# Disentangling surface and bulk properties of Ta_3_N_5_ photoanodes

**DOI:** 10.1038/s41467-026-76117-y

**Published:** 2026-08-11

**Authors:** Lukas M. Wolz, Altantulga Buyan-Arivjikh, Jean Felix Dushimineza, Johannes Dittloff, Laura I. Wagner, Gabriel Grötzner, Jan Luca Blänsdorf, Matthias Kuhl, Sergej Levashov, Julius Kühne, Guanda Zhou, Sonja Matich, Saswati Santra, Verena Streibel, Frans Munnik, Knut Müller-Caspary, Ian D. Sharp, Peter Müller-Buschbaum, Johanna Eichhorn

**Affiliations:** 1 Technical University of Munich, TUM School of Natural Sciences, Department of Physics, Professorship for Nanoscale Microscopy and Spectroscopy of Energy Materials, 85748 Garching, Germany; 2 Technical University of Munich, TUM School of Natural Sciences, Department of Physics, Chair for Functional Materials, 85748 Garching, Germany; 3 Department of Chemistry and Center for NanoScience (CeNS), Ludwig-Maximilians-Universität (LMU) München, Munich, Germany; 4 Technical University of Munich, TUM School of Natural Sciences, Department of Physics, Chair of Experimental Semiconductor Physics, 85748 Garching, Germany; 5 Technical University of Munich, Walter Schottky Institute, 85748 Garching, Germany; 6 Institute of Ion Beam Physics and Materials Research, Helmholtz-Zentrum Dresden-Rossendorf, Dresden, Germany

**Keywords:** Solar fuels, Materials for energy and catalysis

## Abstract

Understanding how material and defect characteristics govern photoelectrochemical performance is essential for developing efficient and stable photoelectrodes. Although bulk properties are often emphasized, surfaces and interfaces can equally determine activity and stability under operation. Here, we use depth-sensitive characterization to disentangle surface and bulk properties of Ta_3_N_5_ thin films. By preparing photoelectrodes from TaO_x_, TaN_x_, and Ta precursors, we systematically vary shallow and deep-level defect concentrations. Structural, compositional, and optoelectronic analyses show that the surfaces consistently exhibit oxygen enrichment, increased structural disorder, and higher deep-level defect densities than the bulk. However, the specific surface structure and its spatial extent depend strongly on precursor chemistry. Ta_3_N_5_ derived from TaO_x_ forms an extended, amorphous, oxide-rich surface with fewer deep-level defects, whereas TaN_x_ and Ta-derived Ta_3_N_5_ films exhibit thinner, more crystalline surfaces with increased mid-gap defect densities. A brief hydrofluoric acid treatment removes the disordered surface layer, improving crystallinity and hydrophilicity and enhancing photoelectrochemical performance and stability for ferrocyanide oxidation. These results highlight interfacial and defect engineering as routes toward durable, high-efficiency Ta_3_N_5_ photoelectrodes.

## Abstract (web summary)

The performance of Ta_3_N_5_ photoanodes depends on the bulk properties, but is also impacted by their surface characteristics. Here, the authors report oxygen-rich, disordered surface layers whose extent depends on the precursor chemistry and show that removing them improves efficiency and stability.

## Introduction

The properties of orthorhombic tantalum nitride (Ta_3_N_5_) and its performance as a photoanode for driving the oxygen evolution reaction (OER) are often dominated by the presence of defects and the incorporation of impurities. Consequently, the photoelectrochemical (PEC) properties strongly depend on the chemical composition of the precursors, the synthesis process, and the subsequent nitridation procedure. For example, Ta_3_N_5_ synthesized via nitridation of tantalum oxide (TaO_x_) shows improved performance and enhanced stability for ferrocyanide oxidation compared to Ta_3_N_5_ synthesized via nitridation of tantalum nitride (TaN_x_) or tantalum (Ta) precursors^1^. Furthermore, increasing nitridation temperatures and ammonia fluxes can reduce the onset potentials and increase the photocurrent densities of such photoanodes^2,3^. Prior studies have shown that these different functional characteristics are caused by the formation of substitutional oxygen impurities (O_N_), nitrogen vacancies (v_N_), or reduced Ta centers (Ta^3+^)^1,4–8^. In particular, both v_N_ and Ta^3+^ act as deep-level defect states that facilitate photocarrier trapping and charge carrier recombination, thus limiting the PEC performance and introducing PEC instabilities^1–4,9–11^. In contrast, O_N_ are shallow donors that can enhance the n-type conductivity and PEC performance characteristics of Ta_3_N_5_^2,9,12,13^. Oxygen impurities can also passivate deep-level defect states, enabling kinetic stabilization for ferrocyanide oxidation and thus, more robust PEC systems^1^.

To manage both the beneficial and detrimental impacts of different defects and impurities in Ta_3_N_5_, intensive efforts have focused on understanding their structural and electronic properties^1–3,14,15^, and developing design strategies that promote charge carrier separation and extraction^4,8,15–21^. However, such studies often focus on the bulk material properties, even though surfaces and interfaces can play an equally important, or even dominant, role in PEC performance and stability^22–24^. Since photogenerated charge carriers must be transported from the bulk to the surface and transferred across the interface, optimizing surface and interface properties is essential for realizing efficient and stable photoelectrodes.

Here, we leverage complementary techniques with different depth sensitivities to reveal the changes between surface and bulk properties of Ta_3_N_5_ thin films. To understand the roles of various defect types and concentrations, we synthesized three different types of Ta_3_N_5_ photoelectrodes with controlled shallow and deep-level defect concentrations using TaO_x_, TaN_x_, and Ta thin films as precursors. Following ammonolysis of these precursor layers to form Ta_3_N_5_ thin films, the structures, chemical compositions, and optoelectronic properties at both the surface and in the bulk were characterized and correlated with their PEC performance. Specifically, grazing incidence wide-angle X-ray scattering (GIWAXS) and depth-dependent confocal photoluminescence (PL) spectroscopy were applied to determine the changes in structure and optoelectronic properties between the bulk and surface of these films. In addition, the chemical compositions in the bulk and at the surface were distinguished by elastic recoil detection analysis (ERDA) and X-ray photoelectron spectroscopy (XPS), respectively. To assess the corresponding (opto)electronic properties, X-ray absorption spectroscopy (XAS) in bulk-sensitive total fluorescence yield (TFY) and surface-sensitive total electron yield (TEY) was performed. This approach enables us to discriminate how crystallinity, surface oxidation, and deep-level defect concentrations at the surface relative to the bulk impact PEC performance and stability. Based on this understanding, we applied a hydrofluoric acid (HF) post-treatment to remove the disordered surface layer, yielding improved PEC onset potentials and increased photocurrent densities. Overall, this work highlights the dominant role of surface properties and how their control can enable significantly enhanced PEC performance characteristics from Ta_3_N_5_.

## Results and discussion

Ta_3_N_5_ photoelectrodes were synthesized with varying chemical compositions to understand the effects of different defect types and concentrations on bulk and surface properties. In brief, we sputter-deposited TaO_x_, TaN_x,_ and Ta thin film precursors, which were subsequently annealed at high temperatures in flowing NH_3_, and are denoted as Ta_3_N_5_(O), Ta_3_N_5_(N), and Ta_3_N_5_(Ta), respectively. For all precursors, homogeneous polycrystalline Ta_3_N_5_ photoelectrodes are formed with a thickness of ~75 ± 20 nm and increasing surface roughness from Ta_3_N_5_(O), to Ta_3_N_5_(N), to Ta_3_N_5_(Ta) (Supplementary Fig. 1). Previously, we reported that all three types of photoelectrodes generate similar photocurrent densities and onset potentials^1^. However, Ta_3_N_5_(N) and Ta_3_N_5_(Ta) were shown to degrade immediately upon exposure to illumination under ferrocyanide oxidation conditions. In general, the stability has been found to strongly depend on the type of hole scavenger^11,25^ and can be enhanced by surface modification and catalyst integration^8,26–29^. Together, these studies reveal the impact of surface and interface properties on PEC characteristics. To understand the underlying reason for this behavior, we now investigate the differences between surface and bulk properties, as well as the specific impacts of synthesis and post-synthetic processing on defining them. To this end, we combine multiple techniques with different probing depths (Supplementary Table 1), with surface-sensitive measurements typically probing the near-surface region within ~5–10 nm. In addition, we note that the transition between surface and bulk properties is not sharply defined and can vary between the three films due to differences in precursor composition, defect distributions, and resulting structural inhomogeneities.

Here, differences between the bulk and surface chemical composition of the differently synthesized films were elucidated by ERDA and XPS, respectively. In the bulk, Ta_3_N_5_(Ta) is characterized by an almost ideal stoichiometry, Ta_3_N_5_(O) by increased oxygen content, and Ta_3_N_5_(N) by increased nitrogen content (Supplementary Table 2). The comparison between XPS and ERDA consistently reveals that the oxygen content is approximately three times higher at the surface than in the bulk across all three films. In detail, we observe decreasing O/Ta ratios for Ta_3_N_5_(O) to Ta_3_N_5_(N) to Ta_3_N_5_(Ta), while the N/Ta ratio simultaneously increases (Supplementary Table 3). As a result, the composition of Ta_3_N_5_(Ta) is closest to the ideal stoichiometry of Ta_3_N_5_, while Ta_3_N_5_(O) is characterized by increased oxygen content and Ta_3_N_5_(N) by slightly increased nitrogen content. Furthermore, formation of an oxygen-rich surface layer is also apparent in the oxygen depth profiles obtained by ERDA (Supplementary Fig. 2a), which reveal a gradually decreasing concentration towards the bulk, in agreement with previous literature^1,30,31^. For ERDA measurements, it is important to note that the gradual increase in element concentrations in the first ~10 nm arises from the limited depth resolution of the detection system, broadening the depth profile. Therefore, we only use ERDA to reveal general trends in the distribution of oxygen and nitrogen near the surface and within the bulk of the films. We observe an increase of near-surface oxygen content relative to bulk, which is most pronounced for Ta_3_N_5_(O) with the highest oxygen content, and is reduced for Ta_3_N_5_(N) and Ta_3_N_5_(Ta), which contain lower oxygen concentrations. In contrast, the nitrogen content exhibits a more homogeneous distribution throughout the bulk. Overall, these results show a pronounced difference between surface and bulk compositions, with these variations depending sensitively on the synthesis route.

**Fig. 1:**
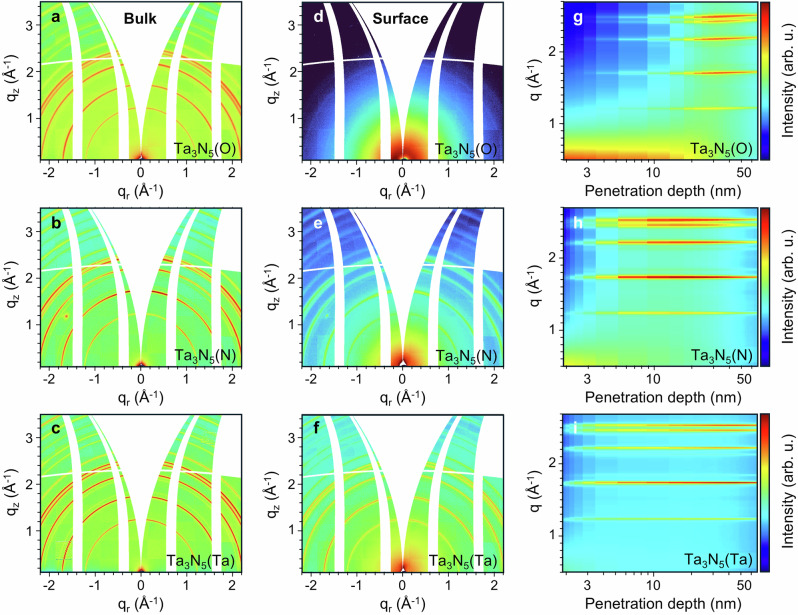
Bulk and surface structures of Ta_3_N_5_ thin films as a function of the different precursors. GIWAXS spectra of Ta_3_N_5_(O), Ta_3_N_5_(N), and Ta_3_N_5_(Ta) using a penetration depth of **a**–**c** 38 nm and **d**–**f** 3 nm, respectively. **g**–**i** Corresponding GIWAXS depth-dependent integrated scattering intensity maps. *q*_r_ corresponds to the in-plane scattering vector component and *q*_z_ to the out-of-plane component. Source data for g-i are provided as a Source data file.

To link the observed compositional changes with the surface and bulk structure of the Ta_3_N_5_ thin films, we next performed GIWAXS measurements as a function of different incident X-ray angles, which correspond to different penetration depths. For a penetration depth of 38 nm (Fig. 1a–c), all three films exhibit distinct and well-defined reflections, confirming the formation of polycrystalline Ta_3_N_5_ in the bulk. In addition, these GIWAXS data show progressively greater broadening of the reflections from Ta_3_N_5_(Ta) to Ta_3_N_5_(N) to Ta_3_N_5_(O), as well as an increasing diffuse scattering background, indicating increasing disorder between these films. Complementary bulk-sensitive grazing incidence X-ray diffraction (GI-XRD) data confirm these observations and additionally reveal shifts in the peak positions (Supplementary Fig. 3)^1,3^ corresponding to small changes in the lattice parameter. In particular, we observe a decrease in the *b*-axis lattice parameter for increasing oxygen content, while the *a-* and *c-*axes only show minor variations between samples. Overall, these findings agree with prior studies that have attributed enhanced lattice disorder and reduced *b*-axis lattice parameters to increased oxygen incorporation in the bulk^1,9,30,32–34^.

For GIWAXS measurements performed at an incident angle corresponding to a penetration depth of 3 nm, Ta_3_N_5_(Ta) still exhibits a clear polycrystalline structure with only minor differences between the surface and bulk. In contrast, the diffraction rings from surface-sensitive GIWAXS measurements are broadened for Ta_3_N_5_(N). Even more strikingly, Ta_3_N_5_(O) is characterized by the formation of an amorphous surface layer (Fig. 1d). These differences are further highlighted by incident angle-dependent GIWAXS maps (Fig. 1g–i), which reveal the structural evolution from the surface to the bulk of each film. While Ta_3_N_5_(Ta) and Ta_3_N_5_(N) exhibit rather crystalline surface layers, the structural differences between surface and bulk are more significant for the case of Ta_3_N_5_(O). In detail, Ta_3_N_5_(O) is characterized by a significantly thicker and more disordered surface layer, extending to greater than 10 nm below the surface, whereas the corresponding surface layer on Ta_3_N_5_(N) is limited to approximately 5 nm.

**Fig. 2:**
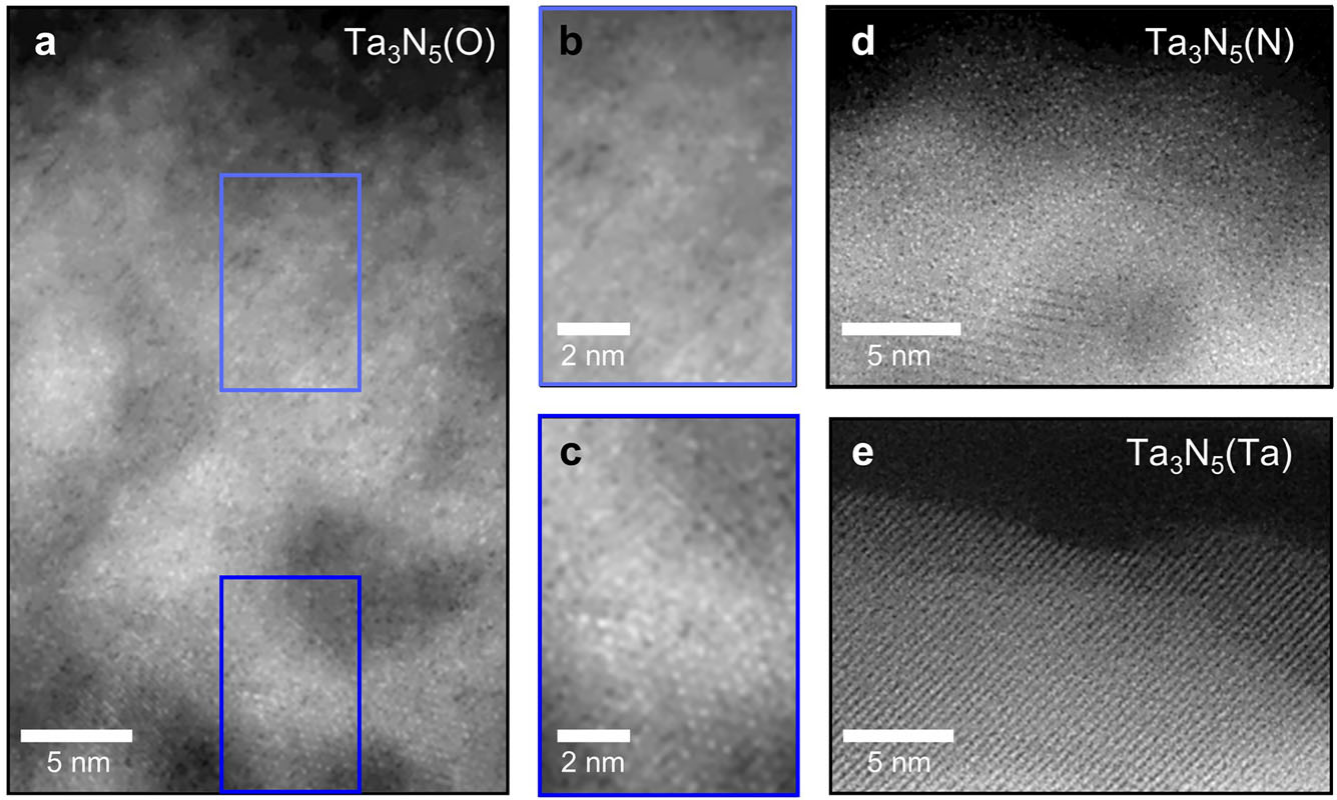
Near-surface structures of Ta_3_N_5_ thin films as a function of the different precursors. Cross-sectional HAADF STEM images of **a**–**c** Ta_3_N_5_(O), **d** Ta_3_N_5_(N), and **e** Ta_3_N_5_(Ta). **b**, **c** Are zoom-in images of the marked areas in (**a**). The black areas at the top of the cross-sections correspond to an evaporated carbon layer. The gray areas correspond to Ta_3_N_5_ and show the transition from the surface (top) towards the bulk (down).

Complementary to GIWAXS, we investigated the near-surface structure of the three photoelectrodes by cross-sectional scanning transmission electron microscopy (STEM). High-angle annular dark field (HAADF) STEM images (Fig. 2) reveal an amorphous surface layer on both Ta_3_N_5_(O) and Ta_3_N_5_(N), with a thickness exceeding 10 nm for Ta_3_N_5_(O) but approximately 5 nm on Ta_3_N_5_(N). In contrast, Ta_3_N_5_(Ta) exhibits a crystalline surface. In this regard, we note that a previous study on Ta_3_N_5_ nanotubes, synthesized by electrochemical anodization of Ta foil and NH_3_ annealing, showed the formation of a crystalline surface structure by TEM^4^. While the present results on films derived from pure Ta are in agreement with this finding, we observe significantly different characteristics for the films synthesized from TaN_x_ and TaO_x_ precursors. The formation of an amorphous surface layer is likely impacted by different synthesis methods, precursor compositions, and conversion conditions, as well as geometric constraints during ammonolysis. For example, the conversion of TaO_x_ into Ta_3_N_5_ is associated with an increase in mass density, leading to a volume contraction, whereas the conversion of TaN_x_ and Ta into Ta_3_N_5_ is characterized by a reduction in mass density, leading to a volume expansion. In addition, thin films are laterally constrained by the substrate and conversion proceeds from the surface, whereas nanostructures, such as nanotubes, have significantly larger gas/solid interface areas from which the transformation can proceed and open internal volumes that can better accommodate density changes. In addition to such factors, comparison of these structural changes with the chemical compositions of the films indicates that increased surface disorder is correlated with increasing oxygen content in the precursor films. Such differences are more evident at the surface compared to the bulk, where the different thin films only exhibit subtle structural variations (Fig. 1). These results provide the demonstration of both structural and chemical deviations between surface and bulk properties in Ta_3_N_5_ as a function of different defect properties and chemical compositions. As discussed below, the increased disorder and higher oxygen contents at the surface can also impact the defect properties and electronic characteristics in both regions.

**Fig. 3:**
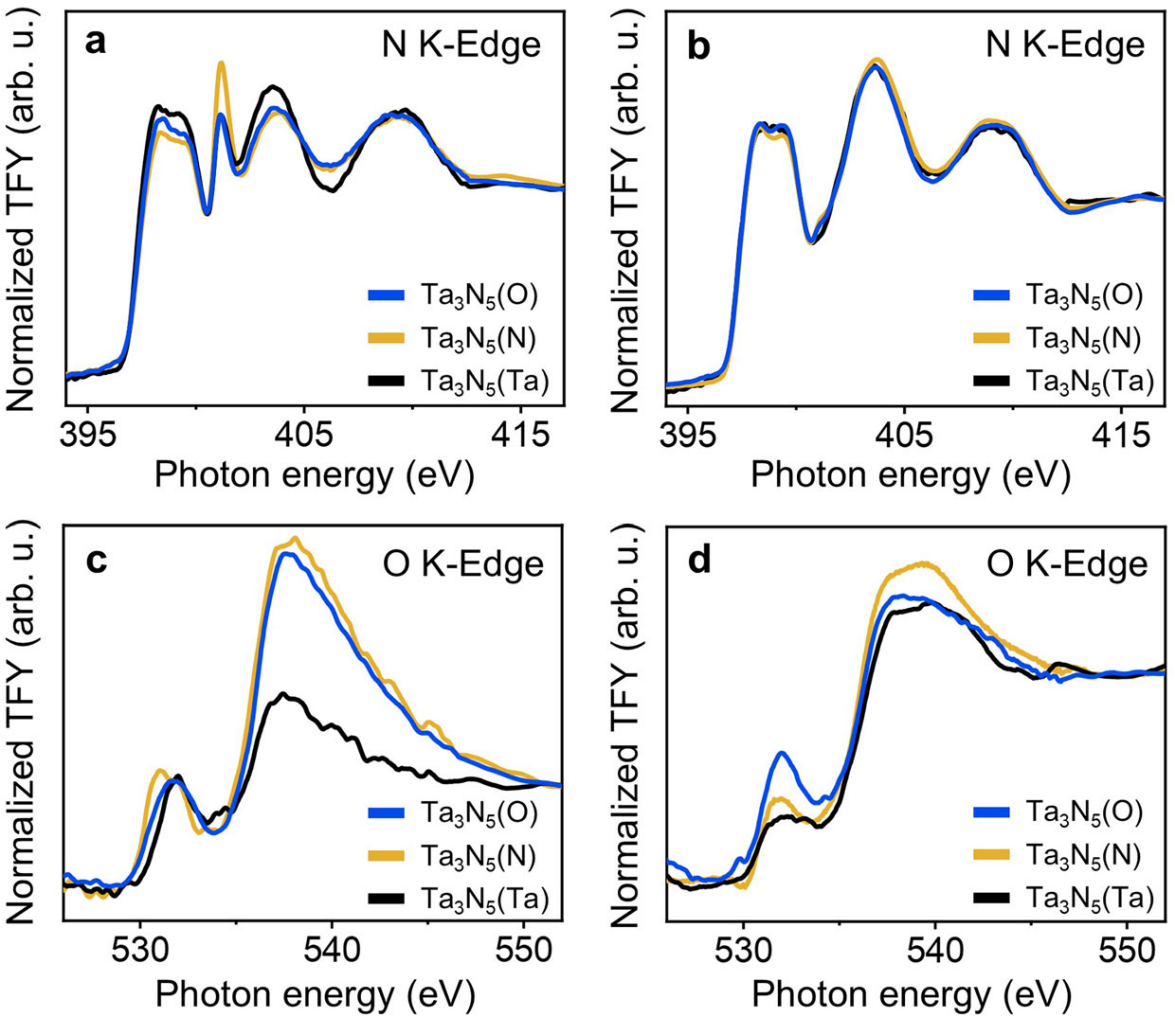
XAS data of the N and O K-edges of Ta_3_N_5_ thin films as a function of the different precursors. Normalized N and O K-edge in **a**, **c** bulk-sensitive total fluorescence yield (TFY) and **b**, **d** surface-sensitive total electron yield (TEY) mode showing the bulk and surface properties of Ta_3_N_5_(O), Ta_3_N_5_(N), and Ta_3_N_5_(Ta), respectively. Source data are provided as a Source data file.

To gain deeper insight into the impact of these different structural properties and chemical compositions on the (opto)electronic properties of Ta_3_N_5_, we next performed N K-edge XAS using both bulk-sensitive TFY and surface-sensitive TEY (Fig. 3) detection modes. In agreement with previous studies^1,4^, the TFY spectra show similar features and absorption onsets, independent of the synthesis pathway, with the exception of the sharp absorption peak located at ~401 eV. This feature can be attributed to dinitrogen incorporated in the lattice^1,4^, and is especially prominent for Ta_3_N_5_(N). This finding is consistent with prior observations that the addition of nitrogen to sputter gas mixtures can result in the incorporation of dinitrogen^35^ that remains present even after NH_3_ annealing^36^. Interestingly, the dinitrogen absorption was significantly weaker in the surface-sensitive TEY spectra (Fig. 3b), indicating that these species are primarily entrapped in the bulk lattice rather than the surface region. Finally, we note that there is no significant change between the different samples in the O K-edge at 532 eV in the bulk (Fig. 3c, d)^37^. In contrast, the surface is characterized by increasing absorption strength at 532 eV from Ta_3_N_5_(Ta) to Ta_3_N_5_(N) to Ta_3_N_5_(O), in agreement with the XPS results above.

**Fig. 4:**
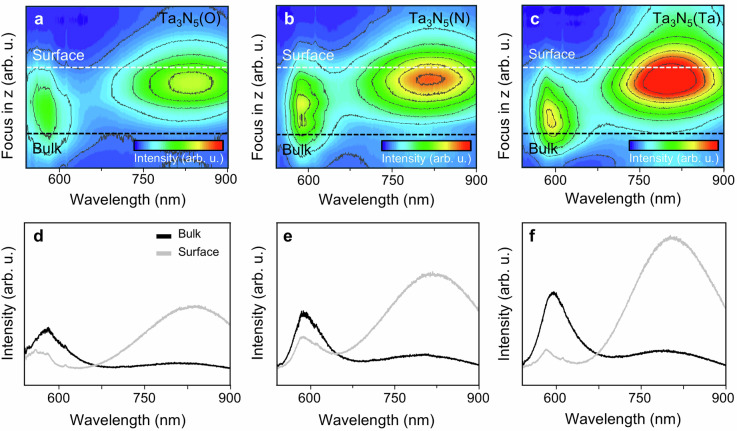
Depth-dependent photoluminescence of Ta_3_N_5_ thin films as a function of the different precursors. **a**–**c** PL intensity map at 10 K as a function of depth (*z*) of Ta_3_N_5_(O), Ta_3_N_5_(N), and Ta_3_N_5_(Ta) excited with a wavelength of 532 nm and a power of 2 mW. **d**–**f** PL spectra extracted from **a** to **c** for surface and bulk regions, indicated by gray and black dotted lines. Source data are provided as a Source data file.

To gain further insights into defect formation and concentrations at the surface and in the bulk, we conducted steady-state confocal PL spectroscopy at low temperatures (10 K). An excitation power of 2 mW (corresponding to ~1.2 × 10^5 ^W cm^−2^) was selected to ensure saturation of emission from defect states for all thin films (Supplementary Fig. 4). As shown in Fig. 4, the depth-dependent optical emission spectra for Ta_3_N_5_(O), Ta_3_N_5_(N), and Ta_3_N_5_(Ta) reveal two peaks for all films, with one more pronounced in the bulk and the other at the surface, independent of the specific film composition.

For optical excitation near surfaces of the films, a broad emission band between 600 nm and 800 nm is observed, indicating the presence of deep-level defect states that have previously been assigned to v_N_ and reduced Ta states^2,17^. We note that prior drive-level capacitance profiling measurements also demonstrated that deep-level defect densities are over an order of magnitude higher at the surface than in the bulk of Ta_3_N_5_^38^. Comparing the sub-bandgap emission intensities among the three films suggests that the defect concentration decreases progressively from Ta_3_N_5_(Ta) to Ta_3_N_5_(N) to Ta_3_N_5_(O). Interestingly, among the three films, Ta_3_N_5_(Ta) displays the strongest emission from deep-level defects at the surface, despite the fact that it is characterized by the highest crystallinity and lowest oxygen content. In contrast, Ta_3_N_5_(O) forms an amorphous surface layer, but with comparatively weak PL from surface defects. In agreement with a previous study^1^, our findings suggest that oxygen incorporation results in greater structural disorder but can also beneficially passivate deep-level defects.

In contrast to the more surface-sensitive measurements discussed above, the PL spectrum from the bulk is dominated by a distinct emission peak at 580 nm, which is consistent with the indirect bandgap of 2.2 eV and has been previously assigned to the band-to-band transition of Ta_3_N_5_^2,3,14^. Near the surface, the strong contribution from deep-levels leads to competitive trapping and recombination, suppressing band-edge and increasing sub-bandgap emission.

In a recent study, we investigated the PEC properties of all three Ta_3_N_5_ films in 1 M potassium phosphate (KP_i_) buffer (pH 12.3) with and without the addition of 0.1 M K_4_Fe(CN)_6_ as a hole scavenger in the dark and under illumination (AM 1.5 G, 100 mW cm^−2^)^1^. Under illumination and in the presence of ferrocyanide, the photocurrent onset potential increased from Ta_3_N_5_(N) to Ta_3_N_5_(Ta) to Ta_3_N_5_(O), while the photocurrent density at 1.23 V vs. reversible hydrogen electrode (V_RHE_) was similar for all photoelectrodes. In contrast, chronoamperometry (CA) measurements revealed rapid degradation of Ta_3_N_5_(N) and Ta_3_N_5_(Ta) during the first 5 min of the experiment in the presence of a hole scavenger, whereas Ta_3_N_5_(O) showed stable operation. Additionally, we observed that all photoelectrodes undergo rapid degradation under water oxidation conditions without a hole scavenger present. The PEC characteristics measured in the present work agree with these prior findings (Fig. 4), and together indicated that they strongly differ between the three photoelectrodes, depending on their material and defect properties, as well as (photo)electrochemical environments.

While the previous studies described above focused on understanding the bulk material properties, it is important to recognize that the surfaces of photoanodes play a critical role in determining the characteristics of solid-liquid junctions. Indeed, we find that PEC performance is directly correlated with the surface properties obtained by PL, GIWAXS, and XPS. In particular, Ta_3_N_5_(N) and Ta_3_N_5_(Ta) surfaces possess lower oxygen contents and improved crystallinity, but also higher deep-level defect concentrations, resulting in rapid degradation. Specifically, larger deep-level defect concentrations near the surfaces of these films result in an unfavorable kinetic competition between interfacial charge injection and trapping-mediated self-oxidation reactions. In contrast, the Ta_3_N_5_(O) surface is characterized by an amorphous surface layer with reduced deep-level defect concentrations, enabling kinetic stabilization of the photoelectrode. These results highlight the importance of understanding defect-mediated recombination processes and approaches to mitigate them for the rational development of interfaces that enable efficient interfacial charge transport and transfer, facilitating high efficiency and long-term stability.

**Fig. 5:**
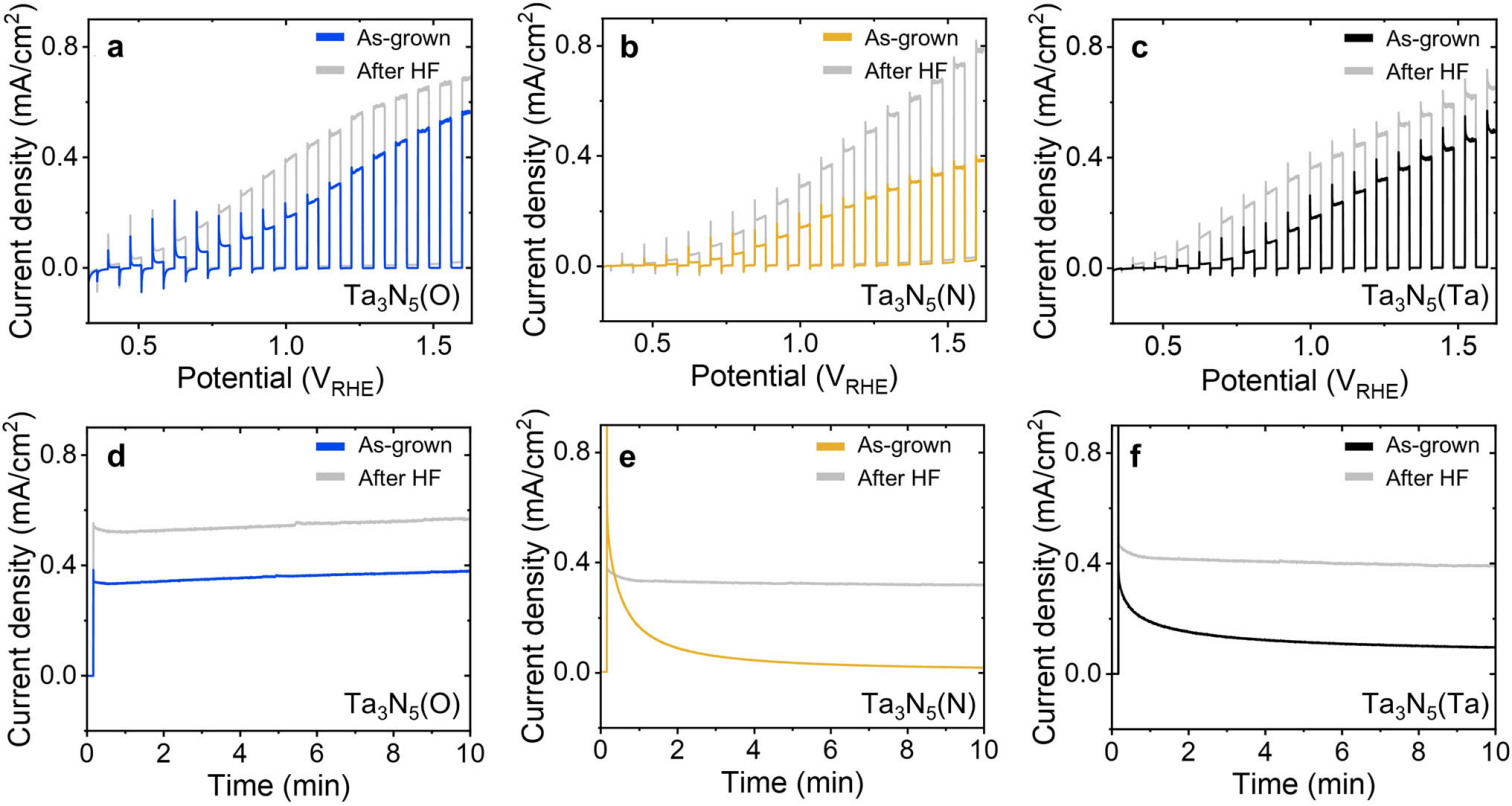
Photoelectrochemical performance characteristics before and after HF surface treatment of Ta_3_N_5_ thin films as a function of the different precursors. Linear sweep voltammetry and chronoamperometry measurements for 10 min at 1.23 V_RHE_ of **a**, **d** Ta_3_N_5_(O), **b**, **e** Ta_3_N_5_(N), and **c**, **f** Ta_3_N_5_(Ta) thin films under chopped illumination in 1 M KP_i_ (pH 12.3) in the presence of 0.1 M K_4_Fe(CN)_6_ as a hole scavenger, before and after the 1 min HF treatment using a three electrode setup. No *iR* correction was applied to the potentials. Source data are provided as a Source data file.

To improve the PEC performance characteristics and stabilities of Ta_3_N_5_, we performed a 1 min post-growth chemical treatment with HF (5%) with the aim of modifying the surface properties by removing the disordered surface layer and/or passivating defects. While HF treatment was already reported to improve the PEC performance of Ta_3_N_5_^28^, the impact on the surface characteristics remains unknown. Here, we include a similar surface treatment to investigate the critical impact on structure, chemical composition, and disorder at the surface, but also to link these surface properties to the PEC characteristics after HF treatment. To this end, linear sweep voltammetry (LSV) was performed in 1 M KP_i_ with 0.1 M K_4_Fe(CN)_6_ under chopped illumination, both before and after the HF treatment (Fig. 5). In agreement with previous reports^28,39^, an enhancement of the photocurrent density and reduction of the onset potential is observed for all films following this HF treatment, while the surface morphology remains similar after HF treatment (Supplementary Fig. 5). Most prominently for Ta_3_N_5_(Ta), the photocurrent transients observed during the LSV are reduced after the treatment, indicating reduced interfacial charge trapping^1,40–44^. The moderate PEC performance observed in this study reflects the deliberate use of model thin films designed to enable comparative mechanistic insights from the different precursor-based synthesis routes. In particular, the film thickness is significantly lower than the ~800 nm typically required to achieve maximum PEC efficiency, and a non-ideal Si back contact was utilized^45^. These choices were made to ensure homogeneous nitridation, enable precise spectroscopic and compositional analysis, and avoid elemental contributions from the support, even though they reduce the light harvesting efficiency and introduce additional back-contact resistances.

Despite the removal of the disordered surface layer following HF etching, differences in PEC performance persist, arising from differences in bulk defect properties associated with the use of three different precursors. As previously reported^1^, Ta_3_N_5_(N) and Ta_3_N_5_(Ta) exhibit comparatively high concentrations of deep-level defects, whereas the higher oxygen content in Ta_3_N_5_(O) facilitates passivation of such states. While deep-level defects lead to rapid photocarrier trapping and recombination, limiting PEC performance, shallow oxygen donors can enhance the electronic conductivity and enable kinetic stabilization of the interface^1^. Consistent with these differences in bulk defect concentrations, Ta_3_N_5_(O) exhibits improved PEC performance and stability compared to Ta_3_N_5_(N) and Ta_3_N_5_(Ta) following HF etching (Fig. 5 and Supplementary Fig. 6). Before HF exposure, only Ta_3_N_5_(O) exhibited stable PEC performance characteristics, while both Ta_3_N_5_(N) and Ta_3_N_5_(Ta) suffered from rapid photocurrent decay (Fig. 5). After HF treatment, all three films show improved durability, which could be indicative of more similar surface and interface properties. Even after 10 h of operation, Ta_3_N_5_(O) still shows stable operation, while a slow decay in the photocurrent density is observed for Ta_3_N_5_(N) and Ta_3_N_5_(Ta). Consequently, enhanced PEC performance and stability are observed for ferrocyanide oxidation even for Ta_3_N_5_(N) and Ta_3_N_5_(Ta) films with high deep-level defect concentrations.

Complementary XPS measurements after 10 h of stability testing reveal changes in the N/Ta and O(530 eV)/Ta ratios (Supplementary Table 4, Supplementary Fig. 7), indicating slight surface oxidation following sustained ferrocyanide oxidation by the HF-treated photoelectrodes. Surface oxidation is mostly pronounced for Ta_3_N_5_(N) and Ta_3_N_5_(Ta) compared to Ta_3_N_5_(O). Comparing XPS spectra of Ta_3_N_5_ photoelectrodes without HF treatment after a 1 h durability test from our previous study^1^ with HF-treated Ta_3_N_5_ photoelectrodes after the 10 h durability test from this study indicates that higher N/Ta ratios are maintained for HF-treated films despite the 10x longer testing duration. Overall, this indicates that HF treatment reduces surface oxidation of Ta_3_N_5_ photoelectrodes. Accordingly, we show that kinetic stabilization of Ta_3_N_5_ can be achieved independent of the exact defect properties by optimizing and tuning the surface, independent of the bulk.

**Fig. 6:**
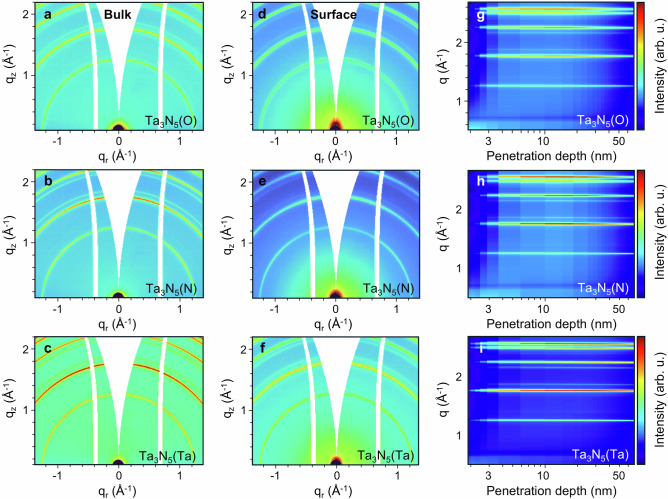
Bulk and surface structures of Ta_3_N_5_ thin films as a function of the different precursors after HF surface treatment. GIWAXS spectra of Ta_3_N_5_(O), Ta_3_N_5_(N), and Ta_3_N_5_(Ta) measured using a penetration depth of **a**–**c** 38 nm and **d**–**f** 3 nm, respectively. **g**–**i** GIWAXS depth-dependent integrated intensity maps. *q*_r_ corresponds to the in-plane scattering vector component and *q*_z_ to the out-of-plane component. Source data for (**g**–**i**) are provided as a Source data file.

To better understand how structural and chemical changes induced by the HF treatment influence the PEC performance characteristics, both the bulk and surface structures were again probed using depth-dependent GIWAXS measurements (penetration depths of 38 nm and 3 nm, respectively). As shown in Fig. 6, while only minimal changes are observed in the bulk, a pronounced sharpening of the near-surface diffraction features is observed after HF treatment of all three photoelectrodes, indicating improved crystallinity at the surface. This effect is most significant for Ta_3_N_5_(O), where distinct reflections emerge after HF treatment compared to the amorphous layer observed for the as-grown material (Fig. 1). In addition, Ta_3_N_5_(N) and Ta_3_N_5_(Ta) show enhanced peak intensities and reduced FWHM at a penetration depth of 3 nm. For all films, the improved crystallinity at the surface after HF treatment is also evident in the integrated intensity maps as a function of incident angle (Fig. 6g–i), indicating reduced thicknesses of the surface layers. To confirm the removal of the surface layer, complementary cross-sectional STEM measurements were conducted on Ta_3_N_5_(O) after HF treatment (Supplementary Fig. 8). Compared to as grown Ta_3_N_5_(O), the measurements confirm that the amorphous layer is significantly reduced after HF etching and the near-surface crystallinity is improved.

Additionally, XPS analysis of the O 1*s*, N 1*s*, and Ta 4*f* core levels of all three Ta_3_N_5_ films provides additional insight into the chemical surface modifications induced by HF treatments (Supplementary Figs. 9–11). For Ta 4*f* and N 1*s*, we considered the contribution of all formed species, while for O 1*s*, only the peak at ~530 eV was considered (O(530 eV)), corresponding to Ta-O bonds and thus to oxygen incorporated in Ta_3_N_5_. For Ta_3_N_5_(O), a reduction in the O(530 eV)/Ta ratio and a slight increase in the total N/Ta ratio are observed. Interestingly, after HF treatment, Ta_3_N_5_(N) and Ta_3_N_5_(Ta) possess similar O(530 eV)/Ta ratios within the detection limit of our system, while the N/Ta ratio is slightly reduced, reaching values that are similar to that of Ta_3_N_5_(O) (Supplementary Table 5). The subtle changes in the surfaces of these two films are consistent with their more ordered, thinner surface layers directly after synthesis, while the similarity in N content agrees with their more similar surface structures following the HF treatment. Furthermore, we do not observe significant changes in the core level binding energies, which agrees with previous XPS observations from Ta_3_N_5_ surfaces treated with a mixed etchant (HF/HNO_3_/H_2_O = 1:2:7)^2^.

Furthermore, we analyzed the F 1*s* and O 1*s* to better understand the surface properties of the different Ta_3_N_5_ thin films. In the F 1 *s* spectra (Supplementary Figs. 12 and 13), we observed the presence of fluoride ions only after HF treatment for all films. In the O 1*s* spectra, hydroxyl groups and exposed regions of silicon terminated by sub-oxides have overlapping signals at ~532 eV. To understand the presence of hydroxyl groups on the surface, the contributions need to be deconvoluted (Supplementary Table 6). The remnant photoemission intensity after subtracting the silicon oxide contribution suggests that hydroxyl groups are also present at the surface after HF treatment. Furthermore, we analyzed the chemical composition after PEC testing for 10 h and immersion for 1 min in 1 M KP_i_ with hole scavenger (Supplementary Figs. 12 and 13), which shows the fast disappearance of the F 1 *s* contribution on the surface, while the stability is still improved. These findings indicate that fluoride ion absorption on the surface is not the main reason for the observed stabilization.

To understand how the termination impacts the surface properties, we performed contact angle measurements with deionized water before and after HF treatments (Supplementary Fig. 14). Directly after nitridation, the photoelectrodes are relatively hydrophobic, with contact angles ranging from 80° to 100° across different defect types. These findings agree with a previous report on oxide-to-nitride converted Ta_3_N_5_^46^. After HF treatment, the contact angles are significantly reduced by ~40° for all three thin films, indicating enhanced surface hydrophilicity. Before and after HF treatment, Ta_3_N_5_(N) and Ta_3_N_5_(Ta) exhibit rather similar contact angles, whereas Ta_3_N_5_(O) exhibits slightly higher values. In general, it has been shown that the surface hydrophilicity can significantly impact the PEC activity and stability^47^. For example, improved hydrophilicity can facilitate interfacial interactions between the electrode and electrolyte, accelerate photogenerated charge carrier transfer and separation^48^, and increase the gas release rate^49^. Consequently, the improved hydrophilicity provides an essential contribution to the improved PEC performance after HF treatment.

Additionally, to determine the change in defect properties after HF treatment, highly sensitive optical absorption measurements were performed via photothermal deflection spectroscopy (PDS) before and after HF treatment (Supplementary Fig. 15). Ta_3_N_5_(O) and Ta_3_N_5_(N) are characterized by a small reduction in the sub-bandgap absorption, while Ta_3_N_5_(Ta) exhibits rather similar absorption before and after HF treatment. Accordingly, we conclude that removal of the disordered surface layer leads to partial elimination of mid-gap defect states. To link changes in surface properties to charge transport/transfer characteristics, we performed both electrochemical impedance spectroscopy (EIS) and photoelectrochemical impedance spectroscopy (PEIS) on Ta_3_N_5_(O) thin films with and without HF treatment in 1 M KP_i_ and 0.1 M K_4_Fe(CN)_6_ as a hole scavenger. In detail, we recorded EIS over a potential range from −0.1 V_RHE_ to 1.6 V_RHE_ and a frequency range from 1 Hz to 100 kHz (Supplementary Fig. 16). The data were fitted with an equivalent circuit to describe Ta_3_N_5_ in contact with the electrolyte (Supplementary Fig. 16c). The assignment of the circuit elements, as shown below, is consistent with previous EIS interpretations for Ta_3_N_5_ in literature^21,50,51^. We used the fitted capacitances from broadband EIS to derive the Mott–Schottky plot (C^−2^ vs. V_RHE_) of Ta_3_N_5_(O) with and without HF treatment. For as-grown Ta_3_N_5_(O), we observe a two-slope behavior, with a change in slope at higher applied potentials (Supplementary Fig. 16d). This behavior, previously observed in Ta_3_N_5_, suggests that band bending responds to the applied potential to varying degrees across different potential regions^21^. In the low potential region, the response is consistent with Fermi level pinning due to the existence of a distribution of surface states. At more anodic potentials, partial depinning of the Fermi level leads to a stronger dependence of the band bending on the potential, corresponding to a depletion-dominated regime^21^. After HF treatment, the Mott–Schottky response approaches the single-regime behavior over the investigated potential range. Complementary PEIS measurements show that the radius of the semicircle decreases at comparable applied potentials after HF treatment (Supplementary Fig. 17), indicating a lower overall resistance under operating conditions and more favorable photoelectrochemical charge-transfer characteristics.

Overall, the improved PEC characteristics are consistent with the GIWAXS results, which show that HF treatment removes the disordered oxide layer. This structural and chemical modification of the surface is especially pronounced in Ta_3_N_5_(O) photoelectrodes. Importantly, the effect of HF extends beyond the elimination of disordered surface layers by also increasing the hydrophilicity of the surface. Together, these changes could lead to fewer electronic trap states, diminished charge carrier recombination, and more efficient interfacial charge transfer. The observed enhancement in photocurrent performance confirms that surface defects and disorder limit efficient hole transfer, and that even a brief HF treatment can significantly improve the interfacial properties of Ta_3_N_5_ photoanodes.

In summary, we systematically investigated how thin film precursor selection impacts defect formation and distribution in Ta_3_N_5_ photoelectrodes, both at the surface and in the bulk. Using a combination of depth-sensitive characterization methods, including GIWAXS, XAS, ERDA, and PL, we identified clear differences between surface and bulk characteristics as a function of the defect properties. The surfaces of all films exhibited pronounced oxygen enrichment along with higher structural disorder and increased concentrations of deep-level defects compared to their bulk counterparts. However, the chemical nature of the precursor strongly influences these characteristics, with oxygen-rich precursors forming amorphous oxide-rich surfaces that exhibit fewer deep-level defects. In contrast, Ta precursors produced crystalline surfaces, but with increased densities of mid-gap defect states. Removing the disordered surface layer using a brief hydrofluoric acid treatment leads to improved surface crystallinity and increased hydrophilicity, as well as reduced oxygen content. Consequently, enhanced PEC performance and stability are observed for ferrocyanide oxidation even for Ta_3_N_5_ films with large deep-level defect concentrations. These findings highlight the necessity of independently optimizing surface and bulk properties to effectively enhance the efficiency and stability of Ta_3_N_5_-based photoelectrodes.

## Methods

### Synthesis of Ta_3_N_5_ thin films

All precursor thin films were deposited using reactive magnetron sputtering (PVD 75, Kurt J. Lesker Company). The system has a base pressure of 5 × 10^−8 ^Torr and a target-substrate distance of ~17 cm. The thin films were simultaneously sputtered onto n^+^-type doped Si wafers (As-doped, (100) oriented, Siegert Wafer GmbH) for PEC characterization and onto fused silica substrates (Siegert Wafer GmbH) for optical characterization. Prior to deposition, the fused silica substrates were cleaned with deionized water, acetone, and isopropanol, followed by drying with flowing nitrogen.

For target conditioning, the Ta target (99.95%, Kurt J. Lesker Company) was initially sputter-cleaned using a 60 W DC Ar plasma (99.9999%, Linde GmbH) for 15 min at a pressure of 8.5 mTorr, with the substrate shutter closed. During the final 5 min of this process, a 20 W radio frequency (RF) substrate bias was applied to simultaneously sputter-clean the substrates in-situ before deposition. For the deposition of TaO_x_ and TaN_x_, the Ta target was sputtered in a gas mixture composed of Ar, N_2_ (99.9999%, Linde GmbH), and O_2_ (99.9999%, Linde GmbH). The TaO_x_ films were deposited for 13 min with an average power of 50 W, a substrate setpoint temperature of 500 °C, and a process pressure of 8.5 mTorr in an Ar atmosphere containing 10% O_2_ as the reactive gas. To prevent substrate oxidation before TaO_x_ deposition, a thin Ta interlayer (5 nm) was sputtered for 2 min with a power of 60 W, substrate setpoint temperature of 500 °C, and a process pressure of 8.5 mTorr, using an Ar flow rate of 40 sccm. The TaN_x_ films were deposited for 70 min with an average power of 50 W, operating in active pulsed mode at 100 kHz with a 98% duty cycle, a substrate setpoint temperature of 650 °C, and a process pressure of 7 mTorr. The gas flow rates for Ar, N_2_, and O_2_ were set to 10 sccm, 20 sccm, and 0.55 sccm, respectively. Finally, Ta films were deposited for 18 min with a direct current (DC) power of 60 W, a substrate setpoint temperature of 400 °C, and a process pressure of 5.5 mTorr using an Ar flow rate of 25 sccm. During all deposition processes, the substrate holder was rotated at 10 rpm.

After deposition, the precursor thin films were annealed in a quartz tube furnace (Nabertherm RS 80/300/1) under a constant NH_3_ (99.999%, Linde GmbH) flow rate of 100 sccm at 1 bar. The temperature was ramped at a rate of 30 °C min^−1^. Upon reaching a target temperature of 920 °C, the samples were held at this temperature for 3 h. The furnace was turned off, and the thin films were allowed to cool under continuous NH_3_ flow. To expedite cooling, the furnace was opened when the temperature dropped below 400 °C. Upon reaching room temperature, the gas flow was switched to pure N_2_, and the tube was purged for 10 min before removing the films. For HF post-treatment, the surface was modified by immersing the Ta_3_N_5_ photoelectrodes in a 5% HF solution for 1 min, following by rinsing in de-ionized water and drying under flowing N_2_.

### Structural and morphological characterization

The crystal structures and phase purities of the films were analyzed by GI-XRD using a Rigaku SmartLab system with Cu K_α_ radiation at a 0.5° incidence angle. The 2*θ* diffraction angle was measured from 15° to 70° in increments of 0.05°.

GIWAXS experiments were performed using a Pilatus 300k (Dectris) detector at the P03 MiNaXS beamline at the PETRA III synchrotron DESY, Hamburg^52^. The measurements were conducted with an X-ray energy of 12.12 keV and incidence angles ranging from 0.1° to 0.8° in 0.025° increments to achieve depth-dependent GIWAXS analysis. Image processing was carried out using the Python tool INSIGHT^53^.

The X-ray penetration depths $\tau \left(\lambda \right)$ in GIWAXS were calculated according to ref. ^54^.

1 $$\tau \left(\lambda \right)=\frac{\lambda }{4\pi }{\left(\frac{2}{\sqrt{{\left({\alpha }_{{{{\rm{i}}}}}^{2}-{\alpha }_{{{{\rm{c}}}}}^{2}\right)}^{2}+4{\beta \left(\lambda \right)}^{2}}-\left({\alpha }_{{{{\rm{i}}}}}^{2}-{\alpha }_{{{{\rm{c}}}}}^{2}\right)}\right)}^{1/2}$$

where *λ* is the X-ray wavelength, *α*_i_ the incidence angle, *α*_c_ the critical angle, and *β(λ)* the absorption term of the complex refractive index:

2 $$n=1-\delta \left(\lambda \right)+i\beta \left(\lambda \right)$$

with $\delta \left(\lambda \right)$ corresponding to the dispersion factor of the complex refractive index. Dispersion- and absorption factors were calculated via^55^:

3 $$\delta \left(\lambda \right)=\frac{\rho {N}_{{{{\rm{A}}}}}}{{M}_{{{{\rm{a}}}}}}\frac{{r}_{{{{\rm{e}}}}}{\lambda }^{2}}{2\pi }\sum {f}^{{\prime} }$$

4 $$\beta \left(\lambda \right)=\frac{\rho {N}_{{{{\rm{A}}}}}}{{M}_{{{{\rm{a}}}}}}\frac{{r}_{{{{\rm{e}}}}}{\lambda }^{2}}{2\pi }\sum {f}^{{\prime} {\prime} }$$

where ρ corresponds to the respective sample density, ${M}_{{{{\rm{a}}}}}$ its molecular mass, ${r}_{{{{\rm{e}}}}}$ the classical electron radius, and ${f}^{{\prime} }$ and ${f}^{{\prime} {\prime} }$ the real and imaginary parts of the atomic scattering factor obtained from values provided by Henke et al.^56^. The critical angle of each sample was calculated as:

$${\alpha }_{{{{\rm{c}}}}}=\sqrt{2\delta \left(\lambda \right)}$$

STEM measurements were performed at an FEI Titan Themis 80–300 (S)TEM, operated at 200 kV, equipped with an XFEG field emission gun, the aberration corrector CEOS DCOR and the chemiSTEM SuperX detection system for energy-dispersive X-ray spectroscopy. HAADF STEM images were acquired using a semi-convergence angle of the scanning probe of 20.0 mrad and a Fischione Model 3000 ring detector covering scattering angles of 35–200 mrad at the used camera length of 195 mm. TEM-lamellae were prepared by a standard milling and lift-out procedure, followed by thinning and polishing steps, inside an SEM FEI Helios NanoLab G3 UC equipped with the MultiChem gas delivery system using a Pt-source and a Tomahawk ion column generating a focused Ga-ion beam. TEM-lamellae were cut from sputtered films grown on Si wafers and attached to molybdenum FIB lift-out grids for TEM usage. Before preparation, an approximately 20 nm carbon film was evaporated onto each wafer using the compact-coating-unit-010.

For SEM imaging and focused ion beam (FIB) preparation, a Zeiss Crossbeam 550-2 KMat was used (FE-REM with GEMINI II electron optics). SEM images were acquired at a low acceleration voltage to reduce charging effects (InLens for top view and SE2 for cross-section view; 5 kV and *I*_probe_ 100 pA in both cases). Before cutting the cross-section, the sample was protected with a thin Pt layer formed by electron beam induced deposition (1 kV, 5 nA, 1250 mC cm^−2^, track and pixel spacing 10 nm, dwell time 0.5 µs, cycles 1, reference height 120 nm). The scanning mode was fast bidirectional, and the cycle mode was back-and-forth. The subsequent cutting steps were performed with 30 kV Ga-ions (50 pA, followed by a 10 pA polishing step). Furthermore, a dose of 200 mC cm^−2^, a pixel and track spacing of 2.25 nm (1.25 nm for polishing), and a dwell time of 20.25 µs (31.25 µs for polishing) were applied. In both cases, we used 10 cycles and a reference depth of 1000 nm. The scanning mode was fast bidirectional, and the cycle mode was cross-sectional.

### Compositional characterization

ERDA measurements were conducted at the Ion Beam Centre of the Helmholtz-Zentrum Dresden-Rossendorf. A 43 MeV Cl^7+^ ion beam was used to determine the bulk composition of the thin film photoelectrodes. The incident beam was oriented at 75° relative to the sample normal and the scattering angle was 30°. An area of approximately 1.5 × 1.5 mm^2^ was analyzed. Recoiled atoms and scattered ions were recorded using a Bragg ionization chamber. Hydrogen recoils were measured separately with a solid-state detector positioned at a scattering angle of 40°. A 25 µm Kapton foil placed in front of this detector blocked scattered ions and heavy recoil ions. The depth resolution of this system was limited due to energy-loss straggling in the foil. Windf v9.3 g was used to fit the spectra^57^. The obtained depth profiles were used to determine the absolute elemental concentrations.

XPS was employed to characterized the near-surface composition using a SPECS system equipped with either a monochromatized or polychromatic Al K_α_ source (*hν* = 1486.6 eV). Spectra were acquired with a pass energy of 20 eV. Owing to variations in the surface properties, the position of the C=C peak component was changed and, consequently, was not sufficiently reliable for energy calibration. Instead, the binding energies were corrected by applying a constant shift of 0.7 eV to prevent artificial variations between the thin films. This correction was obtained from the average offset required to align the Ta 4*f*_7/2_ peak at 24.8 eV for all films, consistent with previous results^1,3^. Peak fitting was performed using the CasaXPS software. Surface atomic composition of the annealed Ta_3_N_5_ films was calculated from the peak areas of the N 1*s*, Ta 4*f*_7/2_, O 1*s*, and Si 2*p* core levels using the corresponding atomic sensitivity factors together with effective attenuation length escape depth correction.

### Optical characterization

PL spectroscopy was performed with an Andor Kymera spectrograph equipped with a 300 lines mm^−1^ grating and a focal length of 328 mm focal length, coupled to an Andor iDus 416 CCD detector. A confocal optical configuration with a 20×/NA 0.45 objective was used, enabling out-of-plane measurements. The excitation wavelength was 403 nm and a 410 nm long-pass filter was used. All PL measurements were conducted in a liquid-flow cryostat under vacuum conditions at pressures below 1 × 10^−5 ^mbar, with temperature varied between 10 K and 300 K. Depth-dependent PL emission maps were acquired by gradually shifting the focal plane from below to above the sample while simultaneously recording spectra at 0.1 s intervals. The resulting datasets were subsequently treated with an FFT-based filter to reduce noise caused by mechanical vibrations.

XAS measurements at the N K-edge were performed at the BEAR beamline of IOM-CNR at Elettra. All thin film photoelectrodes were analyzed in TFY and TEY modes. The N K-edge spectra were collected over three energy ranges: 390 eV to 402 eV with a step size of 0.25 eV, 402 eV to 423 eV with a step size of 0.05 eV, and 423 eV to 460 eV with a step size of 0.2 eV. Three consecutive scans were recorded using an integration time of 0.4 s per data point. The incident X-ray intensity was monitored before and after analysis of each thin film photoelectrode. Data processing was conducted with Origin to subtract pre- and post-edge contributions and to normalize the spectra to the edge step. Energy calibration was performed using the second derivative of the corresponding reference absorption spectrum.

PDS was employed to quantify absorption both above- and below the bandgap using a home-built setup. The photoelectrodes were immersed in perfluorohexane and illuminated at normal incidence with monochromated light from either a halogen or xenon lamp, modulated at 11 Hz. Absorbance was measured from 350 nm to 1300 nm by monitoring the deflection of a 635 nm laser-diode probe beam on a segmented detector. The detector signal was demodulated using a SR530 lock-in amplifier (Stanford Research Systems).

### Photoelectrochemical characterization

Working electrodes were prepared by mechanically scratching the rear side of the Si substrate and applying a conductive indium-gallium eutectic (Sigma-Aldrich) to form the electrical contact. A silver epoxy (CW 2400, Circuit Works) was used to connect a copper wire to the Si substrate. To isolate the connection from the electrolyte, the wire was enclosed in a glass tube and sealed with a silicone polymer compound (101RF BLACK, Microset). The area of the electrodes was between 0.5 cm^2^ to 1.0 cm^2^. The PEC performance characteristics and stability of Ta_3_N_5_ thin films were evaluated by linear sweep voltammetry and chronoamperometry using a Biologic SP-300 potentiostat. Measurements were carried out in a three-electrode PEC cell (Supplementary Fig. S6a) comprising the Ta_3_N_5_ photoanode as the working electrode, a Pt wire as counter electrode, and an Ag/AgCl reference electrode (3 M KCl, Dri-Ref-2SH, WPI Europe). The Ag/AgCl reference electrode was calibrated against a master reference electrode. All PEC experiments were conducted in a 1 M KP_i_ at pH 12.3 ± 0.1 with 0.1 M K_4_Fe(CN)_6_ as a sacrificial hole acceptor (40 ml). The 1 M KP_i_ electrolyte was prepared by dissolving 8.7 g K_2_HPO_4_ and 10.7 g K_3_PO_4_ in distilled water and adjusting the final volume to 100 ml. The KP_i_ electrolyte was stored in a tightly sealed bottle at room temperature (average 23 °C) until use. For measurements employing a hole scavenger, 4.22 g K_4_Fe(CN)_6_·were dissolved in 100 ml of the KP_i_ solution right before the experiment. Before each PEC measurement, the electrolyte was purged with high-purity Ar (99.9999%, Linde GmbH) for 10 min. Front-side illumination was provided by an AM 1.5 G solar simulator (Asahi) calibrated to an irradiance of 100 mW cm^−2^. Linear sweep voltammograms were recorded between 0.4 V and 1.6 V_RHE_ at a scan rate of 50 mV s^−1^ under continuous or chopped illumination, as well as dark conditions. Stability measurements were performed by chronoamperometry at an applied potential of 1.23 V_RHE_ under continuous illumination. The potential of the Ag/AgCl reference electrode was verified against a commercial Ag/AgCl reference electrode. All measured potentials were referenced to a reversible hydrogen electrode according to *E*_RHE_ = *E*_Ag/AgCl_ + 0.210 V + (0.0591 V × pH). Photocurrent densities were obtained by dividing the measured current by the geometrically illuminated area of the photoelectrode.

Mott–Schottky analysis was carried out in the dark over a potential range of –0.1 V_RHE_ to 1.9 V_RHE_ and a frequency range from 1 Hz to 100 kHz. The equivalent-circuit framework comprises the series resistance *R*_series_, bulk (*Q*_bulk_, *R*_bulk_) and interface (*C*_inter_, *R*_inter_) contributions (Supplementary Fig. 16c). The Mott–Schottky plots were constructed from capacitances obtained by fitting the full impedance spectra at each potential. PEIS was performed in 1 M KP_i_ with 0.1 M K_4_Fe(CN)_6_ under illumination using a potential range from −0.4 V_RHE_ to 1.6 V_RHE_ and a frequency range from 1 Hz to 100 kHz.

## Supplementary information

Supplementary Information

Transparent Peer Review file

## Source data

Source Data

## Data Availability

Source data are provided with this paper.
