# Supplementary material for: Disentangling surface and bulk properties of Ta3N5 photoanodes
Source: Nat Commun. 2026 Aug 11;17:7773. doi: 10.1038/s41467-026-76117-y (PMC13463003; doi:10.1038/s41467-026-76117-y)
Supplement: Supplementary file 1 [file 41467_2026_76117_MOESM1_ESM.pdf]

Supplementary Information

Disentangling surface and bulk properties of Ta_3N_5 photoanodes

Lukas M. Wolz,[1] Altantulga Buyan-Arivjikh,[2] Jean Felix Dushimineza,[3] Johannes Dittloff,[4,5] Laura I. Wagner,[4,5] Gabriel Grötzner,[4,5] Jan Luca Blänsdorf,[1] Matthias Kuhl,[1] Sergej Levashov,[1] Julius Kühne,[4,5] Guanda Zhou,[4,5] Sonja Matich,[4,5] Saswati Santra,[4,5] Verena Streibel,[4,5] Frans Munnik,[6] Knut Müller-Caspary,[3] Ian D. Sharp,[4,5] Peter Müller-Buschbaum,[2] and Johanna Eichhorn[1*]

[1] Technical University of Munich, TUM School of Natural Sciences, Department of Physics, Professorship for Nanoscale Microscopy and Spectroscopy of Energy Materials, 85748 Garching, Germany
[2] Technical University of Munich, TUM School of Natural Sciences, Department of Physics, Chair for Functional Materials, 85748 Garching, Germany
[3] Ludwig-Maximilians-Universität (LMU) München, Department of Chemistry and Center for NanoScience (CeNS), 81377 Munich, Germany
[4] Technical University of Munich, TUM School of Natural Sciences, Department of Physics, Chair of Experimental Semiconductor Physics, 85748 Garching, Germany
[5] Technical University of Munich, Walter Schottky Institute, 85748 Garching, Germany
[6] Helmholtz-Zentrum Dresden-Rossendorf, Institute of Ion Beam Physics and Materials Research, 01328 Dresden, Germany

Supplementary Table 1 Depth resolution of the different techniques used for surface and bulk material characterization in this work.

	Surface	Bulk	Reference
Chemical composition	**XPS**<br>~ 5 nm	**ERDA**<br>Signal from complete film<br>Resolution ~10 nm	[1,2]<br>[3]
Structure	**GIWAX**<br>~ 3 nm	**GIWAX**<br>~ 38 nm	[4,5]
XAS	**TEY**<br>Element-dependent:<br>~ 10 nm	**TFY**<br>Multiple hundred nm	[6,7]
PL	Focus point near surface	Focus point in bulk	[8]

Supplementary Table 2 Atomic ratios of O/Ta, N/Ta, and N/O derived from ERDA for all thin films directly after synthesis.

Sample	O/Ta	N/Ta
Ta_3N_5(O)	0.10	1.59
Ta_3N_5(N)	0.07	1.75
Ta_3N_5(Ta)	0.05	1.68

Supplementary Table 3 Atomic ratios of Ta-O/Ta and N/Ta derived from XPS core level spectra (O 1*s*, N 1*s*, Ta 4*f*) for all thin films directly after synthesis. Additionally, the relative contributions of TaN_x and TaO_xN_y obtained from the Ta 4*f* spectra are shown. For the oxygen content, only the peak at 530 eV, corresponding to Ta-O bonds, was considered.

Sample	Ta-O/Ta	N/Ta	TaN_x (at.%)	TaO_xN_y (at.%)
Ta_3N_5(O)	0.28	1.38	87.6	12.4
Ta_3N_5(N)	0.11	1.65	92.6	7.4
Ta_3N_5(Ta)	0.11	1.70	93.0	7.0

Supplementary Table 4 Atomic ratios of Ta-O/Ta and N/Ta derived from XPS core level spectra (O 1*s*, N 1*s*, Ta 4f) for all thin films directly after synthesis. Additionally, the relative contributions of TaN_x and TaO_xN_y obtained from the Ta 4*f* spectra are shown. For the oxygen content, only the peak at 530 eV, corresponding to Ta-O bonds, was considered.

Sample	Ta-O/Ta	N/Ta	TaN_x (at.%)	TaO_xN_y (at.%)	TaO_x (at.%)
Ta_3N_5(O)	0.45	1.22	76.6	17.2	6.2
Ta_3N_5(N)	1.01	0.85	65.1	19.0	15.9
Ta_3N_5(Ta)	1.32	0.73	55.1	31.8	13.1

Supplementary Table 5 Atomic ratios of Ta-O/Ta, N/Ta, and N/O-Ta derived from XPS core level spectra (O 1*s*, N 1*s*, Ta 4*f*) for all thin films after HF treatment. Additionally, the relative contributions of TaN_x and TaO_xN_y obtained from the Ta 4*f* spectra are shown. For the oxygen content, only the peak at 530 eV, corresponding to Ta-O bonds, was considered.

Sample	Ta-O/Ta	N/Ta	TaN_x (at.%)	TaO_xN_y (at.%)
Ta_3N_5(O)	0.21	1.40	87.9	12.1
Ta_3N_5(N)	0.14	1.43	89.2	10.8
Ta_3N_5(Ta)	0.14	1.45	89.5	10.5

Supplementary Table 6 Atomic ratio of O(532 eV)/Si derived from XPS core-level spectra (O 1*s*, Si 2*p*) for all thin films after synthesis and after HF treatment. Therefore, only the Si 2p and O 1s (532 eV) peaks were considered. O 1*s* (532 eV) is typically assigned to hydroxyl groups at the surface, but it can also contain contributions from uncovered regions of the substrate terminated by silicon sub-oxides. After HF treatment, we observe a significant decrease in the intensity of the Si-O component in Si 2*p*.

Sample	As-grown			After HF treatment		
	O(at.%)	Si(at.%)	O(532 eV)/Si	O(at.%)	Si(at.%)	O(532 eV)//Si
Ta_3N_5(O)	69	31	2.2	84	16	5.3
Ta_3N_5(N)	56	44	1.3	75	25	3
Ta_3N_5(Ta)	56	44	1.3	75	25	3

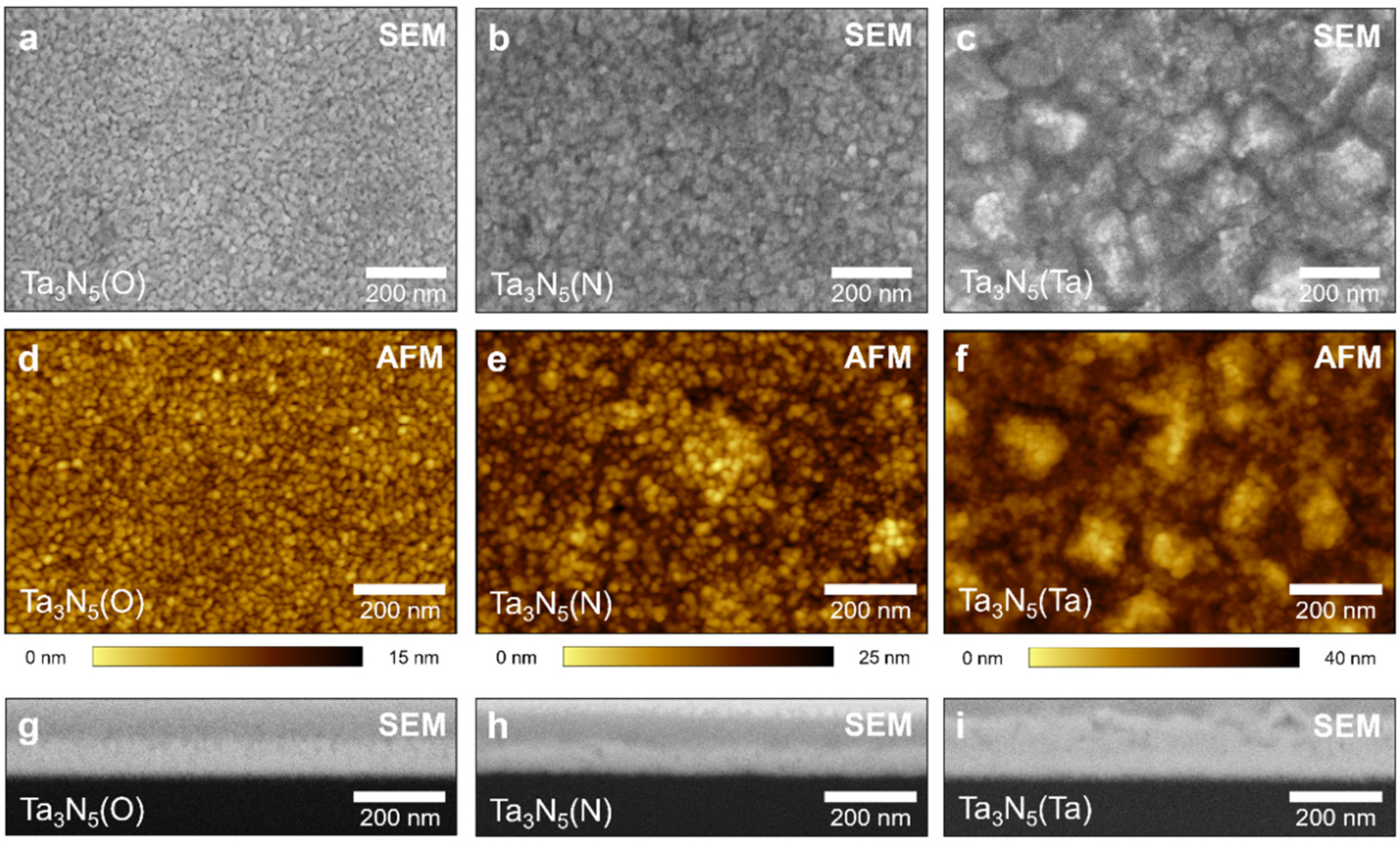

Supplementary Fig. 1 Top-view SEM **a-c** and AFM measurements of **d-f** Ta_3N_5(O), Ta_3N_5(N), and Ta_3N_5(Ta), respectively. **g-i** Corresponding cross-sectional SEM images.

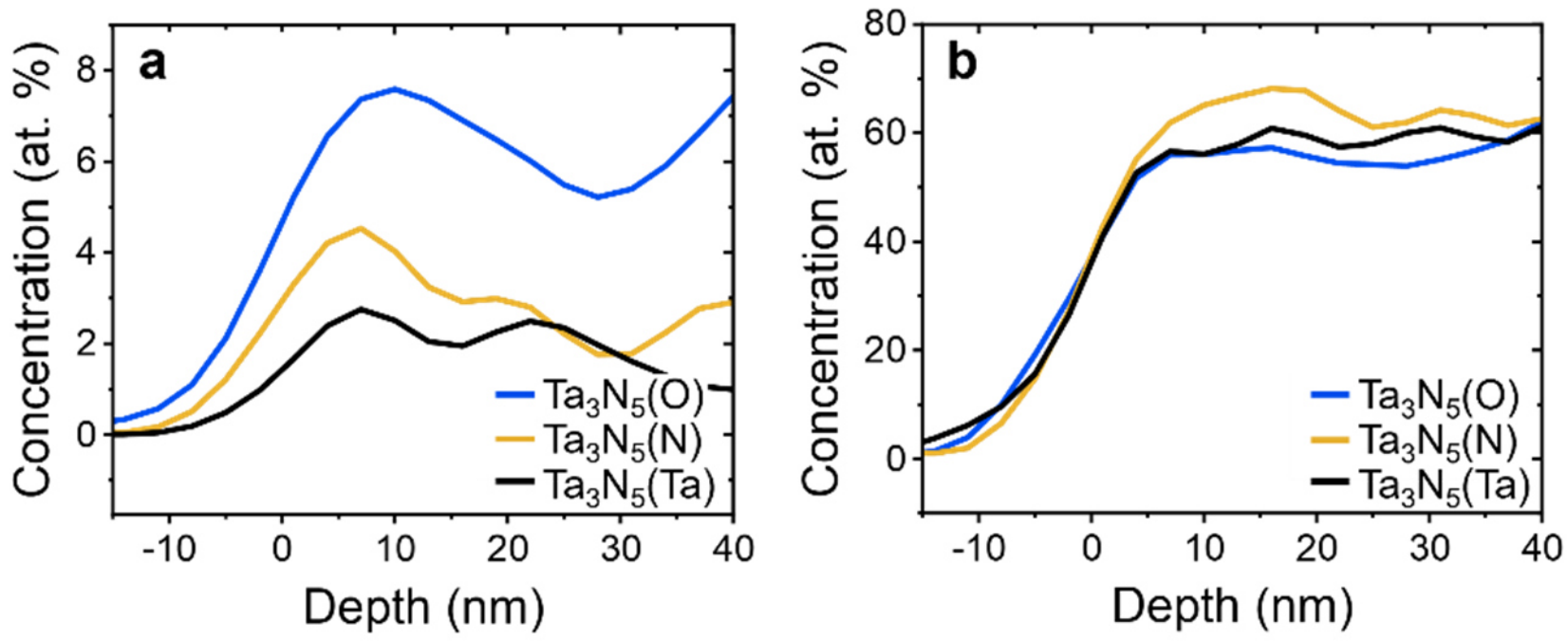

Supplementary Fig. 2 Depth-resolved elemental composition of Ta_3N_5 thin films synthesized from different precursors. **a** Oxygen and **b** nitrogen concentration profiles of Ta_3N_5(O), Ta_3N_5(N), and Ta_3N_5(Ta) obtained by ERDA. Source data are provided as a source data file.

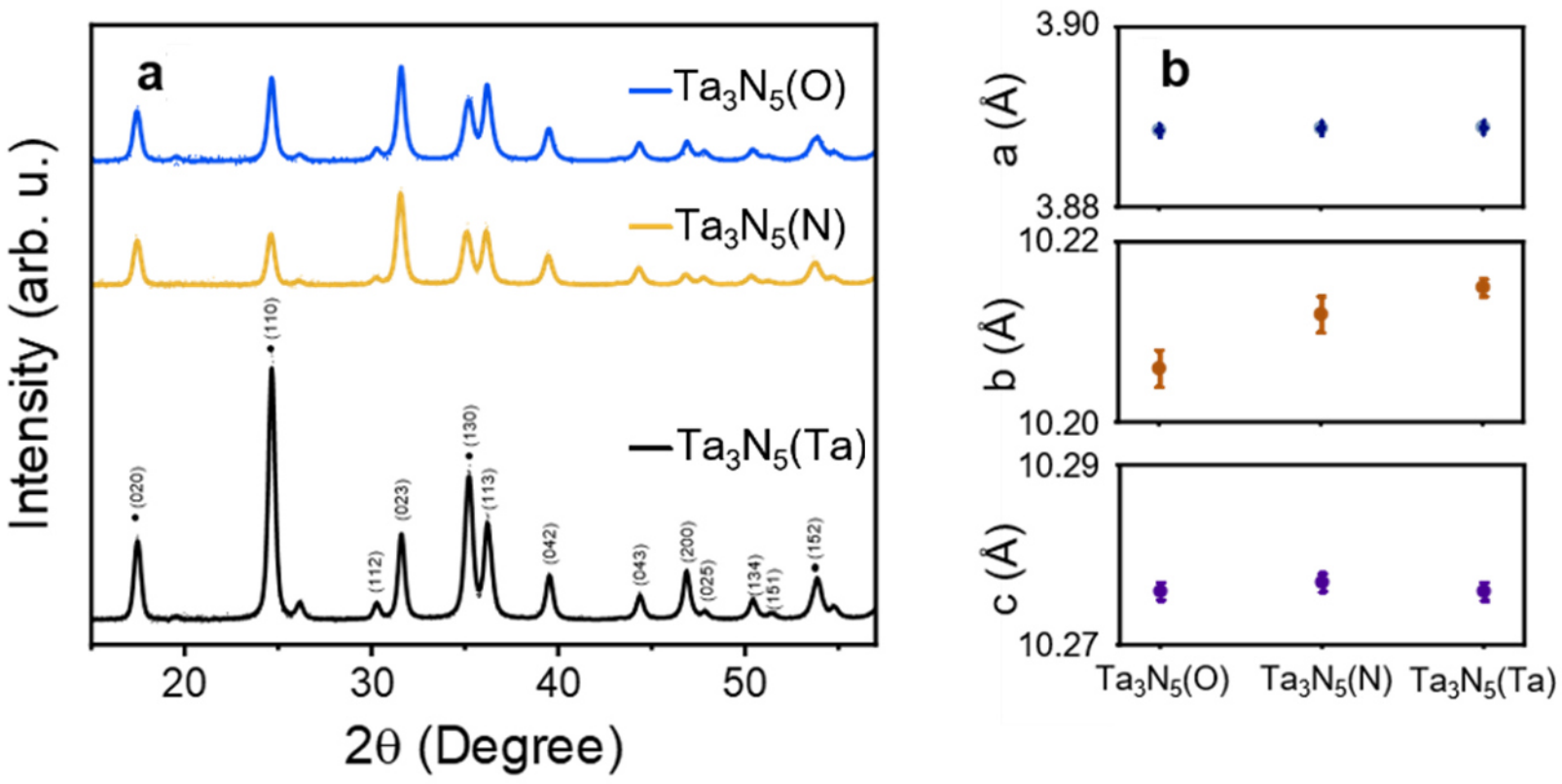

Supplementary Fig. 3 a GI-XRD patterns of Ta_3N_5 films synthesized from varying precursors. **b** Lattice parameters *a*, *b*, and *c* extracted from Rietveld refinement for Ta_3N_5(O), Ta_3N_5(N), and Ta_3N_5(Ta). Source data are provided as a source data file.

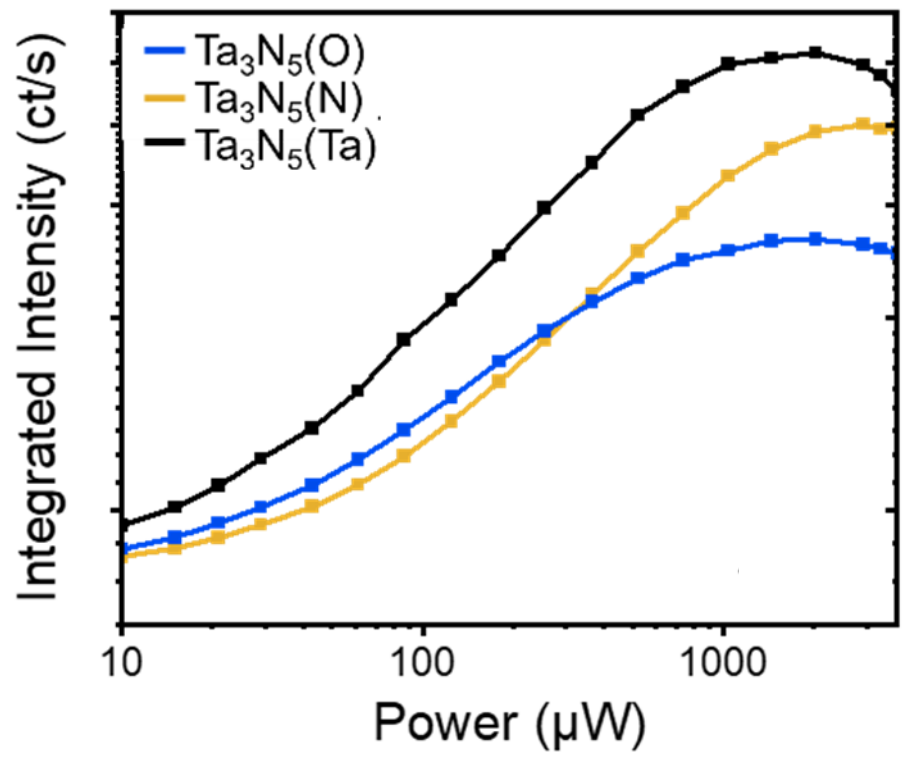

Supplementary Fig. 4 Power-dependent integrated defect photoluminescence of Ta_3N_5(O), Ta_3N_5(N), and Ta_3N_5(Ta) thin films measured at 10 K with excitation wavelength of 403 nm. Source data are provided as a source data file.

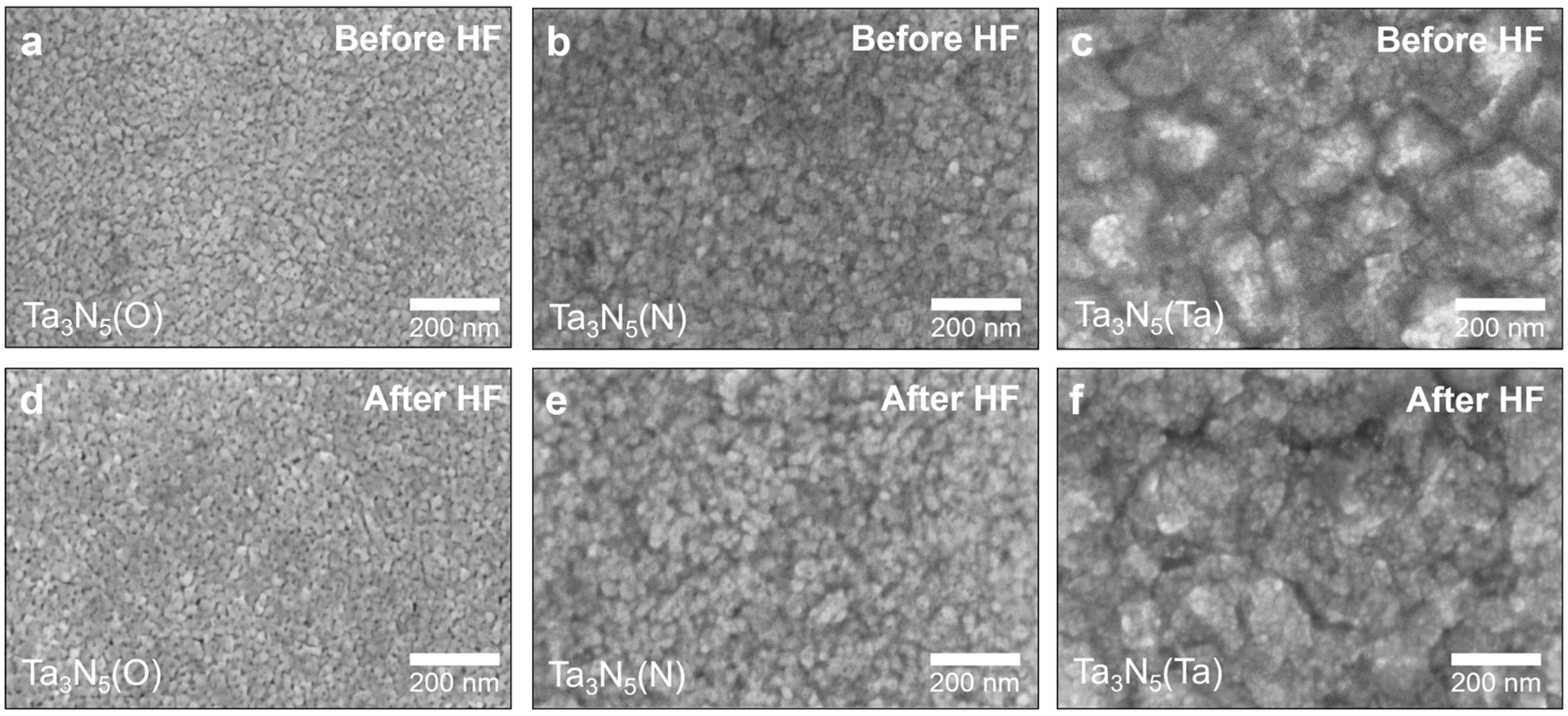

Supplementary Fig. 5 Top-view SEM topography images for Ta_3N_5(O), Ta_3N_5(N), and Ta_3N_5(Ta) thin films **a-c** before and **d-f** after HF-treatment.

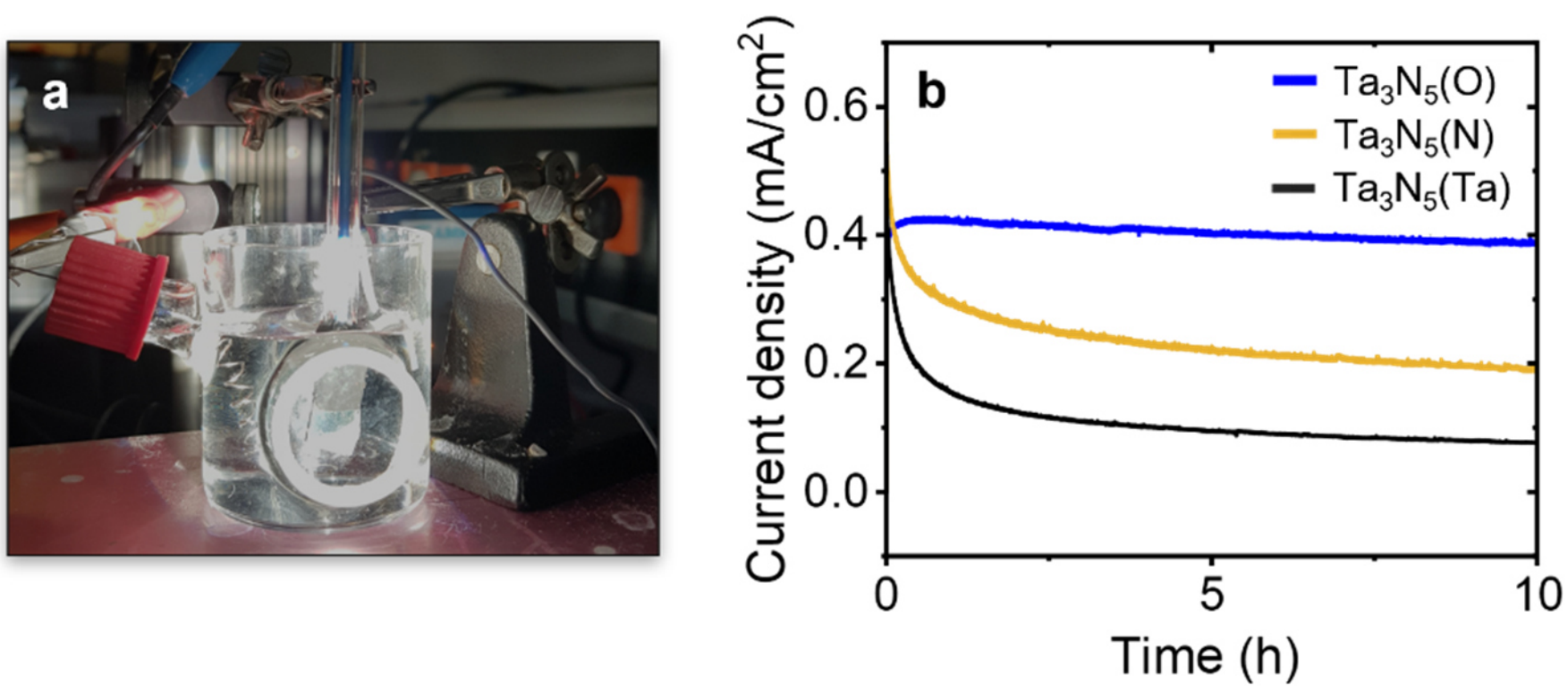

Supplementary Fig. 6 a Photo of the photoelectrochemical setup. **b** Long-term chronoamperometry tests of Ta_3N_5(O), Ta_3N_5(N), and Ta_3N_5(Ta) thin films after HF treatment, measured at 1.23 V_{RHE} under illumination (AM 1.5G) in 1 M KP_i (pH 12.3) with 0.1 M $K_4Fe(CN)_6$ as hole scavenger. Source data for b are provided as a source data file.

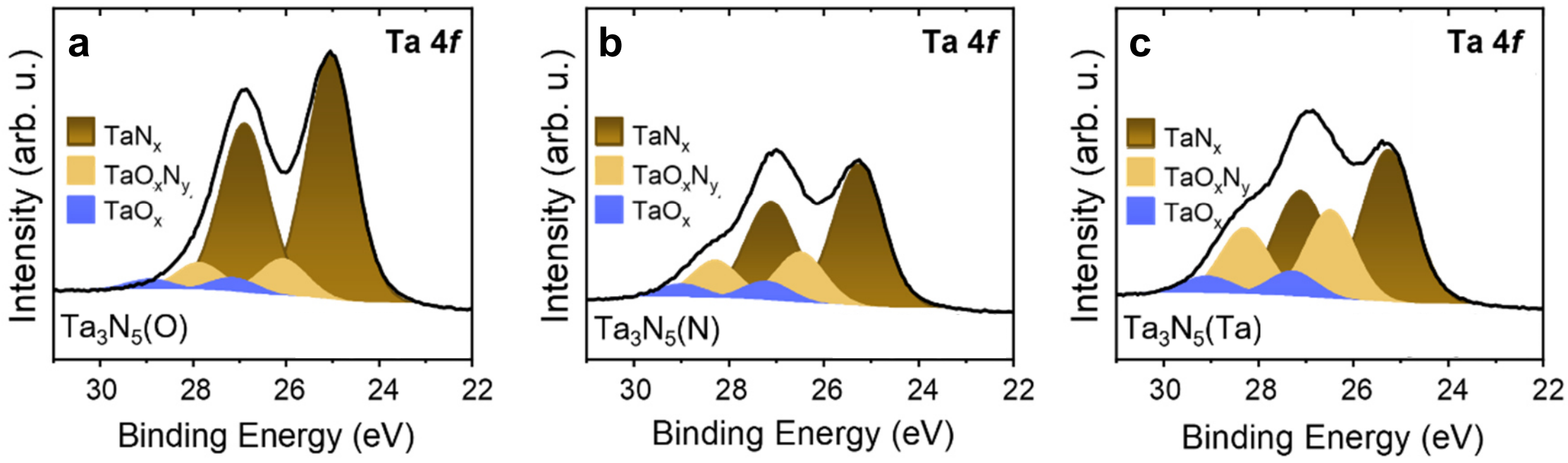

Supplementary Fig. 7 High-resolution Ta 4*f* core level spectra of HF-treated **a** Ta_3N_5(O), **b** Ta_3N_5(N), and **c** Ta_3N_5(Ta) showing peaks corresponding to TaN_x, TaO_xN_y, and TaO_x. The 10 h stability test was conducted in 1 M KP_i with 0.1 M $K_4Fe(CN)_6$ as a hole scavenger at 1.23 V_{RHE} under illumination. Source data are provided as a source data file.

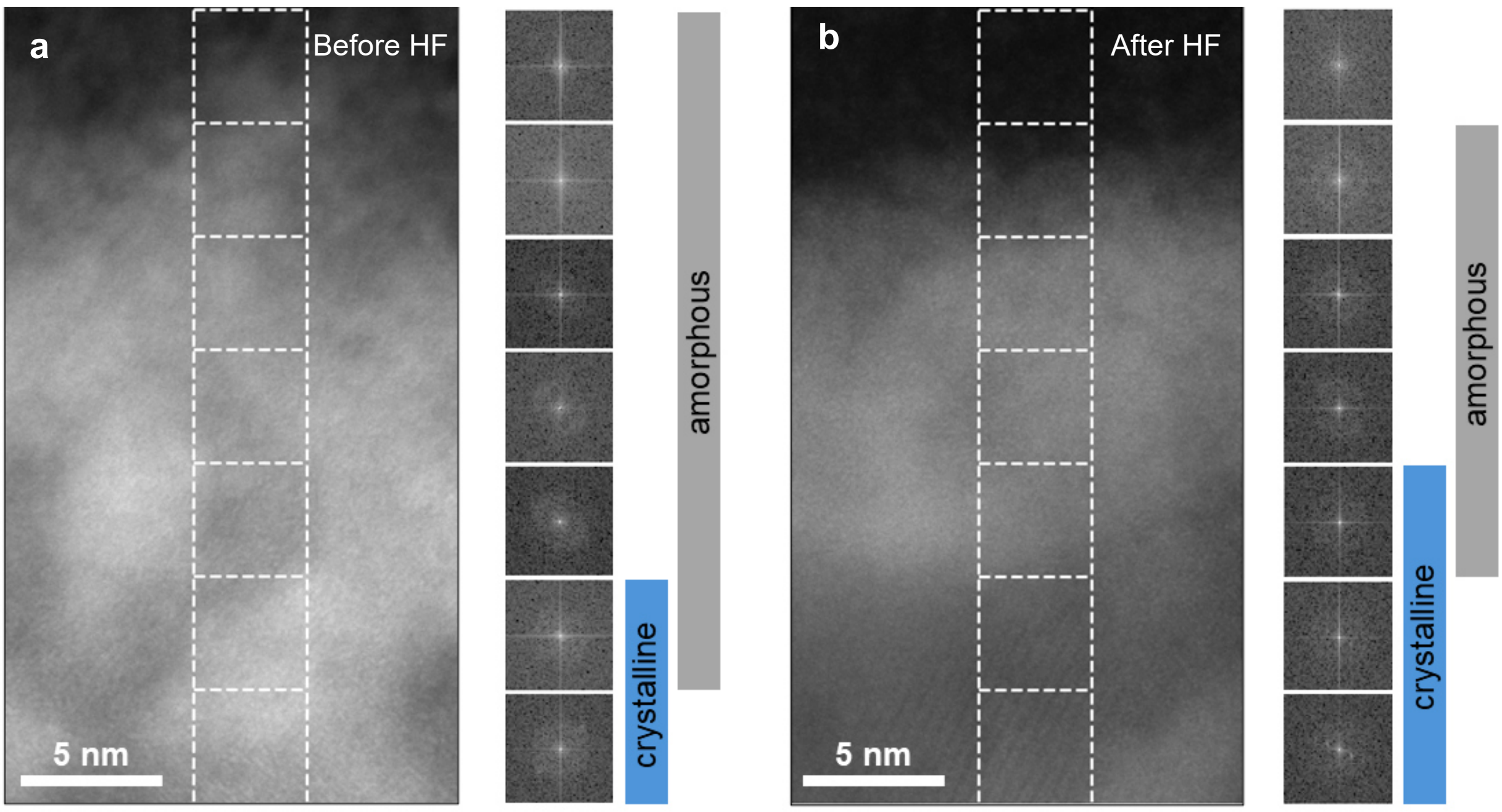

Supplementary Fig. 8 Cross-sectional HAADF STEM images for Ta_3N_5(O) films **a** before and **b** after HF-treatment.

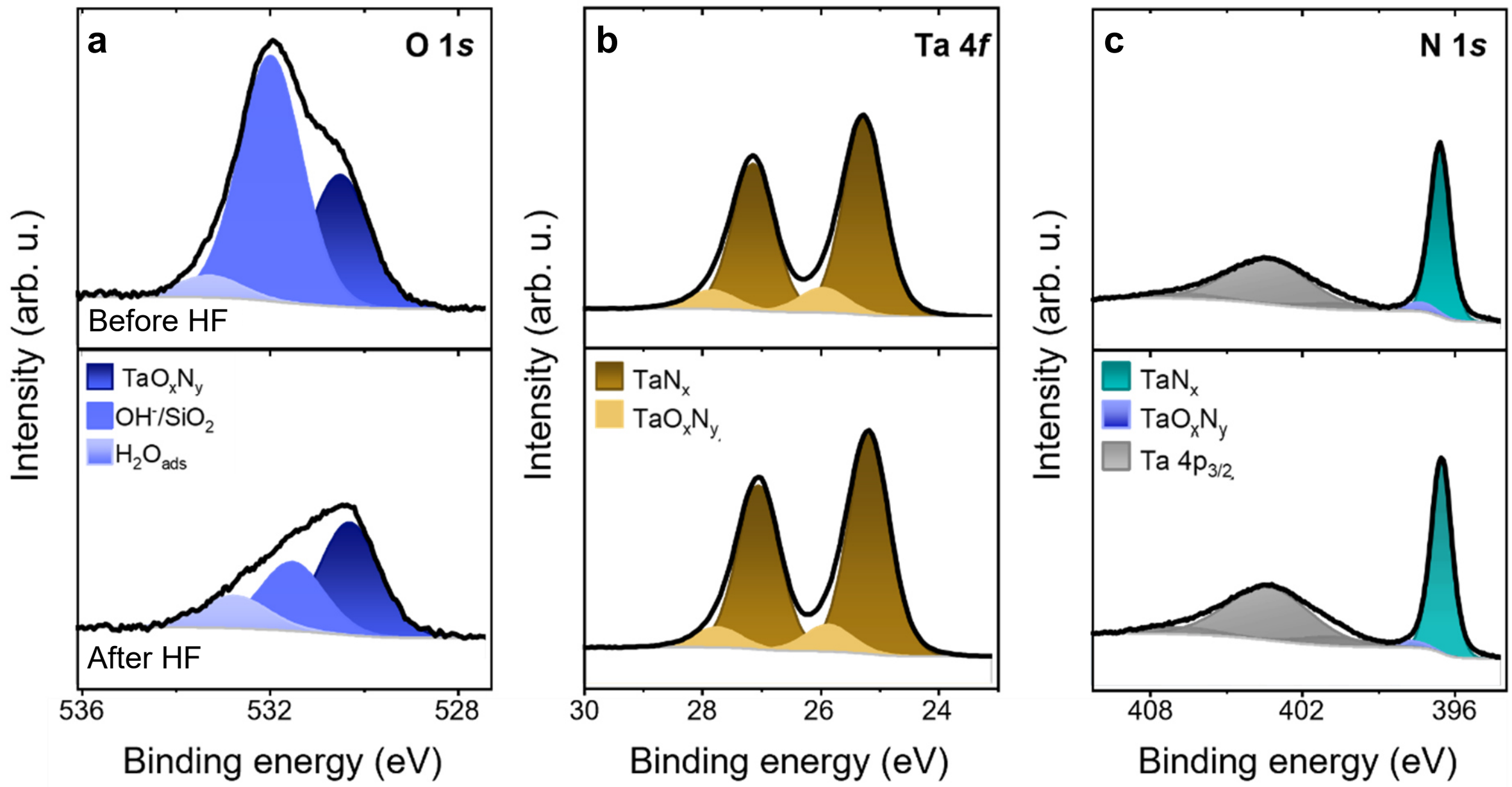

Supplementary Fig. 9 XPS spectra of Ta_3N_5(O) thin films before (top) and after (bottom) HF treatment. **a** O 1*s* spectra deconvoluted into components attributed to metal oxide/oxynitride, hydroxide/silicon oxide, and adsorbed water. **b** Ta 4*f* spectra showing peaks corresponding to TaN_x and TaO_xN_y. **c** N 1*s* spectra fitted with components for TaN_x and TaO_xN_y. Additionally, the Ta 4$p_{3/2}$ is shown in grey. Source data are provided as a source data file.

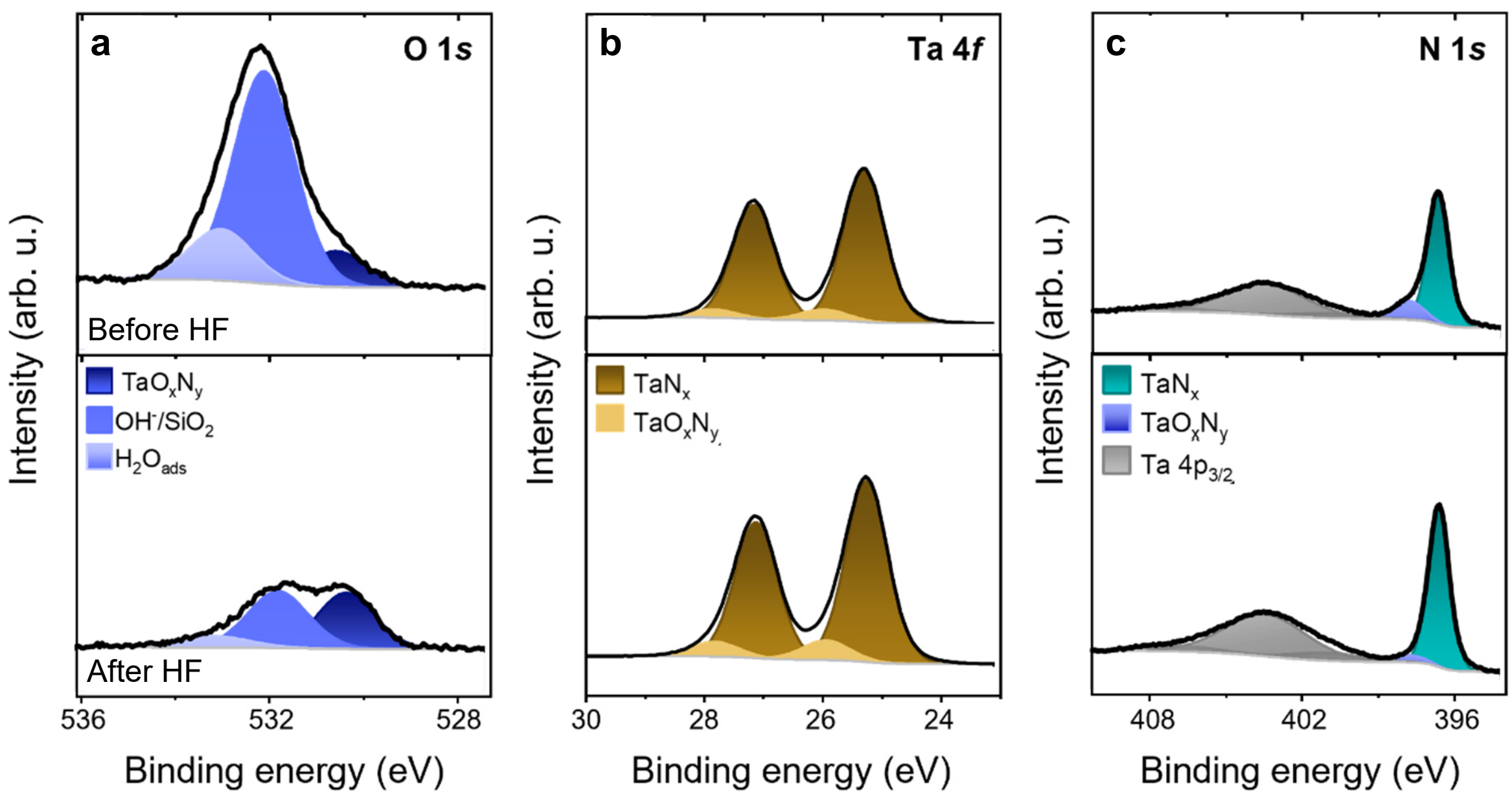

Supplementary Fig. 10 XPS spectra of Ta_3N_5(N) thin films before (top) and after (bottom) HF treatment. **a** O 1*s* spectra deconvoluted into components attributed to metal oxide/oxynitride, hydroxide/silicon oxide, and adsorbed water. **b** Ta 4*f* spectra showing peaks corresponding to TaN_x and TaO_xN_y. **c** N 1*s* spectra fitted with components for TaN_x and TaO_xN_y. Additionally, the Ta 4$p_{3/2}$ is shown in grey. Source data are provided as a source data file.

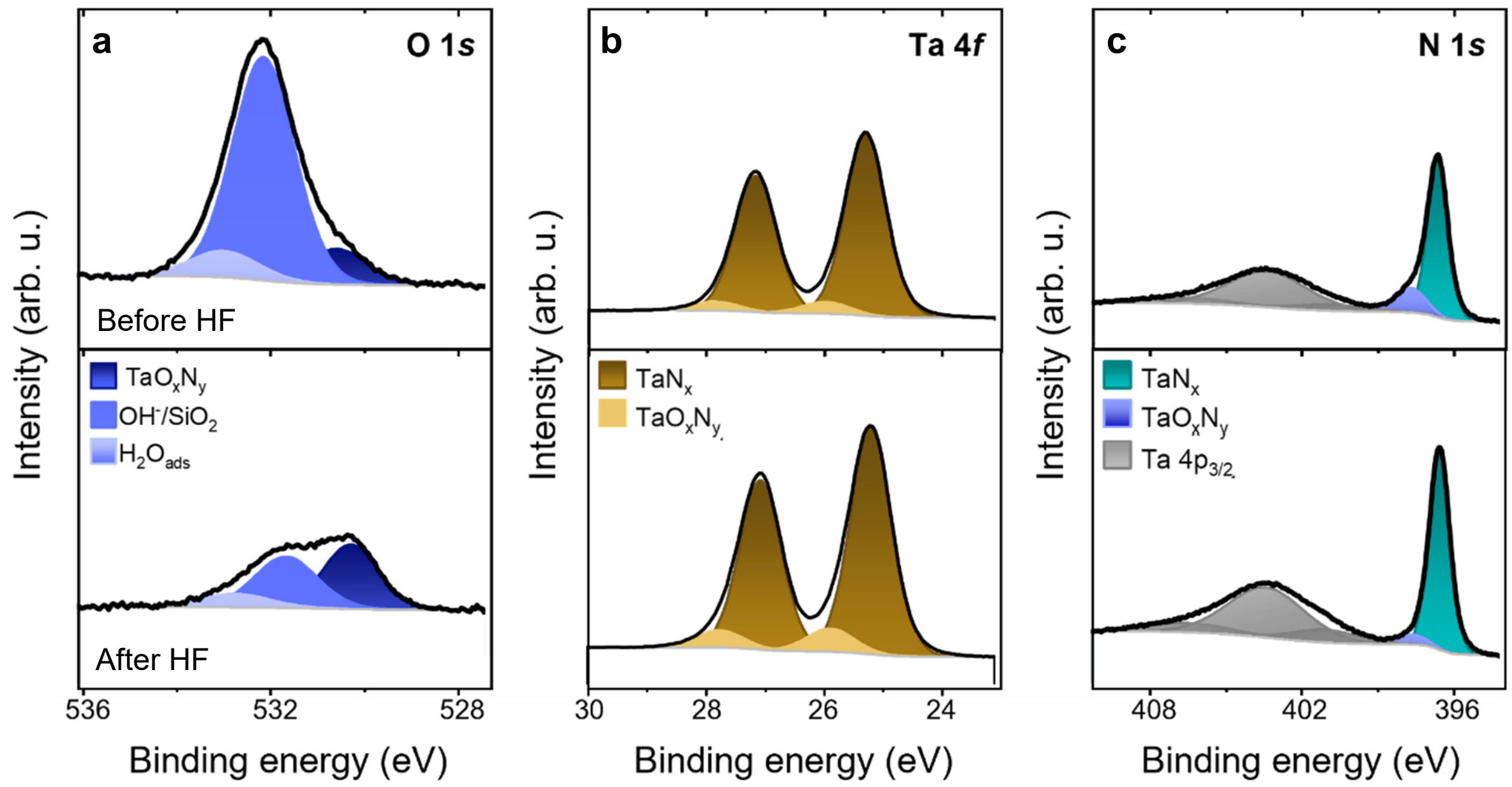

Supplementary Fig. 11 XPS spectra of Ta_3N_5(Ta) thin films before (top) and after (bottom) HF treatment. **a** O 1*s* spectra deconvoluted into components attributed to metal oxide/oxynitride, hydroxide/silicon oxide, and adsorbed water. **b** Ta 4*f* spectra showing peaks corresponding to TaN_x and TaO_xN_y. **c** N 1*s* spectra fitted with components for TaN_x and TaO_xN_y. Additionally, the Ta 4$p_{3/2}$ is shown in grey. Source data are provided as a source data file.

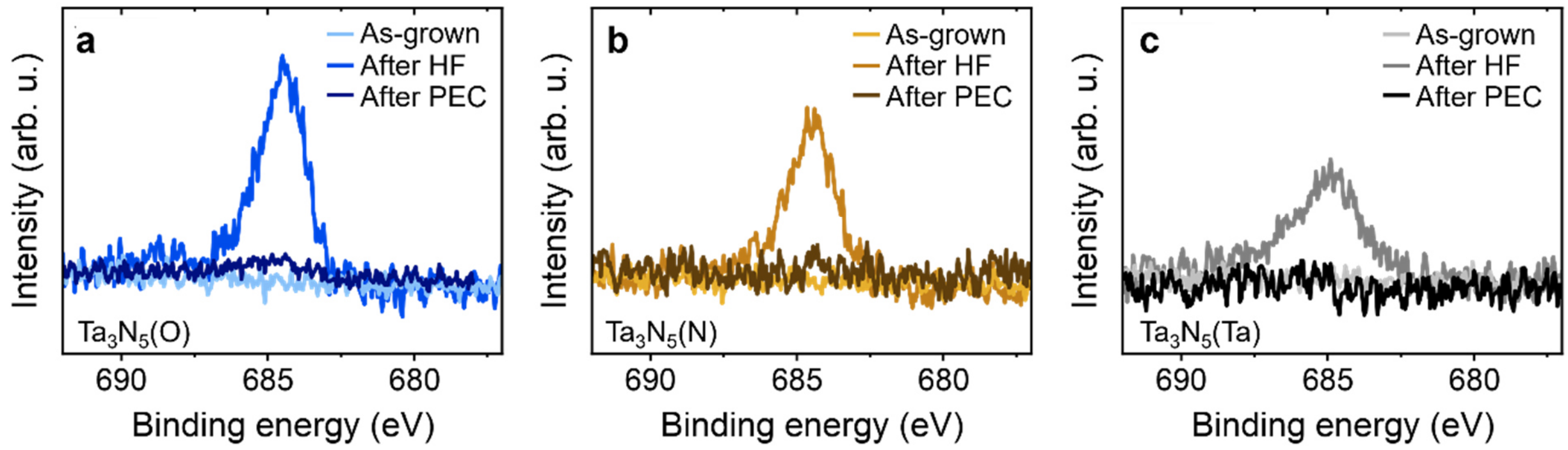

Supplementary Fig. 12 F 1*s* core level spectra of **a** Ta_3N_5(O), **b** Ta_3N_5(N), and **c** Ta_3N_5(Ta) before and after HF treatment, as well as after HF etching and PEC testing for 10 h in 1 M KP_i with 0.1 M $K_4Fe(CN)_6$ as a hole scavenger at 1.23 V_{RHE} under illumination. Source data are provided as a source data file.

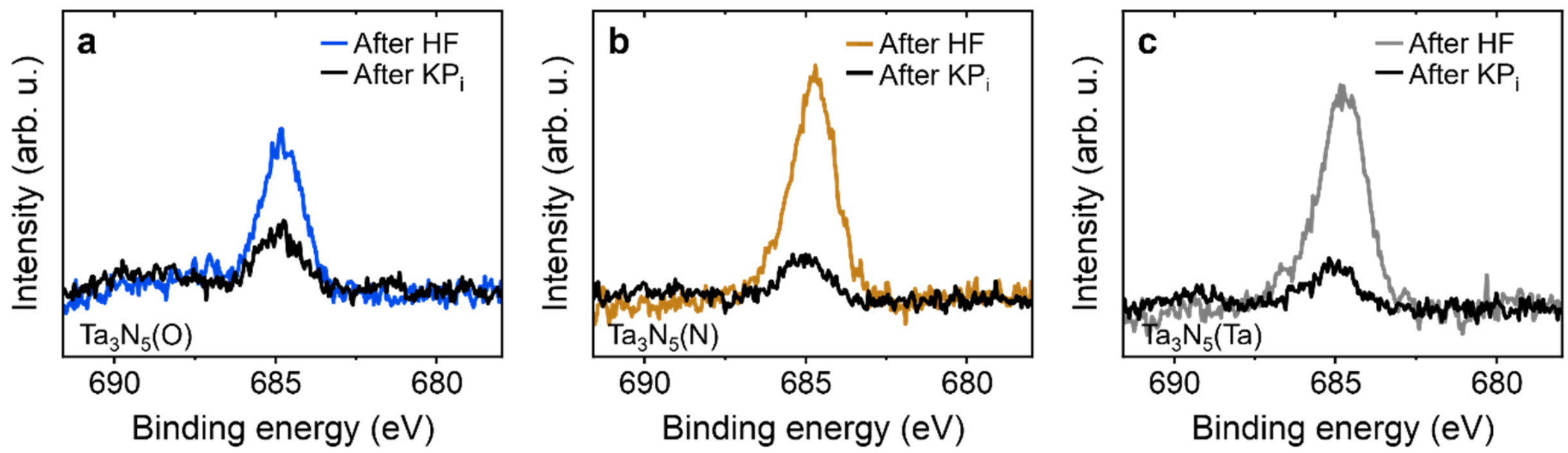

Supplementary Fig. 13 F 1*s* core level spectra of **a** Ta_3N_5(O), **b** Ta_3N_5(N), and **c** Ta_3N_5(Ta) after HF treatment, as well as immersion for 1 min in 1 M KP_i with 0.1 M $K_4Fe(CN)_6$ hole scavenger in the dark. Source data are provided as a source data file.

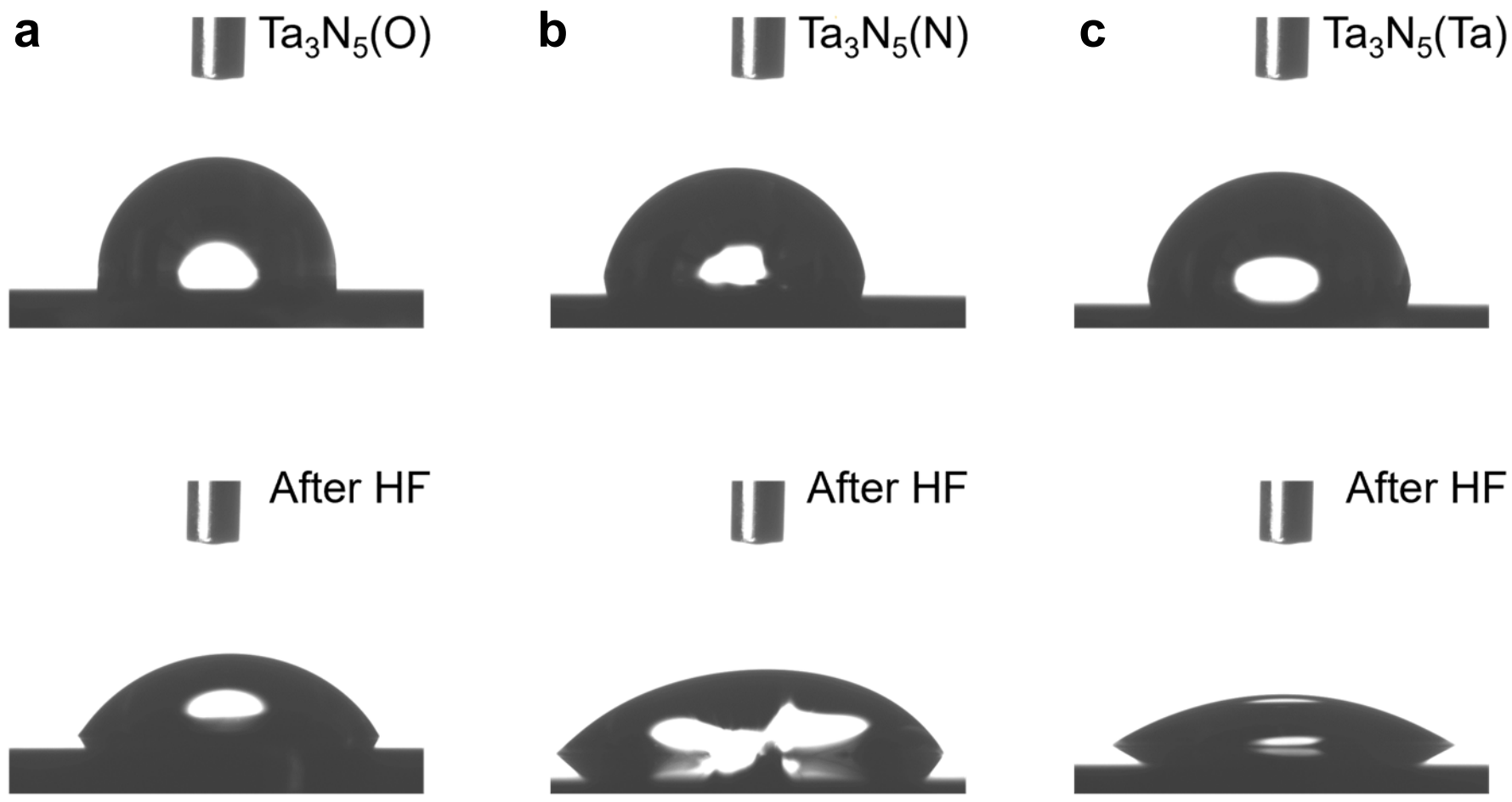

Supplementary Fig. 14 Contact angle measurements on the respective films before and after HF treatment for **a** Ta_3N_5(O), **b** Ta_3N_5(N), and **c** Ta_3N_5(Ta).

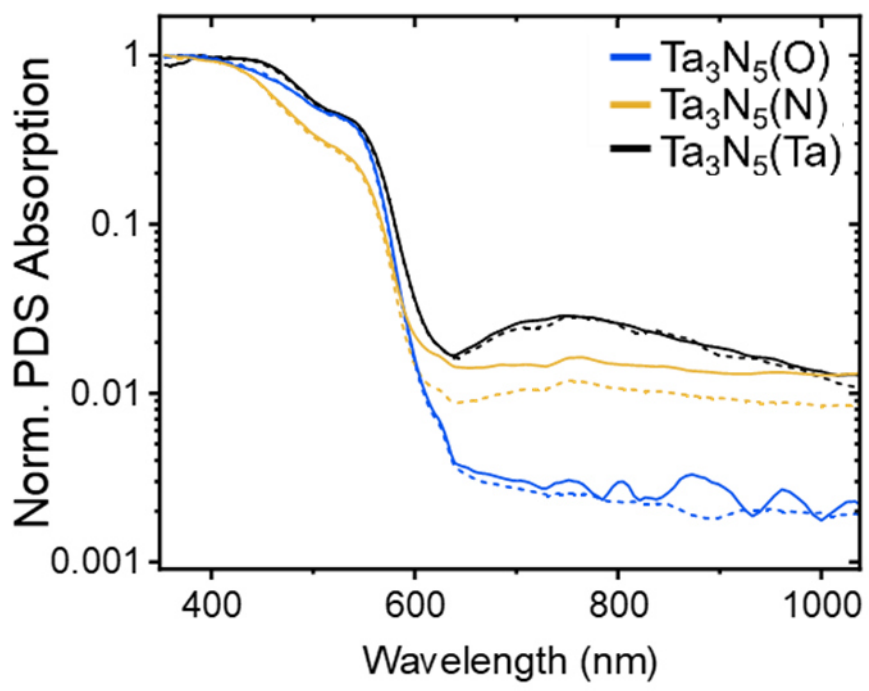

Supplementary Fig. 15 PDS of Ta_3N_5(O), Ta_3N_5(N), and Ta_3N_5(Ta) before (full line) and after (dashed line) HF treatment. Source data are provided as a source data file.

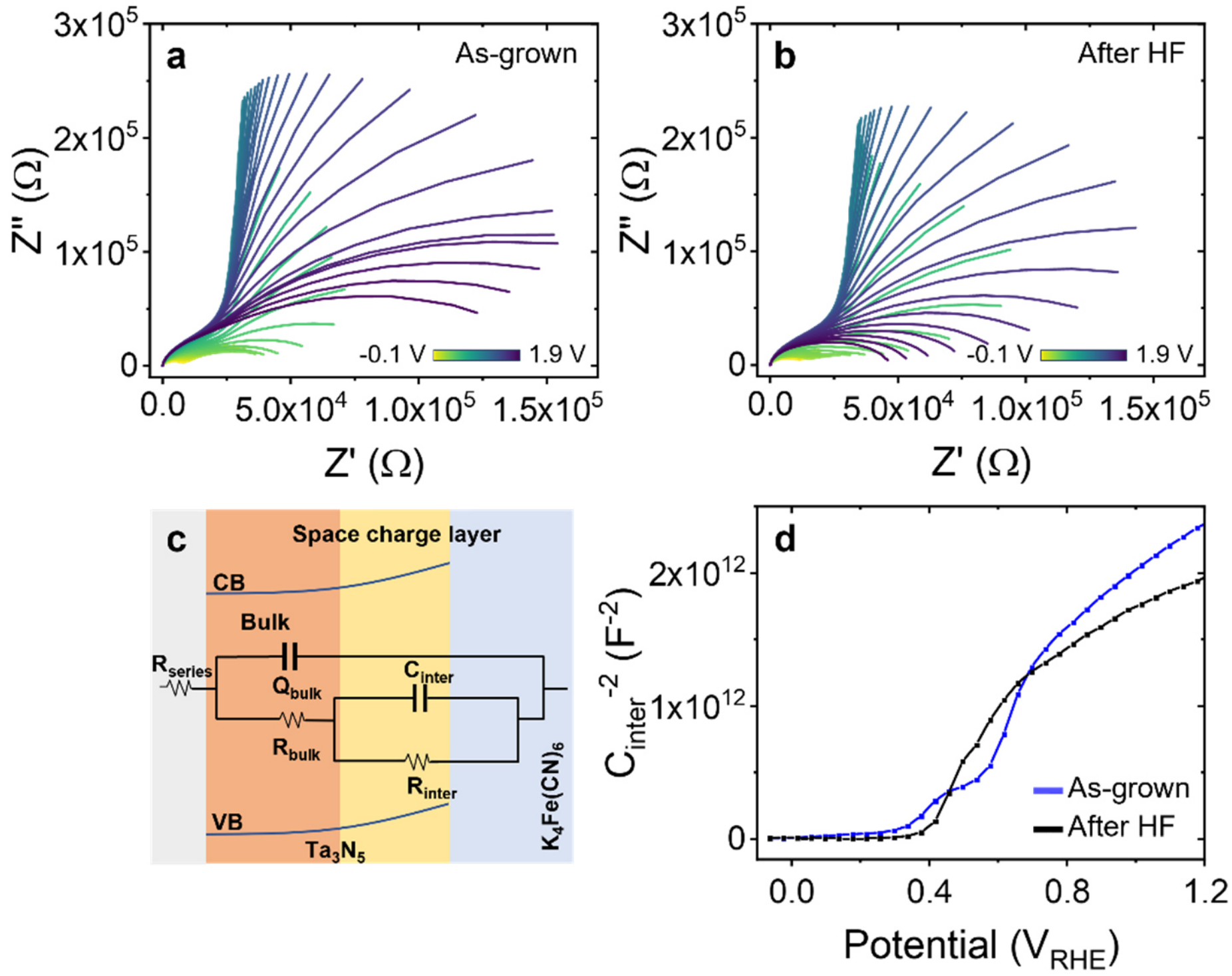

Supplementary Fig. 16 Electrochemical impedance spectroscopy of Ta_3N_5(O) was measured over a frequency range from 1 Hz to 100 kHz under dark conditions. Nyquist plots recorded over a potential range from -0.1 V_{RHE} to 1.9 V_{RHE} **a** without and **b** with HF treatment. **c** Equivalent circuit used for fitting, comprising the series resistance R_{series}, bulk (Q_{bulk}, R_{bulk}) and interface (C_{inter} and R_{inter}) contributions. **d** Mott-Schottky plots derived from capacitance values obtained from fitting the potential-dependent EIS data. In the model, R_{series} accounts for ohmic contributions from contacts and wiring. The bulk is described by a resistance R_{bulk} to charge transport through the semiconductor bulk, and a non-ideal capacitive element Q_{bulk}, is used to account for inhomogeneities. The interface is described by a capacitance C_{inter} between the electrode and the electrolyte and a charge-transfer resistance R_{inter}. Source data are provided as a source data file.

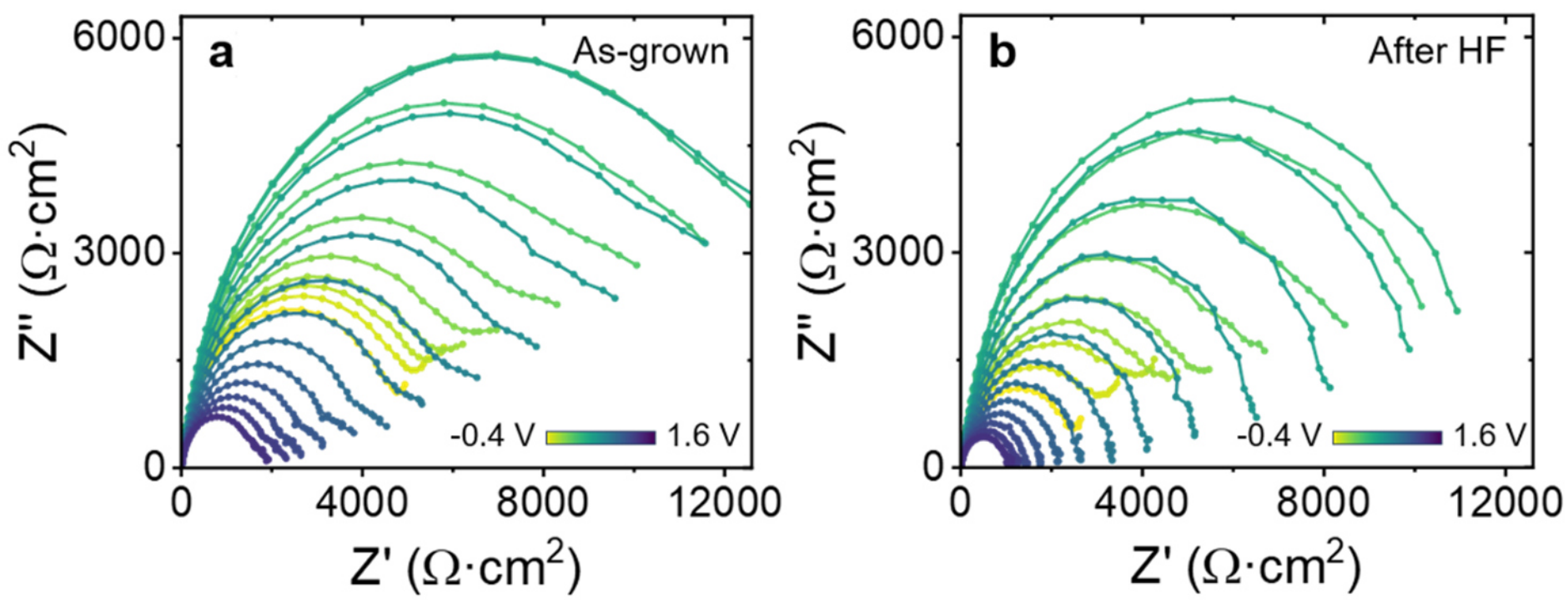

Supplementary Fig. 17 Photoelectrochemical impedance spectroscopy of Ta_3N_5(O) over a frequency range from 1 Hz to 100 kHz under illumination**.** Nyquist plots recorded over a potential range from -0.1 V_{RHE} to 1.9 V_{RHE} **a** without and **b** with HF treatment. Source data are provided as a source data file.

References

1 D. R. Baer, K. Artyushkova, C. Richard Brundle, J. E. Castle, M. H. Engelhard, K. J. Gaskell, J. T. Grant, R. T. Haasch, M. R. Linford, C. J. Powell, A. G. Shard, P. M. A. Sherwood and V. S. Smentkowski, *J. Vac. Sci. & Techno. A: Vac. Surf Films*, 2019, **37**, 031401.

2 D. I. Patel, T. Roychowdhury, V. Jain, D. Shah, T. G. Avval, S. Chatterjee, S. Bahr, P. Dietrich, M. Meyer, A. Thißen and M. R. Linford, *Surf. Sci. Spectra*, 2019, **26**, 016801.

3 K. Yasuda, *Quantum Beam Sci.*, 2020, **4**, 40.

4 H. Dosch, B. W. Batterman and D. C. Wack, *Phys. Rev. Lett.*, 1986, **56**, 1144–1147.

5 A. Hexemer and P. Müller-Buschbaum, *IUCrJ*, 2015, **2**, 106–125.

6 B. H. Frazer, B. Gilbert, B. R. Sonderegger and G. De Stasio, *Surf. Sci.*, 2003, **537**, 161–167.

7 D. Asakura, E. Hosono, Y. Nanba, H. Zhou, J. Okabayashi, C. Ban, P.-A. Glans, J. Guo, T. Mizokawa, G. Chen, A. J. Achkar, D. G. Hawthron, T. Z. Regier and H. Wadati, *AIP Advances*, 2016, **6**, 035105.

8 Z. Wei, M. Dubajic, C. Chosy, S. Kahmann and S. D. Stranks, *Nat. Rev. Methods Primers*, 2025, **5**, 37.
