# Supplementary material for: Disentangling surface and bulk properties of Ta3N5 photoanodes
Source: Nat Commun. 2026 Aug 11;17:7773. doi: 10.1038/s41467-026-76117-y (PMC13463003; doi:10.1038/s41467-026-76117-y)
Supplement: Supplementary file 2 [file 41467_2026_76117_MOESM2_ESM.pdf]

nature portfolio

Peer Review File

Disentangling surface and bulk properties of Ta_3N_5 photoanodes

Corresponding Author: Professor Johanna Eichhorn

This file contains all reviewer reports in order by version, followed by all author rebuttals in order by version.

Version 0:

Reviewer comments:

Reviewer #1

(Remarks to the Author)
In this manuscript, the authors employ depth-sensitive characterizations to disentangle surface and bulk properties of Ta3N5 thin films synthesized from different precursors. The results reveal that surfaces exhibit oxygen enrichment, increased structural disorder, and elevated deep-level defect densities relative to the bulk. After removing the surface layer by a brief hydrofluoric acid treatment, the photoelectrochemical performance and stability for ferrocyanide oxidation are markedly enhanced. Although the important role of interfacial and defect engineering in enabling efficient charge transportation and transfer is highlighted, there is no exciting discovery or large enhancement on Ta3N5 photoanodes relative to the literature reports. Therefore, I recommend the publication of the manuscript in a more special journal rather than NC. The detailed comments are listed as follows:
1. There is no morphology information of photoanodes. The top and cross-sectional SEM images of photoanodes from different precursors should be provided in the main text or SI file. Besides defects, the thickness and morphology of photoanode films also play critical roles in determining the final PEC performance.
2. As is well known that Ta3N5 is easily oxidized even under ambient conditions, the existence of oxide surface layer has been well recognized in the literature, and the remove of the surface layer by using a hydrofluoric acid contained solution has been widely adapted to improve the photoanode performance. Therefore, the discovery of the work is nothing new.
3. The PEC performance and stability are enhanced for ferrocyanide oxidation rather than water oxidation, what is the potential application of PEC ferrocyanide oxidation? Moreover, the obtained photocurrent densities are still much lower than the literature reported values, and only around 10 min stability is tested, which is too short to assess the stability of photoanodes.
4. The contents of both N and O elements are much lower at near surface region (0-10nm) relative to the bulk for all Ta3N5 photoanodes as shown in Figure S1. If surfaces exhibit oxygen enrichment, oxygen contents should be higher at near surface region. What is the reason?
5. The authors observed improved performance, particularly in terms of stability, after treating Ta3N5 with hydrofluoric acid. Since this modification method inevitably introduces fluoride ions into the surface layer of materials, detailed characterizations are necessary to determine the possible influence of fluoride ions on the performance changes.
6. Although this manuscript focused on a detailed investigation of surface and bulk crystal structures, chemical composition, and types and concentrations of defects using multiple techniques, the authors need to characterize the studied Ta3N5 using TEM, SEM, and electrochemical impedance spectroscopy (EIS) to rule out contributions from other potential factors (e.g., morphology, particle size, charge transport efficiency) to the overall performance.
7. In Figure 2a and c, there is no sample information and the colors of curves are so similar that it is not easy to identify them immediately.

Reviewer #2

(Remarks to the Author)
The manuscript presents a detailed investigation into the differentiation of surface and bulk defect states in Ta_3N_5 photoanodes using a suite of depth-sensitive structural, chemical, and optoelectronic characterization methods. The study is well structured and supported by substantial experimental data; however, several aspects of the interpretation, validation of PEC performance, quantitative analysis, and mechanistic justification require further strengthening before the manuscript can meet the publication standard of Nature Communications.

1. The authors state that HF-treated samples exhibit improved stability, but no long-term durability studies are shown. It is recommended to extend the stability test to at least 10 hours.
2. No chemical analysis of post-stability samples is provided to confirm retention of nitrogen and prevention of further oxidation. Since Ta-based nitrides are known to undergo photocorrosion, the configuration after stability testing should be analyzed with XPS or Raman.
3. The central claim is that surface disorder and deep-level traps dominate PEC behavior, but there is no quantitative defect density extraction via Mott–Schottky slopes or Deep Level Transient Spectroscopy (DLTS). The argument remains descriptive rather than mechanistic. The authors should quantify defect densities to strengthen the cause–effect link.
4. The authors attribute PEC improvements to crystallization and oxygen removal. The question to be answered is does HF simply etch away amorphous oxides, or does it also introduce fluorine species on the surface?
5. The manuscript concludes that the surface defects suppress charfe transfer, yet there is no electrochemical impedance spectroscopy (EIS). Such data would be essentail to substantiate statements about surface-meadiated recombination and transport limitations.

Reviewer #3

(Remarks to the Author)
This work reports the preparation of orthorhombic Ta3N5 using three different precursors. Based on prior studies, the stability of Ta3N5 is known to depend strongly on the choice of precursor. The authors employ GIWAXS, ERDA, and depth-dependent photoluminescence (PL) to reveal pronounced differences in defect types and concentrations between the surface and bulk regions, which are attributed to compositional variations. Ta3N5 derived from TaOx exhibits enhanced photoelectrode kinetic stability, which the authors ascribe to passivation by a disordered surface layer. They further propose that deep-level defects deteriorate kinetic stability by hindering interfacial charge injection and promoting trapping-mediated self-oxidation. In addition, HF etching is applied to all samples, resulting in hydroxyl-terminated surfaces. This treatment improves surface hydrophilicity, induces surface passivation, and consequently enhances both catalytic stability and photoelectrochemical performance. Despite these merits, several issues require clarification or further evidence:
1.Reference 4 does not report the presence of a disordered surface layer in as-synthesized, fresh Ta3N5 samples. The authors should therefore clarify the origin of the disordered surface layer observed in Ta3N5(O). Additional structural characterization of this layer—such as cross-sectional TEM—would be highly valuable.
2.GIWAXS results clearly indicate an amorphous surface region within approximately the top ~3 nm of Ta3N5. The authors attribute this amorphization to increased oxygen content and enhanced surface disorder. However, ERDA measurements show comparable O and N concentrations among all three samples down to a depth of ~8 nm. This apparent inconsistency should be addressed, and the authors should clarify whether the amorphous surface layer arises from subtle compositional differences, local bonding environments, or other structural factors.
3.The criteria used to define the boundary between the “surface” and “bulk” regions should be explicitly stated. What depth or physical parameters were used to distinguish these two regimes in the analysis?
4.It remains unclear whether the disordered surface layer in Ta3N5(O) exhibits electronic and chemical properties similar to those of the well-crystallized Ta3N5(Ta), given their markedly different defect concentrations. The depth range of the ~10 nm amorphous surface layer in Ta3N5(O) should be clearly indicated and discussed.
5.Are both surface-induced shallow oxygen vacancies and deep-level defects confined to the near-surface region? Since HF etching reduces the surface-layer thickness and removes the disordered surface layer in Ta3N5(O), all three samples should, in principle, exhibit more bulk-like characteristics after treatment. Why, then, does Ta3N5(O) continue to outperform the other two samples?
6.HF etching leads to hydroxyl termination of the surface. Should this hydroxylation be considered as introducing a new type of defect, or is it better described as surface functionalization that primarily enhances hydrophilicity? If regarded as a defect, is it shallow or deep in nature? Moreover, what is the relationship between HF-induced hydrophilicity and the precursor-dependent shallow oxygen vacancies or deep-level defects?
7.Finally, the authors should provide surface morphology and structural characterization (e.g., TEM) of all three samples after HF etching to directly confirm the removal of the disordered surface layer and to assess any morphology changes induced by the treatment.

Version 1:

Reviewer comments:

Reviewer #1

(Remarks to the Author)
After carefully reading the revised manuscript and detailed response to my and others' concerns, I now tend to support the pubulication of this study about narrow bandgap Ta3N5 that is a very important class of photocatalysts.

Reviewer #2

(Remarks to the Author)
The authors have satisfactorily addressed all of the comments and concerns raised during the review process. The revisions have significantly improved the clarity, quality, and scientific rigor of the manuscript. I appreciate the author's thorough responses and the additional analyses and discussions provided. In its current form, the manuscript is well presented and suitable for publication. I therefore recommend acceptance of the manuscript for publication.

Reviewer #3

(Remarks to the Author)
The authors have answered all questions raised by the reviewer. The quality of the manuscript has been improved.

Open Access This Peer Review File is licensed under a Creative Commons Attribution 4.0 International License, which permits use, sharing, adaptation, distribution and reproduction in any medium or format, as long as you give appropriate credit to the original author(s) and the source, provide a link to the Creative Commons license, and indicate if changes were made.
In cases where reviewers are anonymous, credit should be given to 'Anonymous Referee' and the source.
The images or other third party material in this Peer Review File are included in the article's Creative Commons license, unless indicated otherwise in a credit line to the material. If material is not included in the article's Creative Commons license and your intended use is not permitted by statutory regulation or exceeds the permitted use, you will need to obtain permission directly from the copyright holder.
To view a copy of this license, visit https://creativecommons.org/licenses/by/4.0/

Response to Reviewer Comments for Manuscript "Disentangling surface and bulk properties of Ta_3N_5 photoanodes" to Nature Communications (NCOMMS-25-78748)

We would like to thank the reviewers for their thoughtful assessment of our manuscript. We believe their constructive feedback has helped us to improve the clarity, depth, and overall presentation of our work. Below, we address each point raised by the reviewers. Reviewer comments are provided in **blue**, our responses in **black**, and any corresponding changes applied to the manuscript are noted in **red**.

Response to reviewer 1:
In this manuscript, the authors employ depth-sensitive characterizations to disentangle surface and bulk properties of Ta_3N_5 thin films synthesized from different precursors. The results reveal that surfaces exhibit oxygen enrichment, increased structural disorder, and elevated deep-level defect densities relative to the bulk. After removing the surface layer by a brief hydrofluoric acid treatment, the photoelectrochemical performance and stability for ferrocyanide oxidation are markedly enhanced. Although the important role of interfacial and defect engineering in enabling efficient charge transportation and transfer is highlighted, there is no exciting discovery or large enhancement on Ta_3N_5 photoanodes relative to the literature reports. Therefore, I recommend the publication of the manuscript in a more special journal rather than NC. The detailed comments are listed as follows:

Answer: We thank the reviewer for their constructive feedback and insightful comments. While we appreciate the reviewer's perspective, we respectfully disagree with the assessment that the present work does not represent a significant advance in the understanding of Ta_3N_5 photoelectrodes. In our study we show, for the first time, the difference in structural, chemical, and defect properties between surface and bulk of Ta_3N_5 photoanodes and how these impact the PEC characteristics. Furthermore, we reveal how different precursors impact surface and bulk properties, which has not been reported up to now. Specifically, the data reveal that (i) surface disorder and oxygen enrichment are systematically decoupled from deep-level defect densities, and (ii) the thickness and nature of the disordered near-surface region strongly depend on the precursor route, which has direct consequences for charge trapping and kinetic competition at the interface. These insights rationalize why nominally similar bulk Ta_3N_5 photoanodes exhibit dramatically different stability trends under identical photoelectrochemical conditions. Our findings highlight the need to optimize the surface properties independent of bulk properties, as demonstrated by the application of a HF post-treatment. This goes beyond typical Ta_3N_5 studies, which focus on bulk property optimization or, when interfaces are treated, assume a thin native oxide layer on the otherwise bulk semiconductor.

Importantly, we believe that these findings have implications beyond specific performance metrics, revealing more general characteristics of Ta_3N_5 that will broadly inform mechanistic interpretations and future optimization efforts. In addition, we note that the films investigated here were not specifically optimized for achieving maximum photoelectrochemical performance (see Comment 1.3 for complete details). Rather, they were designed to enable

a systematic comparison between synthesis routes. In particular, the films are relatively thin (~75 nm, compared to 800 nm for typical high efficiency films) to ensure complete nitridation of the different precursors under comparable conditions. In addition, we did not incorporate dopants or specific back contact engineering approaches since each of these would need to be individually optimized for each synthesis route, thereby precluding comparisons between the films. As such, the performance values should be viewed in the context of this comparative study, while the central conclusions regarding the different surface and bulk properties, their control through synthesis and post-synthetic etching, and their impact on PEC behavior are more fundamental and generally applicable.

Comment 1.1. There is no morphology information of photoanodes. The top and cross-sectional SEM images of photoanodes from different precursors should be provided in the main text or SI file. Besides defects, the thickness and morphology of photoanode films also play critical roles in determining the final PEC performance.

Answer: We agree with the reviewer that thickness and morphology impact the PEC performance. In the present work, all films were prepared by sputter deposition and subsequent ammonolysis, yielding continuous, polycrystalline thin films with different surface roughness, which strongly correlates with the volume change during ammonolysis.[1]

Changes: We added additional top-view SEM, AFM, and cross-sectional SEM images in Fig. S1 in the supplementary information.

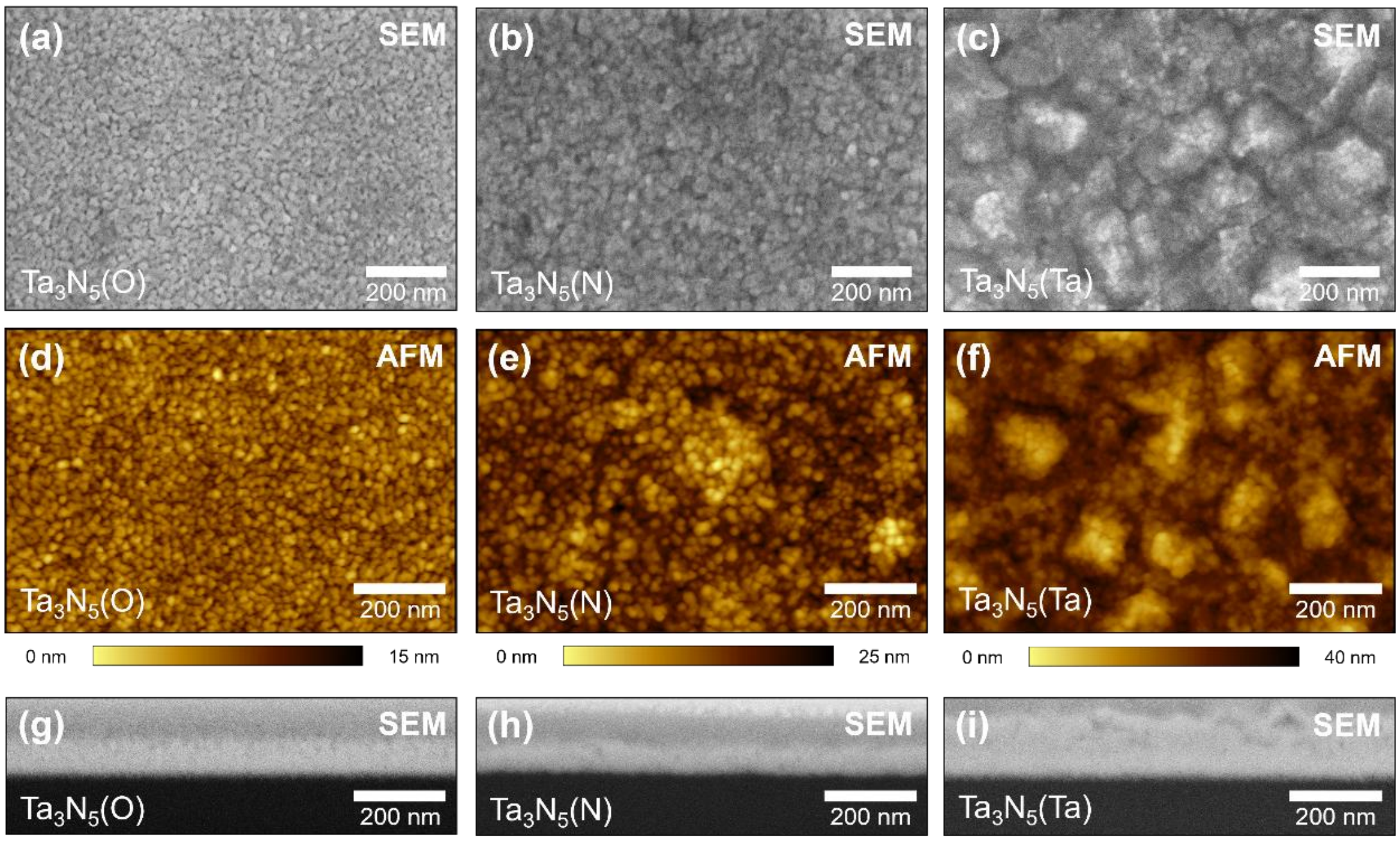

Figure S1. Top-view SEM (a-c) and AFM measurements (d-f) of Ta_3N_5(O), Ta_3N_5(N), and Ta_3N_5(Ta), respectively. (g-i) Corresponding cross-sectional SEM images.

Additionally, we revised the text in the main manuscript on page 2:

"For all precursors, homogeneous polycrystalline Ta_3N_5 photoelectrodes are formed with a thickness of ~75 ± 20 nm and increasing surface roughness from Ta_3N_5(O), Ta_3N_5(N), to Ta_3N_5(Ta) (Fig. S1)."

The corresponding experimental details were included in the Methods Section:
"For SEM imaging and focused ion beam (FIB) preparation, a Zeiss Crossbeam 550-2 KMat was used (FE-REM with GEMINI II electron optics). The SEM images were acquired at a low acceleration voltage to reduce charging effects (InLens for top view and SE2 for cross-section view; 5 kV and 100 pA in both cases). Before cutting the cross-section, the sample was protected with a thin Pt layer formed by electron beam induced deposition (1 kV, 5 nA, 1250 mC cm^{-2}, track and pixel spacing 10 nm, dwell time 0.5 µs, cycles 1, reference height 120 nm). The scanning mode was fast bidirectional, and the cycle mode was back-and-forth. The subsequent cutting steps were performed with 30 kV Ga-ions (50 pA, followed by a 10 pA polishing step). Furthermore, a dose of 200 mC cm^{-2}, a pixel and track spacing of 2.25 nm (1.25 nm for polishing), and a dwell time of 20.25 µs (31.25 µs for polishing) were applied. In both cases, we used 10 cycles and a reference depth of 1000 nm. The scanning mode was fast bidirectional and the cycle mode cross-section."

Comment 1.2: As is well known that Ta_3N_5 is easily oxidized even under ambient conditions, the existence of oxide surface layer has been well recognized in the literature, and the remove of the surface layer by using a hydrofluoric acid contained solution has been widely adapted to improve the photoanode performance. Therefore, the discovery of the work is nothing new.

Answer: We agree with the reviewer that Ta_3N_5 surface oxidation under ambient conditions has been reported. However, it is important to distinguish between the commonly assumed thin native oxide layer (typically a few nm thick) and the substantially thicker, structurally disordered near-surface layer that can form during ammonolysis of oxide precursors, which is the most widely used route to synthesize Ta_3N_5. In this study, we show that formation of the surface layer and its disorder are strongly dependent on the precursor and defect properties. By combining depth-sensitive structural, compositional, and optoelectronic probes, and correlating these to the PEC characteristics, we provide detailed and unique insights into the specific surface changes in contrast to bulk properties. By using different precursors, the surface layer can be significantly reduced, and highly crystalline surfaces are formed, in contrast to the amorphous layer that forms on Ta_3N_5(O).

Furthermore, although it is known that HF etching can improve the PEC performance of Ta_3N_5, the underlying mechanism, the role of different defect properties, and the impact on PEC stability have not been investigated in detail. In particular, prior studies generally treat HF etching as a means of removing the thin native oxide, whereas our results show that it can also modify or remove a much more extended, defect-rich, and structurally disordered surface region whose properties depend sensitively on the synthesis pathway. As such, we provide a fundamental understanding of how HF surface treatment changes the surface

properties (crystallinity, wettability, composition) after surface etching, as well as chemical composition changes after stability testing. We show that the HF treatment is more effective for more defective Ta_3N_5 with low oxygen content, such as Ta_3N_5(N) and Ta_3N_5(Ta), which has not been reported before.

Changes: Based on the reviewer's comments, we strengthened the novelty statements within the paper:

Page 1 (Abstract): "However, despite these common trends, the specific surface characteristics and their spatial extent are strongly altered depending on the chemical nature of the precursor. In detail, Ta_3N_5 derived from a TaO_x precursor forms an extended amorphous oxide-rich surface with fewer deep-level defects compared to Ta_3N_5 derived from TaN_x and Ta precursors, which exhibit thinner and more crystalline surfaces with increased densities of mid-gap defect states."

Page 5: "These results provide the first demonstration of both structural and chemical deviations between surface and bulk properties in Ta_3N_5 as a function of different defect properties and chemical compositions."

Page 9: "..., we show for the first time that kinetic stabilization of Ta_3N_5 can be achieved independent of the exact defect properties by optimizing and tuning the surface, independent of the bulk."

Page 12: "…, we identified clear differences between surface and bulk characteristics as a function of the defect properties."

Page 12: "…, enhanced PEC performance and stability are observed for ferrocyanide oxidation, even for Ta_3N_5 films with large deep-level defect concentrations."

Comment 1.3: The PEC performance and stability are enhanced for ferrocyanide oxidation rather than water oxidation, what is the potential application of PEC ferrocyanide oxidation? Moreover, the obtained photocurrent densities are still much lower than the literature reported values, and only around 10 min stability is tested, which is too short to assess the stability of photoanodes.

Answer: We thank the reviewer for the comment and for providing an expert opinion to help strengthen this work. Here, it is important to distinguish between studies that aim to optimize device-level performance for a specific system and those that seek to uncover more general and broadly applicable relationships governing material properties and interfacial processes. The present work follows the latter approach.

To gain insights into fundamental properties, it is essential to use well-defined thin films, without co-catalyst or protection layers, on simple, well-known substrates rather than highly

porous films that provide the highest photocurrent densities. Indeed, we intentionally used comparatively thin films to enable precise spectroscopic characterization and to decouple optoelectronic from catalytic effects. Specifically, this choice allowed us to investigate physical surface and bulk properties as a function of shallow and deep defect concentrations while avoiding effects of interlayer interactions with co-catalyst layers, composition gradients, or elemental interferences from substrates. To ensure homogeneous composition following ammonolysis, we intentionally chose to study thin films with a thickness significantly thinner than those known to maximize photocurrent densities and overall energy conversion efficiencies. For example, the Domen group showed that a Ta_3N_5 film thickness of approximately 800 nm is required to achieve peak photocurrent densities[2]. However, for our fundamental study, ammonolysis of such thick films would likely result in additional defects and composition gradients that inhibit physical understanding. Furthermore, the prior work used GaN back contacts and other groups typically use metal foils to ensure efficient majority carrier extraction. In contrast, we used degenerately doped Si, even though it is not necessarily ideal for electrically contacting the active layer, since a highly planar, non-nitride material was needed for precise property and composition determination. Based on the arguments above, we believe there is a strong justification for the use of thin films with non-optimal thickness and back-contacts to provide the mechanistic understanding needed for rational optimization in the future.

For water oxidation, Ta_3N_5 is used as a light absorber and is often combined with an OER catalyst. In this photoelectrode, the catalyst can have multiple functions, such as enhancing OER activity, passivating defects, and/or enhancing interfacial charge transport. Here, we focus on the fundamental surface properties of Ta_3N_5 photoanodes, which are decisive for catalyst integration, as they determine the interfacial properties and can dominate overall system stability. To reveal the fundamental properties of Ta_3N_5, we omit the integration of a catalyst on the surface and use a hole scavenger for efficient charge extraction instead. In a previous study, we already elucidated the PEC performance of the three Ta_3N_5 photoanodes for water oxidation,[1] revealing enhanced interfacial charge trapping and rapid degradation independent of their chemical composition or defect properties. Overall, we showed that the fast hole transfer to the redox couple in solution, compared to self-oxidation, can kinetically stabilize the material in ferrocyanide. In terms of potential applications of PEC ferrocyanide oxidation, we note that this redox couple has been investigated in photo-driven solar redox flow batteries.[3,4] While that is not the focus of the present work, and this redox couple is more commonly utilized as a well-understood sacrificial hole acceptor, our results could have relevance to future developments of PEC redox flow systems.

We agree with the reviewer that the 10 min chronoamperometry might be short for durability testing. Therefore, for all three HF-treated films, we have now performed continuous 10 h chronoamperometry tests in 1 M KP_i with 0.1 M $K_4Fe(CN)_6$ as hole scavenger at 1.23 V_{RHE} under illumination (AM 1.5G). The measurements clearly show that the improved stability after HF treatment persists even after 10 h under ferrocyanide oxidation conditions. Specifically, $Ta_3N_5(O)$ still shows stable operation, while a slight decay in the photocurrent density is observed for $Ta_3N_5(N)$ and $Ta_3N_5(Ta)$ after long-term operation. Compared to

before HF treatment, the degradation is clearly slower, and photocurrents can still be measured after several hours of operation.

Changes: We updated the main manuscript as follows:
On page 8: "The moderate PEC performance observed in this study reflects the deliberate use of model thin films designed to enable comparative mechanistic insights from the different precursor-based synthesis routes. In particular, the film thickness is significantly lower than the ~800 nm typically required to achieve maximum PEC efficiency, and a non-ideal Si back contact was utilized.[47] These choices were made to ensure homogeneous nitridation, enable precise spectroscopic and compositional analysis, and avoid elemental contributions from the support, even though they reduce the light harvesting efficiency and introduce additional back-contact resistances."

In addition, we included the results of 10 h chronoamperometry tests as Figure S6 in the supporting information on page 4.

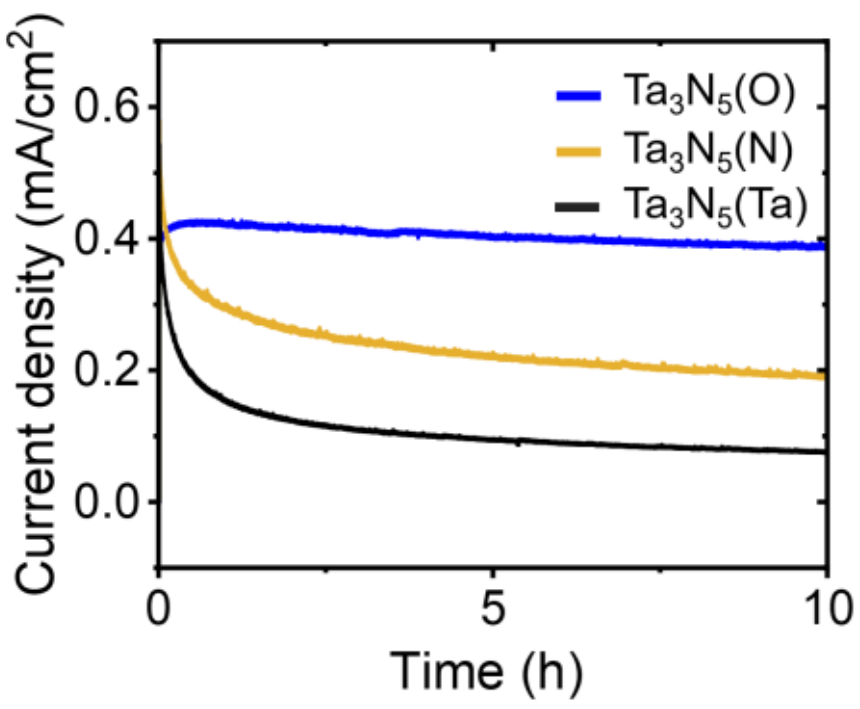

Figure S6. Long-term chronoamperometry tests of Ta_3N_5(O), Ta_3N_5(N), and Ta_3N_5(Ta) thin films after HF treatment, measured at 1.23 V_{RHE} under illumination (AM 1.5G) in 1 M KP_i (pH 12.3) with 0.1 M $K_4Fe(CN)_6$ as hole scavenger.

We included the results in the discussion on page 8:
"Even after 10 h of operation, Ta_3N_5(O) still shows stable operation, while a decay in the photocurrent density is observed for Ta_3N_5(N) and Ta_3N_5(Ta)."

Comment 1.4: The contents of both N and O elements are much lower at near surface region (0 - 10 nm) relative to the bulk for all Ta_3N_5 photoanodes as shown in Figure S1. If surfaces exhibit oxygen enrichment, oxygen contents should be higher at near surface region. What is the reason?

Answer: We thank the reviewer for pointing this out. Indeed, the data show a gradual increase in oxygen and nitrogen concentrations in the first ~10 nm, which is due to the limited depth resolution of ERDA. As a result, the physical profiles are convolved with the system resolution and physical effects such as straggling. Therefore, we only used ERDA to indicate the general trend in the distribution of oxygen and nitrogen within the film without focusing on the exact depth profile. The important information we gain from ERDA measurements is

that nitrogen is rather homogeneously distributed across the films, while higher oxygen concentrations are observed in the near-surface region than in the bulk. The gained insights are further supported by XPS measurements.

The comment motivated a much closer inspection of the ERDA data. Through this analysis, we discovered that a sample naming error led to the inclusion of the incorrect dataset for Ta_3N_5(O) and Ta_3N_5(N). In the revised version, we included the correct data and improved the analysis. While the key interpretations are not affected, the correct data are in better agreement with the insights gained from the other techniques. Specifically, we observe significantly higher oxygen content in the bulk and at the surface for Ta_3N_5(O) and Ta_3N_5(N), whereas it is more homogeneously distributed in Ta_3N_5(Ta). Overall, this inadvertent error motivated us to carefully review all other data sets presented, and we can verify that they are correct.

Changes: We updated Fig. S2 in the supporting information.

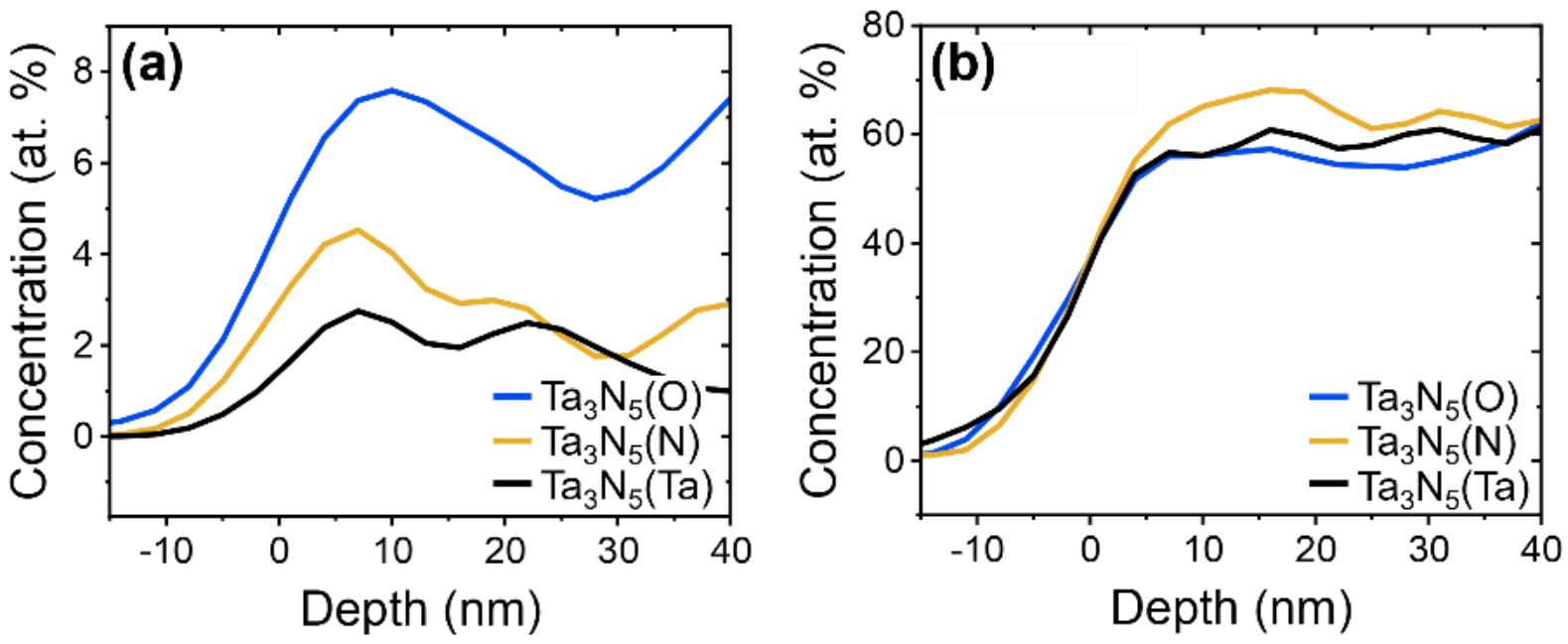

Figure S2. Depth-resolved elemental composition of Ta_3N_5 thin films synthesized from different precursors. (a) Oxygen and (b) nitrogen concentration profiles obtained by ERDA.

Furthermore, we improved the discussion on page 3:

"For ERDA measurements, it is important to note that the gradual increase in element concentrations in the first ~10 nm arises from the limited depth resolution of the detection system, broadening the depth profile. Therefore, we only use ERDA to reveal general trends in the distribution of oxygen and nitrogen near the surface and within the bulk of the films. We observe an increase of near-surface oxygen content relative to bulk, which is most pronounced for Ta_3N_5(O) with the highest oxygen content, and is reduced for Ta_3N_5(N) and Ta_3N_5(Ta), which contain lower oxygen concentrations. In contrast, the nitrogen content exhibits a more homogeneous distribution throughout the bulk. Overall, these results show a pronounced difference between surface and bulk compositions, with these variations depending sensitively on the synthesis route."

Comment 1.5: The authors observed improved performance, particularly in terms of stability, after treating Ta_3N_5 with hydrofluoric acid. Since this modification method inevitably introduces fluoride ions into the surface layer of materials, detailed characterizations are necessary to determine the possible influence of fluoride ions on the performance changes.

Answer: We agree with the reviewer that the possible impact of fluoride ions on performance should be discussed. The thin films were treated with HF (5%) for 1 min and subsequently rinsed with DI water. To identify the fluoride surface termination, we performed XPS measurements of the F 1s core level on the thin films directly after growth, after HF treatment, and after the PEC stability test. In detail, we confirmed that no fluoride ions are present at the surface of the as-grown films. As suggested by the reviewer, after HF-treatment, we do observe the adsorption of fluoride ions on the surface. However, after PEC stability tests the fluoride peak disappears again for all three thin films. Accordingly, the F 1s contribution is not present after PEC testing for 10 h in 1 M KP_i with hole scavenger, while the stability is still improved. Additionally, we tested the photoelectrode immersing for 1 min in 1 M KP_i with hole scavenger in the dark. The F 1s signal decreases strongly indicating the rapid loss of the fluoride-related contribution. Accordingly, the fluoride ions are only weakly bound to the surface and are unstable under the aqueous conditions relevant to PEC testing.

These findings strongly suggest that the surface termination with fluoride ions is not the main factor contributing to enhanced stability. Rather, we correlate the improvement to removal/thinning of the disordered near-surface region (GIWAXS), reduced O(530 eV)/Ta (XPS), and increased hydrophilicity (contact angle). Furthermore, to understand the impact on charge transfer and transport, additional EIS measurements are included, as described in Comment 1.6.

Changes: We included the F 1s core level spectra as Figure S12 and S13 in the supporting information.

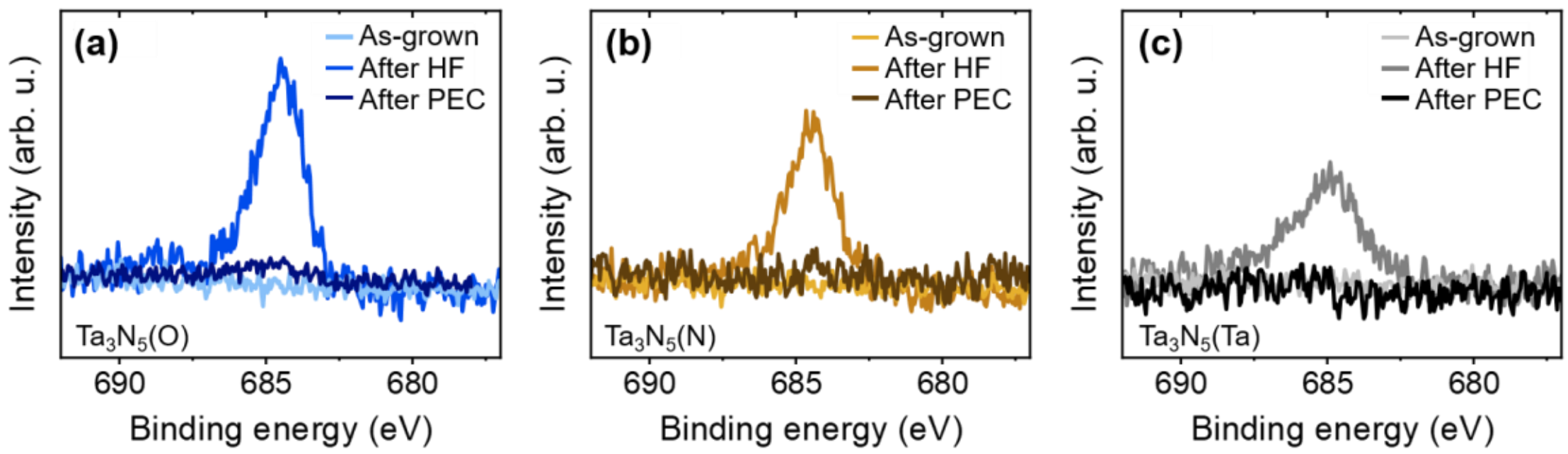

Figure S12. High-resolution F 1s core level spectra of (a) Ta_3N_5(O), (b) Ta_3N_5(N), and (c) Ta_3N_5(Ta) before and after HF treatment, as well as after HF etching and PEC testing for 10 h in 1 M KP_i with hole scavenger.

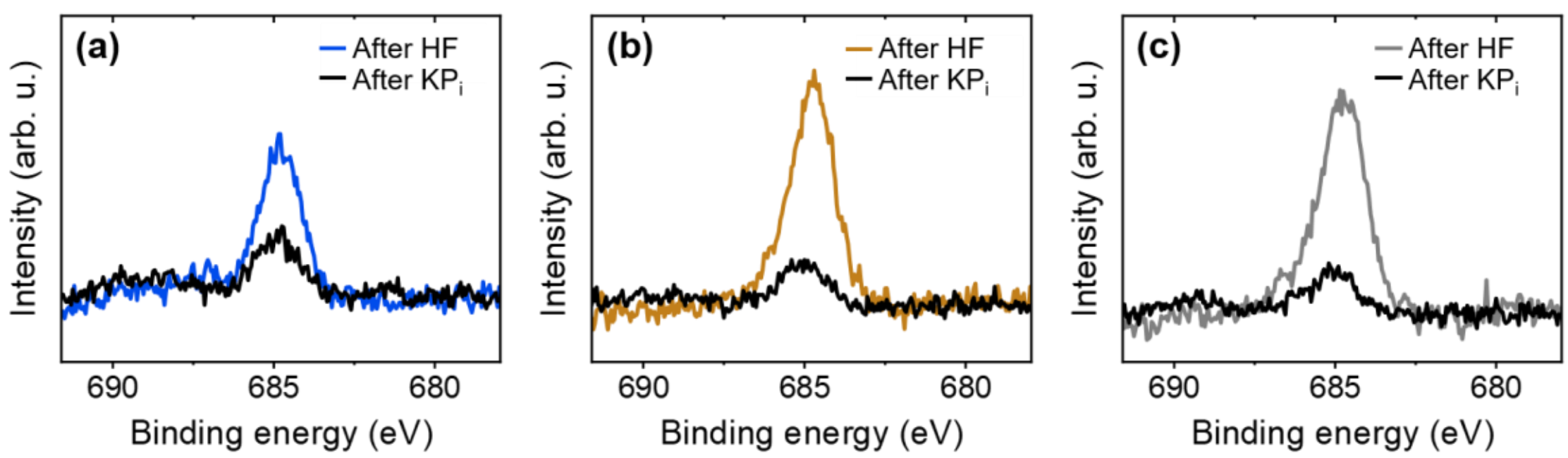

Figure S13. High-resolution F 1s core level spectra of (a) Ta_3N_5(O), (b) Ta_3N_5(N), and (c) Ta_3N_5(Ta) after HF treatment, as well as 1 min in electrolyte.

Furthermore, we improved the discussion on page 10:
"Furthermore, we analyzed the F 1s and O 1s to better understand the surface properties of the different Ta_3N_5 thin films. In the F 1s spectra (Fig. S12), we observed the presence of fluoride ions only after HF treatment for all films. In the O 1s spectra, hydroxyl groups and exposed regions of silicon terminated by sub-oxides have overlapping signals at ~532 eV. To understand the presence of hydroxyl groups on the surface, the contributions need to be deconvoluted (Table S6). The remnant photoemission intensity after subtracting the silicon oxide contribution suggests that hydroxyl groups are also present at the surface after HF treatment. Additionally, we analyzed the chemical composition after PEC testing for 10 h and immersion for 1 min in 1 M KP_i with a hole scavenger (Fig. S12 and S13), which shows the fast disappearance of the F 1s contribution on the surface, while the stability is still improved. These findings indicate that fluoride ion absorption on the surface is not the main reason for the observed stabilization."

Comment 1.6: Although this manuscript focused on a detailed investigation of surface and bulk crystal structures, chemical composition, and types and concentrations of defects using multiple techniques, the authors need to characterize the studied Ta_3N_5 using TEM, SEM, and electrochemical impedance spectroscopy (EIS) to rule out contributions from other potential factors (e.g., morphology, particle size, charge transport efficiency) to the overall performance.

Answer: We thank the reviewer for the suggestions. In comment 1.1, we included additional AFM, as well as top-view and cross-sectional SEM images to better characterize the surface morphology. For all precursors, homogeneous polycrystalline Ta_3N_5 photoelectrodes are formed with increasing surface roughness from Ta_3N_5(O) to Ta_3N_5(N) to Ta_3N_5(Ta), which does not result in a significant enhancement in PEC performance. In addition, to address the reviewer's comment, we performed additional cross-sectional TEM analysis to directly observe the amorphous layer on the surface. As shown below, these new measurements are in agreement with the GIWAXS results and show that the thickness of the disordered surface layer decreases from Ta_3N_5(O) to Ta_3N_5(N) to Ta_3N_5(Ta).

At the reviewer's suggestion, we have now also performed both electrochemical impedance spectroscopy (EIS) and photoelectrochemical impedance spectroscopy (PEIS) on Ta_3N_5 thin films. However, it is important to note that Ta_3N_5(N) and Ta_3N_5(Ta) are not stable under ferrocyanide conditions without HF treatment (Fig. 4), thus preventing reliable comparison of PEIS data from these films before and after etching. Therefore, to assess the general impact of the HF treatment, we now include the EIS and PEIS measurements on Ta_3N_5(O) with and without HF treatment. First, we recorded EIS over a potential range from -0.1 V_{RHE} to 1.9 V_{RHE} and a frequency range from 1 Hz to 100 kHz under dark conditions for Ta_3N_5(O) with and without HF treatment. The data were fitted with an equivalent circuit to describe Ta_3N_5(O) in contact with the electrolyte. The assignment of the circuit elements, is consistent with previous EIS interpretations for Ta_3N_5 in literature.[5–7] In detail, the electronic circuit consists of a series resistance (R_{series}), a bulk-related response (R_{bulk}, Q_{bulk}), and an

interfacial response (R_{inter}, C_{inter}). In this model, R_{series} accounts for ohmic contributions from e.g. contacts and wiring. The bulk is described by a resistance R_{bulk} to charge transport through the semiconductor bulk, and a non-ideal capacitive element, Q_{bulk}, is used to account for inhomogeneities. The interface is described by a capacitance C_{inter} between the electrode and the electrolyte and the charge-transfer resistance R_{inter}. The size of the observed semicircle varies strongly with potential, indicating a pronounced potential dependence of the components.

We used the fitted capacitances from broadband EIS to derive the Mott-Schottky plot (C^{-2} vs. V_{RHE}) for Ta_3N_5(O) with and without HF treatment. The as-grown Ta_3N_5(O) film exhibits a clear two-slope behavior, with a change of slope at more anodic applied potentials. Such behavior is consistent with Fermi-level pinning by surface states at low potentials, resulting in a reduced effective band-bending, and a transition to a more depletion-dominated regime at higher potentials.[6] After HF treatment, a single-regime behavior is observed over the investigated potential range, consistent with a reduced influence of surface states following removal of the surface layer. Overall, the results support the conclusion that HF treatment reduces surface defect contributions by removing the surface layer.

Second, PEIS measurements were performed over a potential range from -0.1 V_{RHE} to 1.9 V_{RHE} for Ta_3N_5(O) without and with HF treatment under illumination. At comparable applied potentials after HF treatment, the height and diameter of the semicircles are reduced compared to without HF treatment, indicating a lower overall resistance under operating conditions and more favorable photoelectrochemical charge-transfer characteristics.

Changes: We added additional top-view SEM, AFM, and cross-sectional SEM images in Fig. S1 in the supplementary information and TEM images in Fig. 2 of the main manuscript.

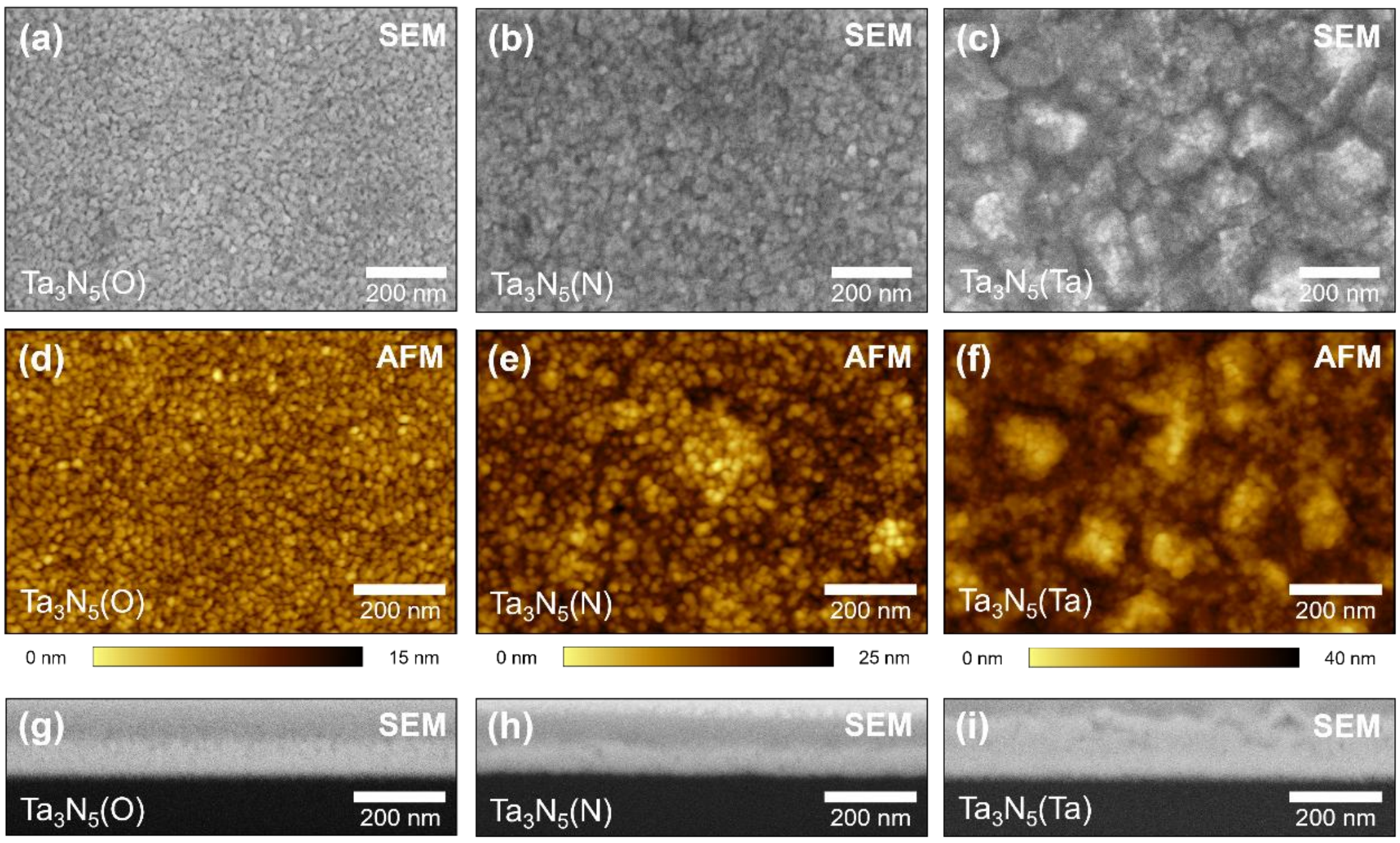

Figure S1. Top-view SEM (a-c) and AFM measurements (d-f) of Ta_3N_5(O), Ta_3N_5(N), and Ta_3N_5(Ta), respectively. (g-i) Corresponding cross-sectional SEM images.

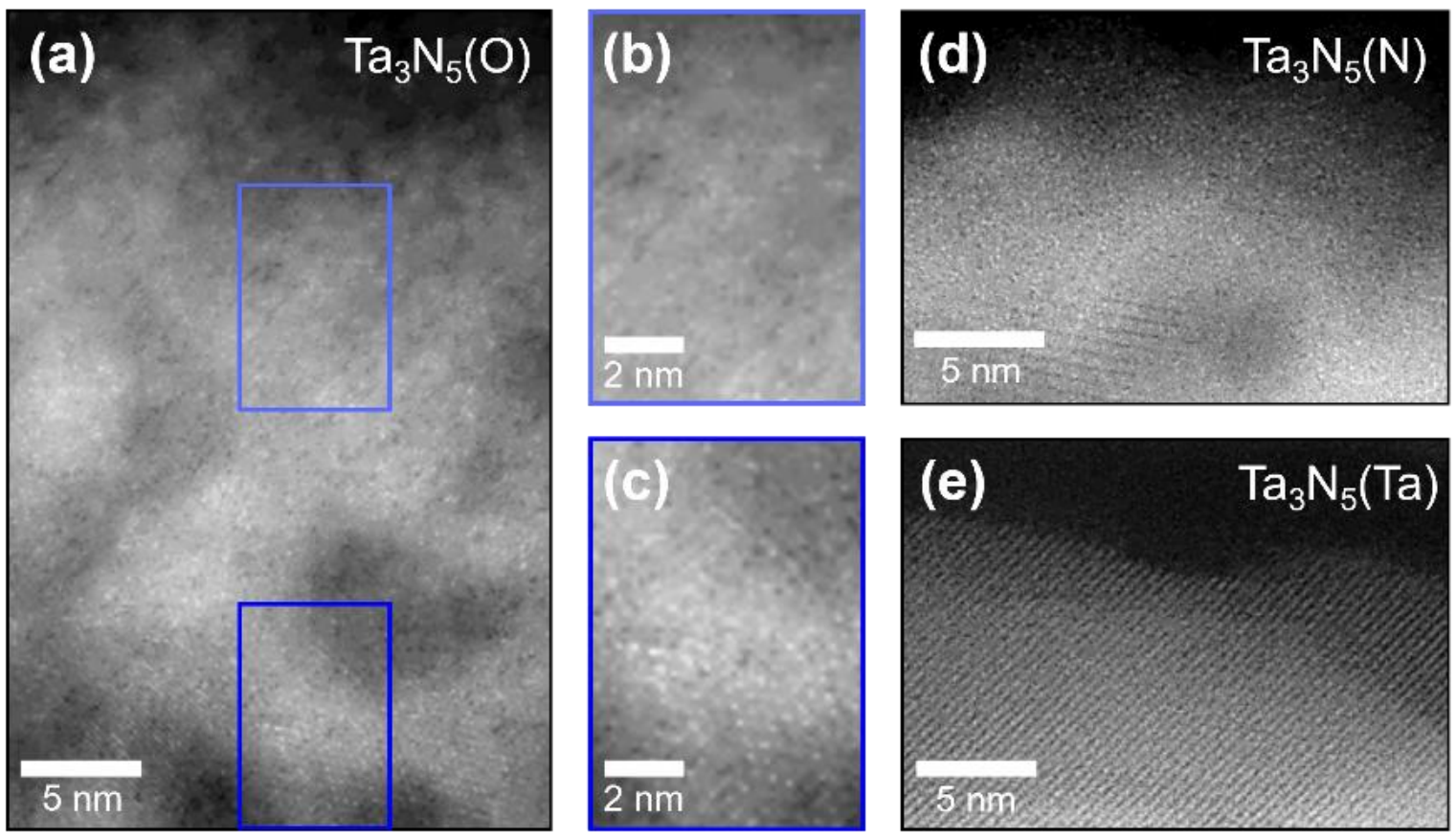

Figure 2. Near-surface structures of Ta_3N_5 thin films as a function of the different thin film precursors. Cross-sectional HAADF STEM images of (a) Ta_3N_5(O), (d) Ta_3N_5(N), and (e) Ta_3N_5(Ta). (b) and (c) are zoom-in images of the marked areas in (a).

We added the Mott-Schottky measurements for Ta_3N_5(O) in Figure S16.

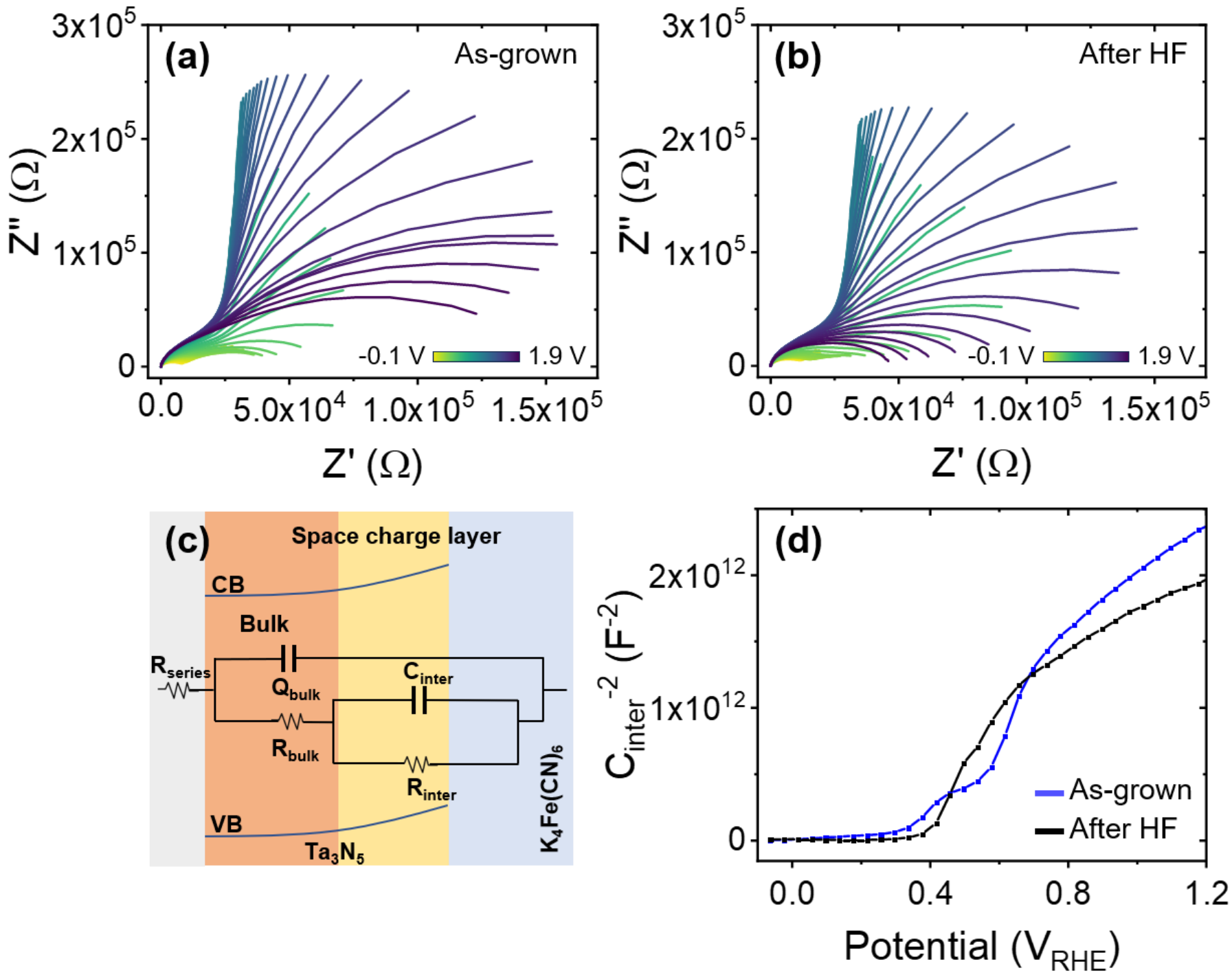

Figure S16. Electrochemical impedance spectroscopy of Ta_3N_5(O) was measured over a frequency range from 1 Hz to 100 kHz under dark conditions. Nyquist plots recorded over a potential range from -0.1 V_{RHE} to 1.9 V_{RHE} (a) without and (b) with HF treatment. (c) Equivalent circuit used for fitting, comprising the series resistance R_{series}, bulk (Q_{bulk}, R_{bulk}) and interface (C_{inter} and R_{inter}) contributions. (d) Mott-Schottky plots derived from capacitance values obtained from fitting the potential-dependent EIS data. In the model, R_{series} accounts for ohmic contributions from contacts and wiring. The bulk is described by a resistance R_{bulk} to charge transport through the semiconductor bulk, and a non-ideal capacitive element Q_{bulk}, is used to account for inhomogeneities. The interface is described by a capacitance C_{inter} between the electrode and the electrolyte, and the charge-transfer resistance R_{inter}.

We included the data in the discussion on page 11:

"To link changes in surface properties to charge transport/transfer characteristics, we performed both electrochemical impedance spectroscopy (EIS) and photoelectrochemical impedance spectroscopy (PEIS) on Ta_3N_5(O) thin films with and without HF treatment in 1 M KP_i and 0.1 M $K_4Fe(CN)_6$ as a hole scavenger. In detail, we recorded EIS over a potential range from -0.1 V_{RHE} to 1.6 V_{RHE} and a frequency range from 1 Hz to 100 kHz (Fig. S16). The data were fitted with an equivalent circuit to describe Ta_3N_5 in contact with the electrolyte (Fig. S16c). The assignment of the circuit elements, as shown below, is consistent with previous EIS interpretations for Ta_3N_5 in literature.[5–7] We used the fitted capacitances from broadband EIS to derive the Mott-Schottky plot (C^{-2} vs. V_{RHE}) of Ta_3N_5(O) with and without HF treatment. For as-grown Ta_3N_5(O), we observe a two-slope behavior, with a change in slope at higher applied potentials (Fig. S16d). This behavior, previously observed in Ta_3N_5, suggests that band bending responds to the applied potential to varying degrees across different potential regions.[6] In the low potential region, the response is consistent with Fermi level pinning due to the existence of a distribution of surface states. At more anodic potentials, partial depinning of the Fermi level leads to a stronger dependence of the band bending on the potential, corresponding to a depletion-dominated regime.[6] After HF treatment, the Mott–Schottky response approaches the single-regime behavior over the investigated potential range."

We added the PEIS measurements for Ta_3N_5(O) in Figure S17.

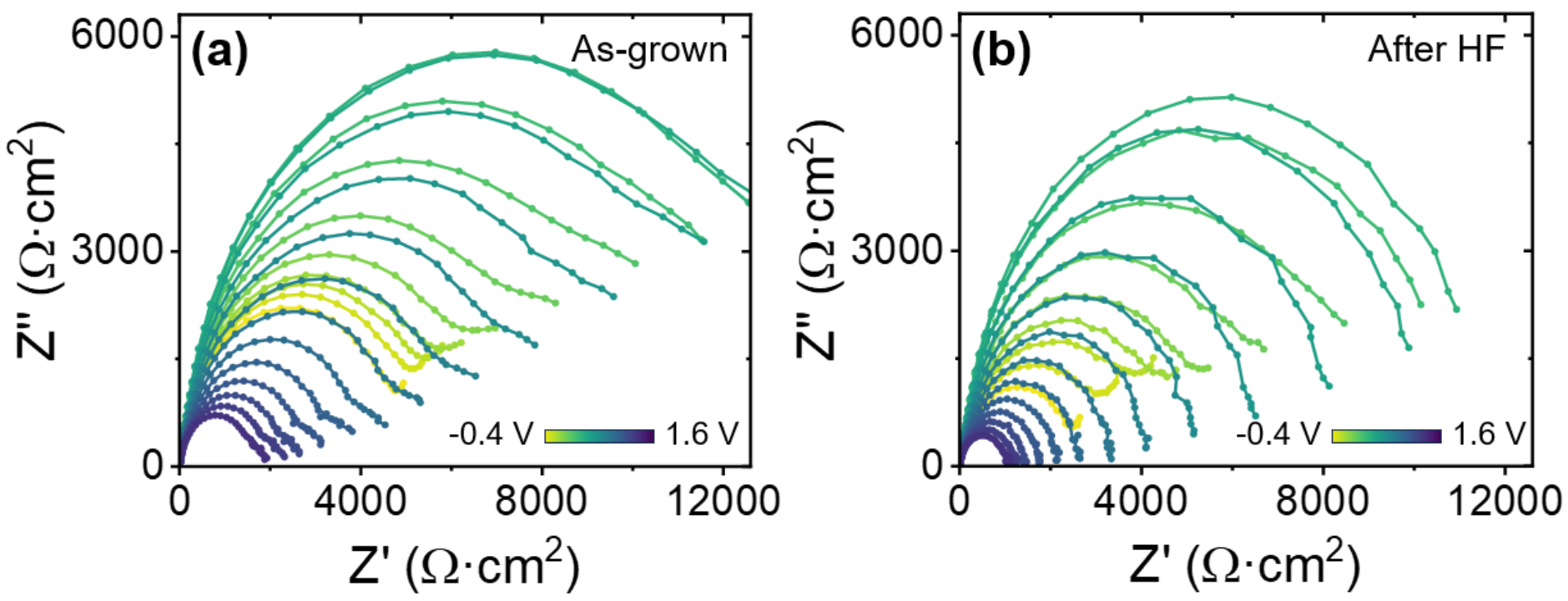

Figure S17. Photoelectrochemical impedance spectroscopy of Ta_3N_5(O) over a frequency range from 1 Hz to 100 kHz under illumination**.** Nyquist plots recorded over a potential range from -0.1 V_{RHE} to 1.9 V_{RHE} (a) without and (b) with HF treatment.

Furthermore, we improved the discussion on page 11:

"Complementary PEIS measurements show that the radius decreases at comparable applied potentials after HF treatment (Fig. S17), indicating a lower overall resistance under operating conditions and more favorable photoelectrochemical charge-transfer characteristics."

Comment 1.7: In Figure 2a and c, there is no sample information, and the colors of curves are so similar that it is not easy to identify them immediately.

Changes: Since we added an additional figure in the revised manuscript, Fig. 2 is now Fig. 3. We updated the color scale in Figs. 3 and 5 to improve readability, since both images use

the same colors. Additionally, labels were added to Fig. 3a and c. The new figures are reproduced below for clarity.

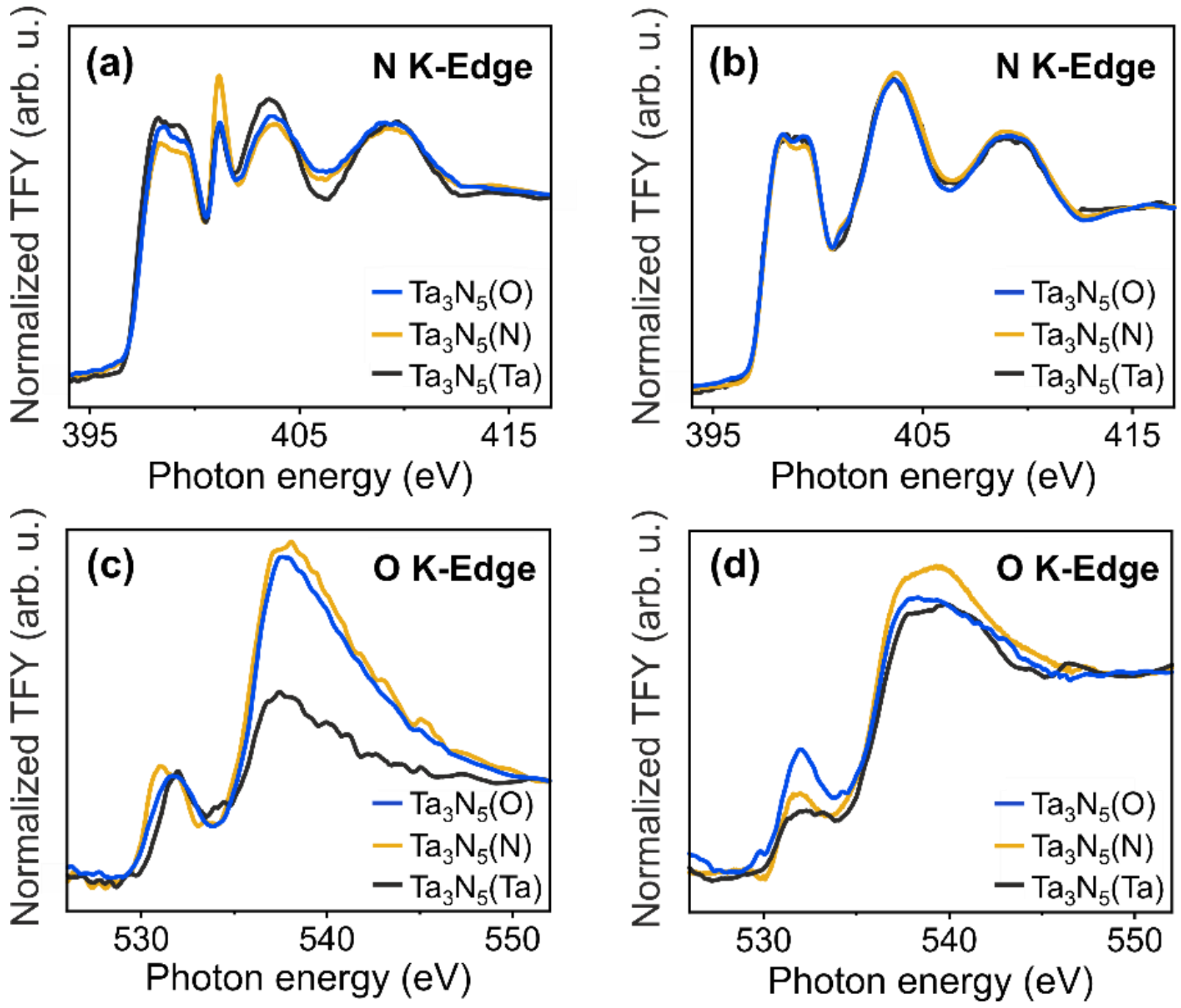

Figure 3. XAS data of the N and O K-edges of Ta_3N_5 thin films as a function of the different precursors. Normalized N and O K-edge in (a, c) TFY and (b, d) TEY mode showing the bulk and surface properties of Ta_3N_5(O), Ta_3N_5(N), and Ta_3N_5(Ta), respectively.

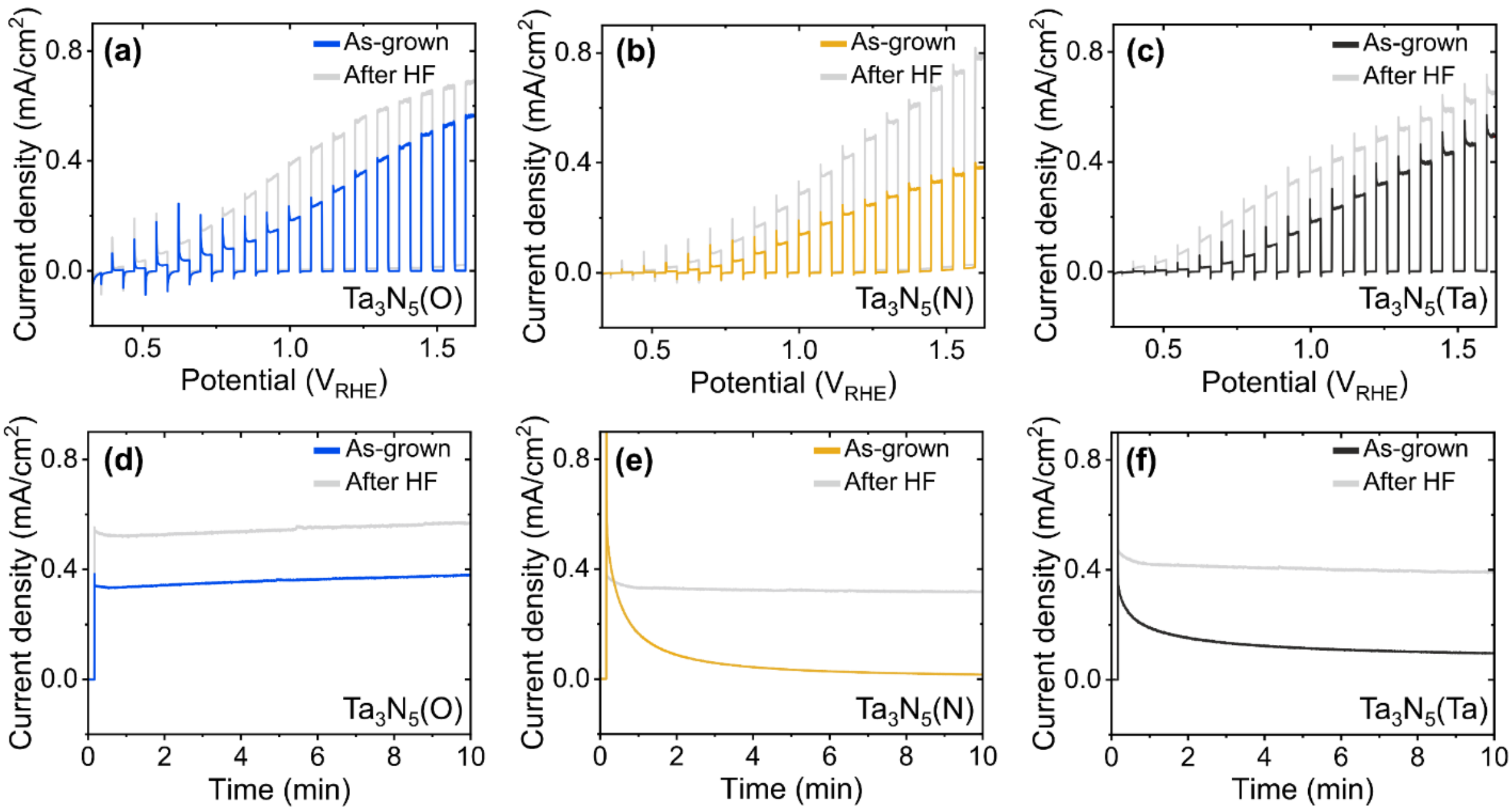

Figure 5. Photoelectrochemical performance characteristics before and after surface treatment of Ta_3N_5 thin films as a function of the different precursors. Linear sweep voltammetry and chronoamperometry measurements for 10 min at 1.23 V_{RHE} of (a, d) Ta_3N_5(O), (b, e) Ta_3N_5(N), and (c, f) Ta_3N_5(Ta) thin films under chopped illumination in 1 M KP_i (pH 12.3) in the presence of 0.1 M $K_4Fe(CN)_6$ as a hole scavenger, before and after the 1 min HF treatment.

Response to reviewer 2

The manuscript presents a detailed investigation into the differentiation of surface and bulk defect states in Ta_3N_5 photoanodes using a suite of depth-sensitive structural, chemical, and optoelectronic characterization methods. The study is well structured and supported by substantial experimental data; however, several aspects of the interpretation, validation of PEC performance, quantitative analysis, and mechanistic justification require further strengthening before the manuscript can meet the publication standard of Nature Communications.

We thank the reviewer for their positive assessment of the manuscript and for recognizing the quality of the structure and analysis of the results. In addition, we appreciate their constructive comments, which have helped us to further strengthen the interpretation, clarify the mechanistic insights, and improve the overall presentation of the work.

Comment 2.1: The authors state that HF-treated samples exhibit improved stability, but no long-term durability studies are shown. It is recommended to extend the stability test to at least 10 hours.

Answer: We agree with the reviewer that the 10 min chronoamperometry experiments are short for durability testing. Therefore, for all three HF-treated films, we have now performed continuous 10 h chronoamperometry tests in 1 M KP_i with 0.1 M $K_4Fe(CN)_6$ as hole scavenger, measured at 1.23 V_{RHE} under illumination (AM 1.5G). The improved stability after HF treatment persists even after 10 h under these oxidation conditions, in contrast to the much faster degradation of Ta_3N_5(N) and Ta_3N_5(Ta) thin films without HF treatment.

Changes: We added the 10 h durability measurements as Fig. S6.

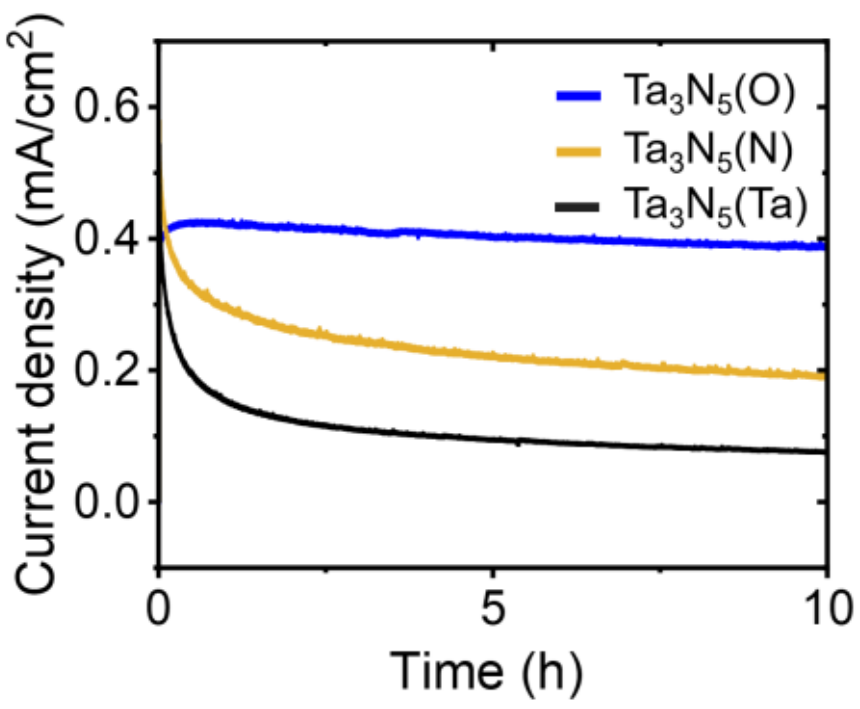

Figure S6. Long-term chronoamperometry tests of Ta_3N_5(O), Ta_3N_5(N), and Ta_3N_5(Ta) thin films after HF treatment, measured at 1.23 V_{RHE} under illumination (AM 1.5G) in 1 M KP_i (pH 12.3) with 0.1 M $K_4Fe(CN)_6$ as hole scavenger.

We included the results in the discussion on page 8:
"After 10 h of operation, Ta_3N_5(O) still shows stable operation, while a decay in the photocurrent density is observed for Ta_3N_5(N) and Ta_3N_5(Ta)."

Comment 2.2: No chemical analysis of post-stability samples is provided to confirm retention of nitrogen and prevention of further oxidation. Since Ta-based nitrides are known to undergo photocorrosion, the configuration after stability testing should be analyzed with XPS or Raman.

Answer: We thank the reviewer for the suggestion and have now conducted complementary XPS measurements after 10 h of chronoamperometry for HF-treated films. XPS core levels of Ta 4f, N 1s, and O 1s were recorded and analyzed. For all thin films, we observe a change in the N/Ta ratio and the O(530 eV)/Ta ratio, indicating slight oxidation under ferrocyanide oxidation after HF treatment. Surface oxidation is most pronounced for Ta_3N_5(N) and Ta_3N_5(Ta) compared to Ta_3N_5(O). Interestingly, after HF treatment, a measurable current density is still maintained after 10 h of operation, despite slight surface oxidation. Comparing XPS spectra of Ta_3N_5 photoelectrodes without HF treatment after a 1 h durability test from our previous study[1] and with HF-treated Ta_3N_5 photoelectrodes after the 10 h durability test from this study indicates that higher N/Ta ratios are maintained for HF-treated films despite the 10-times longer testing duration. Overall, this indicates that HF treatment reduces surface oxidation of Ta_3N_5 photoelectrodes.

Changes: We included XPS measurements after 10 h of stability testing in the hole scavenger for all three thin films in Fig. S7. Additionally, we summarized the corresponding ratios in Table S4.

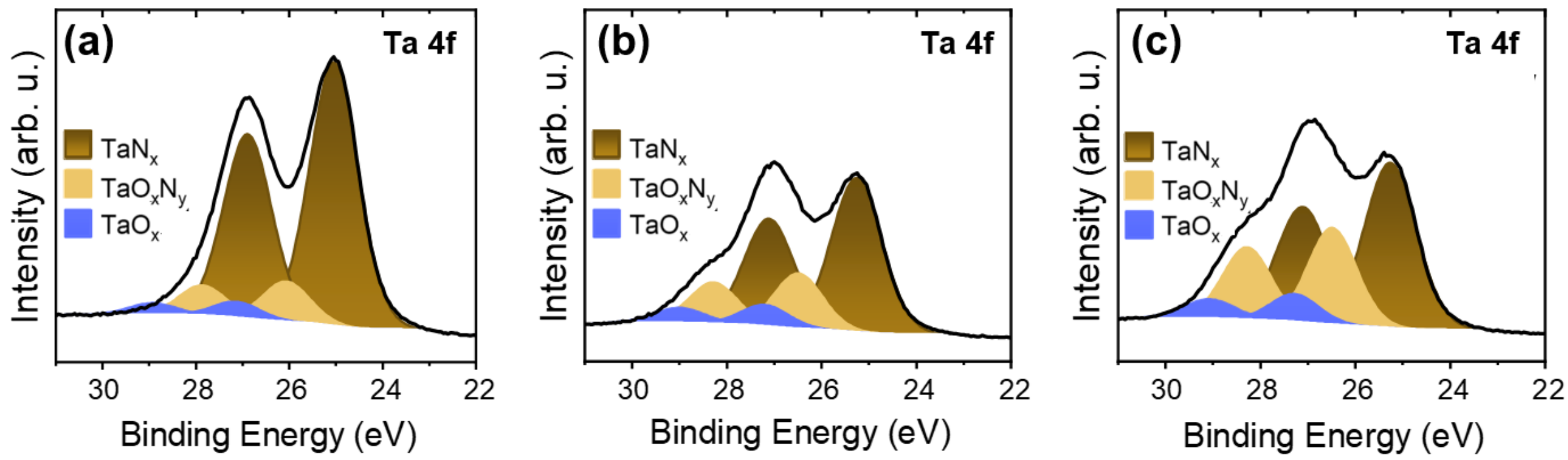

Figure S7. High-resolution XPS spectra of HF-treated tantalum nitride thin films after 10 h stability testing with hole scavenger. Ta 4f spectra of (a) Ta_3N_5(O), (b) Ta_3N_5(N), and (c) Ta_3N_5(Ta).

Table S3. Atomic ratios O/Ta and N/Ta derived from XPS core-level spectra (O 1s, N 1s, Ta 4f) for all thin films directly after synthesis. Additionally, the relative contributions of TaN_x and TaO_xN_y obtained from the Ta 4f spectra are shown. For the oxygen content, only the peak at 530 eV, corresponding to Ta-O bonds, was considered.

Sample	O/Ta	N/Ta	TaN_x (at.%)	TaO_xN_y (at.%)
Ta_3N_5(O)	0.28	1.38	87.6	12.4
Ta_3N_5(N)	0.11	1.65	92.6	7.4
Ta_3N_5(Ta)	0.11	1.70	93.0	7.0

Table S4. Atomic ratios O/Ta and N/Ta derived from XPS core level spectra (O 1s, N 1s, Ta 4f) for all thin films directly after synthesis. Additionally, the relative contributions of TaN_x and TaO_xN_y obtained from the Ta 4f spectra are shown. For the oxygen content, only the peak at 530 eV, corresponding to Ta-O bonds, was considered.

Sample	O/Ta	N/Ta	TaN_x (at.%)	TaO_xN_y (at.%)	TaO_x (at.%)
Ta_3N_5(O)	0.45	1.22	76.6	17.2	6.2
Ta_3N_5(N)	1.01	0.85	65.1	19.0	15.9
Ta_3N_5(Ta)	1.32	0.73	55.1	31.8	13.1

Furthermore, we improved the discussion on page 8/9:
"Complementary XPS measurements after 10 h of stability testing reveal changes in the N/Ta and O(530 eV)/Ta ratios (Table S4, Fig. S7), indicating slight surface oxidation following sustained ferrocyanide oxidation by the HF treated electrodes. Surface oxidation is most pronounced for Ta_3N_5(N) and Ta_3N_5(Ta) compared to Ta_3N_5(O). Comparing XPS spectra of Ta_3N_5 photoelectrodes without HF treatment after a 1 h durability test from our previous study[1] with HF-treated Ta_3N_5 photoelectrodes after the 10 h durability test from this study indicates that higher N/Ta ratios are maintained for HF-treated films despite the 10x longer testing duration. Overall, this indicates that HF treatment reduces surface oxidation of Ta_3N_5 photoelectrodes."

Comment 2.3: The central claim is that surface disorder and deep-level traps dominate PEC behavior, but there is no quantitative defect density extraction via Mott–Schottky slopes or Deep Level Transient Spectroscopy (DLTS). The argument remains descriptive rather than mechanistic. The authors should quantify defect densities to strengthen the cause–effect link.

Answer: We agree with the reviewer that DLTS would be a perfect addition. Generally, DLTS analysis requires a well-defined rectifying junction with an asymmetric contact configuration. For Ta_3N_5 photoanodes, we found that achieving a reliable asymmetric device contact without altering the near-surface chemistry/defect landscape is experimentally challenging. In this context, we tested gold, titanium, aluminum, and platinum as contacts but could not achieve the required Schottky contact characteristics. Furthermore, attempts to grow a closed dielectric layer on Ta_3N_5 were unsuccessful. Due to these synthesis challenges, we were unfortunately not able to measure DLTS.

We agree that Mott-Schottky analysis is a valuable addition to this work, and inspired by questions from all three reviewers, we have now performed both electrochemical impedance spectroscopy (EIS) in the dark and photoelectrochemical impedance spectroscopy (PEIS) under illumination. For a discussion of the EIS and PEIS results and changes to the manuscript, please see the response to Comment 1.6 (Reviewer 1).

Lastly, we agree with the reviewer that the quantitative link between defects and PEC behavior is important. Therefore, we are currently working on another manuscript using

transient absorption spectroscopy to establish property-defect relationships. This complex analysis is outside of the scope of the present study.

Comment 2.4: The authors attribute PEC improvements to crystallization and oxygen removal. The question to be answered is does HF simply etch away amorphous oxides, or does it also introduce fluorine species on the surface?

Answer: We agree with the reviewer that the possible impact of fluoride ions on performance should be discussed. The thin films were treated with HF (5%) for 1 min and subsequently rinsed with DI water. To identify the fluoride surface termination, we performed XPS measurements of the F 1s core level on the thin films directly after growth, after HF treatment, and after the PEC stability test. In detail, we confirmed that no fluoride ions are present at the surface of the as-grown films. As suggested by the reviewer, after HF-treatment, we do observe the adsorption of fluoride ions on the surface. However, after PEC stability tests the fluoride peak disappears again for all three thin films. Accordingly, the F 1s contribution is not present after PEC testing for 10 h in 1 M KP_i with hole scavenger, while the stability is still improved. Additionally, we tested the photoelectrode immersing for 1 min in 1 M KP_i with hole scavenger in the dark. The F 1s signal decreases strongly indicating the rapid loss of the fluoride-related contribution. Accordingly, the fluoride ions are only weakly bound to the surface and are unstable under the aqueous conditions relevant to PEC testing.

These findings strongly suggest that the surface termination with fluoride ions is not the main factor contributing to enhanced stability. Rather, we correlate the improvement to removal/thinning of the disordered near-surface region (GIWAXS), reduced O(530 eV)/Ta (XPS), and increased hydrophilicity (contact angle). Furthermore, to understand the impact on charge transfer and transport, additional EIS measurements are included, as described in Comment 1.6.

Changes: We included the F 1s core level spectra as Figure S12 in the supporting information.

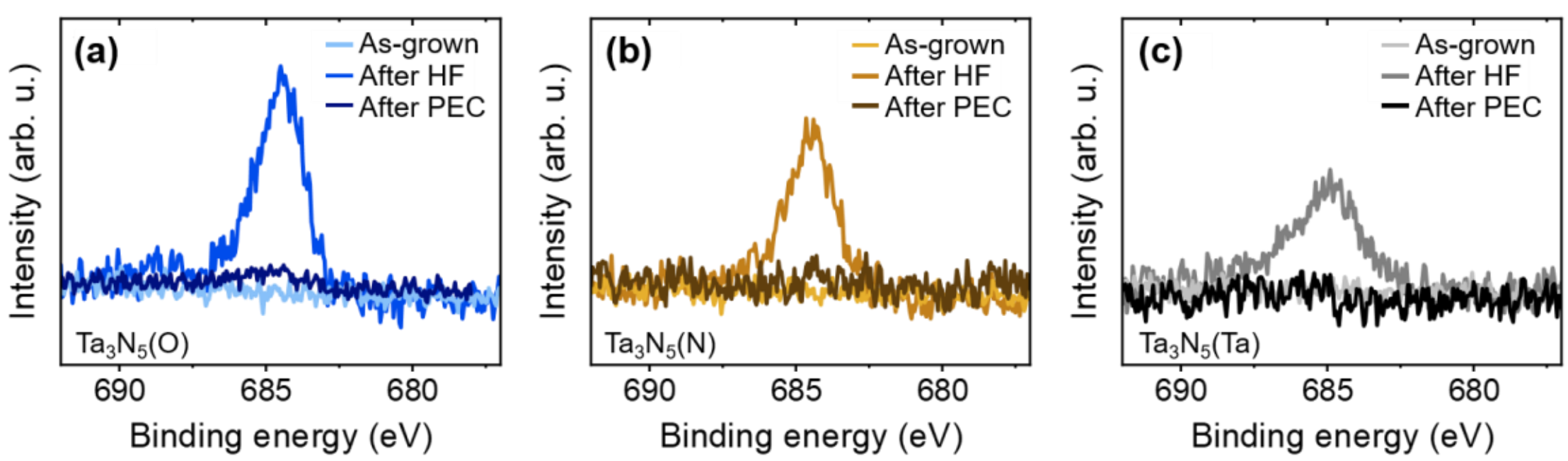

Figure S12. High-resolution F 1s core level spectra of (a) Ta_3N_5(O), (b) Ta_3N_5(N), and (c) Ta_3N_5(Ta) before and after HF treatment, as well as after HF etching and PEC testing for 10 h in 1 M KP_i with hole scavenger.

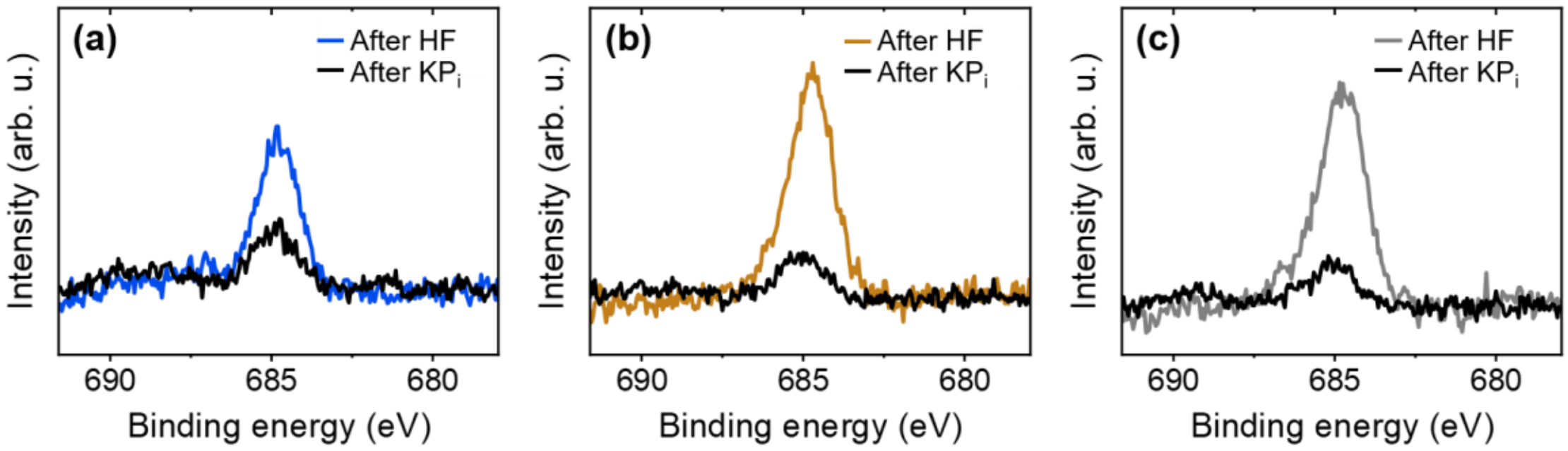

Figure S13. High-resolution F 1s core level spectra of (a) Ta_3N_5(O), (b) Ta_3N_5(N), and (c) Ta_3N_5(Ta) after HF treatment, as well as 1 min in electrolyte.

Furthermore, we improved the discussion on page 10:

"Additionally, we analyzed the F 1s and O 1s to better understand the surface properties of the different Ta_3N_5 thin films. In the F 1s spectra (Fig. S12), we observed the presence of fluoride ions only after HF treatment for all films. In the O 1s spectra, hydroxyl groups and exposed regions of silicon terminated by sub-oxides have overlapping signals at ~532 eV. To understand the presence of hydroxyl groups on the surface, the contributions need to be deconvoluted (Table S6). The remnant photoemission intensity after subtracting the silicon oxide contribution suggests that hydroxyl groups are also present at the surface after HF treatment. Additionally, we analyzed the chemical composition after PEC testing for 10 h and immersion for 1 min in 1 M KP_i with a hole scavenger (Fig. S12 and S13), which shows the fast disappearance of the F 1s contribution on the surface, while the stability is still improved. These findings indicate that fluoride ion absorption on the surface is not the main reason for the observed stabilization."

Comment 2.5: The manuscript concludes that the surface defects suppress charge transfer, yet there is no electrochemical impedance spectroscopy (EIS). Such data would be essential to substantiate statements about surface-mediated recombination and transport limitations.

Answer: We thank the reviewer for suggesting EIS measurements to investigate interfacial charge transfer characteristics. In this context, it is important to note that Ta_3N_5(N) and Ta_3N_5(Ta) are not stable under ferrocyanide conditions without HF treatment (Fig. 4), which is essential for reliable EIS measurements. Therefore, we only measured EIS for Ta_3N_5(O) before and after HF at different potentials under illumination, given its stability. For the discussion of the EIS and PEIS results and changes to the manuscript, please see the response to Comment 1.6 (Reviewer 1).

Response to reviewer 3

This work reports the preparation of orthorhombic Ta_3N_5 using three different precursors. Based on prior studies, the stability of Ta_3N_5 is known to depend strongly on the choice of precursor. The authors employ GIWAXS, ERDA, and depth-dependent photoluminescence (PL) to reveal pronounced differences in defect types and concentrations between the surface and bulk regions, which are attributed to compositional variations. Ta_3N_5 derived from TaO_x exhibits enhanced photoelectrode kinetic stability, which the authors ascribe to passivation by a disordered surface layer. They further propose that deep-level defects deteriorate kinetic stability by hindering interfacial charge injection and promoting trapping-mediated self-oxidation. In addition, HF etching is applied to all samples, resulting in hydroxyl-terminated surfaces. This treatment improves surface hydrophilicity, induces surface passivation, and consequently enhances both catalytic stability and photoelectrochemical performance. Despite these merits, several issues require clarification or further evidence:

We thank the reviewer for their careful reading of the manuscript and for their thoughtful and technically insightful comments, which have helped us to clarify key aspects of the interpretation and further strengthen the manuscript.

Comment 3.1: Reference 4 does not report the presence of a disordered surface layer in as-synthesized, fresh Ta_3N_5 samples. The authors should therefore clarify the origin of the disordered surface layer observed in Ta_3N_5(O). Additional structural characterization of this layer—such as cross-sectional TEM—would be highly valuable.

Answer: We thank the reviewer for the comment and have now conducted additional cross-sectional HAADF STEM measurements to address this point. In agreement with our XAS and GIWAX results, cross-sectional HAADF STEM confirms the formation of a disordered surface layer for Ta_3N_5(O) and Ta_3N_5(N), while a clear crystalline structure is observed for Ta_3N_5(Ta). In detail, the amorphous surface layer is significantly thicker on Ta_3N_5(O) compared to Ta_3N_5(N).

More generally, the material properties and structure of Ta_3N_5 are sensitive to the synthesis route, the chemical composition of the precursor, and the nitridation conditions. In reference 4, Ta_3N_5 nanotubes were synthesized by anodizing Ta foil to form tantalum oxide nanotubes that were subsequently converted to Ta_3N_5 via NH_3 annealing, yielding a crystalline surface structure, as shown by cross-sectional HAADF STEM. In contrast, our study focuses on polycrystalline thin films derived from sputtered amorphous thin films with different chemical compositions. Thus, these systems differ not only in precursor type and morphology, but also in nitridation conditions (e.g., pressure, flow, and annealing times), all of which can influence near-surface disorder. For example, in a previous study, we showed that extended annealing times can lead to defect formation.[8]

Importantly, the morphology of the precursor can play a role in the nitridation process. For nanotubes, conversion can initiate from both the surface and sidewalls, which are all exposed to NH_3 during ammonolysis, and the structure can expand or contract more freely due to the internal void volume and small contact area with the substrate. In contrast, thin films are laterally confined and convert primarily from the upper surface, which can introduce additional stress and affect the conversion process. Overall, these different reaction conditions and morphologies could impact the formation of an amorphous layer.

In this context, it is also important to note that the conversion of TaO_x into Ta_3N_5 is associated with an increase in mass density, leading to a volume contraction, whereas the conversion of TaN_x and Ta into Ta_3N_5 is characterized by a reduction in mass density, leading to a volume expansion. In our study, we observed improved surface crystallinity for systems undergoing volume expansion, whereas volume contraction appears to be correlated with the formation of an amorphous layer. Although the exact chemical composition and density of the precursor are not provided in Reference 4, limiting our ability to provide a direct comparison between the different conversion processes, these observations suggest that both the synthesis pathway and associated volume changes play an important role in defining the near-surface structure.

Changes: To address the reviewer's comment, we performed HAADF STEM measurements and present them in Fig. 2, together with a discussion on page 4:

"Complementary to GIWAX, we investigated the near-surface structure of the three photoelectrodes by cross-sectional scanning transmission electron microscopy (STEM). High-angle annular dark field (HAADF) STEM images (Fig. 2) reveal an amorphous surface layer on both Ta_3N_5(O) and Ta_3N_5(N), with a thickness exceeding 10 nm for Ta_3N_5(O) but approximately 5 nm on Ta_3N_5(N). In contrast, Ta_3N_5(Ta) exhibits a crystalline surface."

On page 5: "The formation of an amorphous surface layer is likely impacted by different synthesis methods, precursor compositions, and conversion conditions, as well as geometric constraints during ammonolysis. For example, the conversion of TaO_x into Ta_3N_5 is associated with an increase in mass density, leading to a volume contraction, whereas the conversion of TaN_x and Ta into Ta_3N_5 is characterized by a reduction in mass density, leading to a volume expansion. In addition, thin films are laterally constrained by the substrate and conversion proceeds from the surface, whereas nanostructures, such as nanotubes, have significantly larger gas/solid interface areas from which the transformation can proceed and open internal volumes that can better accommodate density changes. In addition to such factors, comparison of these structural changes with the chemical compositions of the films indicates that increased surface disorder is correlated with increasing oxygen content in the precursor films."

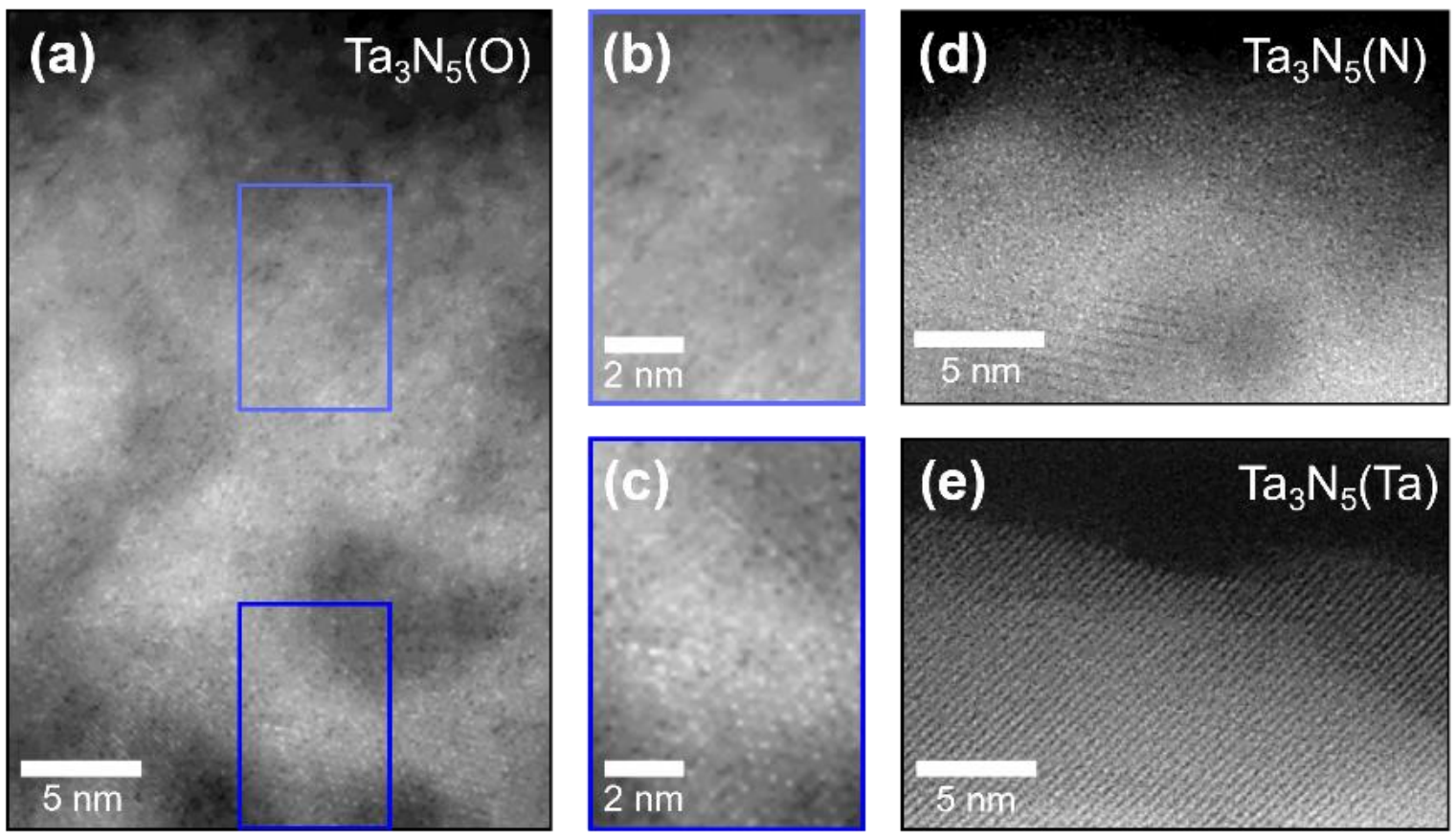

Figure 2. Near-surface structures of Ta_3N_5 thin films as a function of the different thin film precursors. Cross-sectional HAADF STEM images of (a) Ta_3N_5(O), (d) Ta_3N_5(N), and (e) Ta_3N_5(Ta). (b) and (c) are zoom-in images of the marked areas in (a).

The corresponding experimental details were included in the Method Section:
"STEM measurements were performed at an FEI Titan Themis 80–300 (S)TEM, operated at 200 kV, equipped with an XFEG field emission gun, the aberration corrector CEOS DCOR and the chemiSTEM SuperX detection system for energy-dispersive X-ray spectroscopy. High-angle annular dark field (HAADF) STEM images were acquired using a semi-convergence angle of the scanning probe of 20.0 mrad and a Fischione Model 3000 ring detector covering scattering angles of 35 – 200 mrad at the used camera length of 195 mm. TEM-lamellae were prepared by a standard milling and lift-out procedure, followed by thinning and polishing steps, inside an SEM FEI Helios NanoLab G3 UC equipped with the MultiChem gas delivery system using a Pt-source and a Tomahawk ion column generating a focused Ga-ion beam. TEM-lamellae were cut from sputtered films grown on Si wafers and attached to molybdenum FIB lift-out grids for TEM usage. Before preparation, an approximately 20 nm carbon film was evaporated onto each wafer using the compact-coating-unit-010."

Comment 3.2: GIWAXS results clearly indicate an amorphous surface region within approximately the top ~3 nm of Ta_3N_5. The authors attribute this amorphization to increased oxygen content and enhanced surface disorder. However, ERDA measurements show comparable O and N concentrations among all three samples down to a depth of ~8 nm. This apparent inconsistency should be addressed, and the authors should clarify whether the amorphous surface layer arises from subtle compositional differences, local bonding environments, or other structural factors.

We thank the reviewer for pointing this out. Indeed, the data show a gradual increase in oxygen and nitrogen concentrations in the first ~10 nm, which is due to the limited depth resolution of ERDA. As a result, the physical profiles are convolved with the system resolution and physical effects such as straggling. Therefore, we only used ERDA to indicate the general trend in the distribution of oxygen and nitrogen within the film without focusing

on the exact depth profile. The important information we gain from ERDA measurements is that nitrogen is rather homogeneously distributed across the film, while higher oxygen concentrations are observed in the near-surface region than in the bulk. The gained insights are further supported by XPS measurements.

The comment motivated a much closer inspection of the ERDA data. Through this analysis, we discovered that a sample naming error led to the inclusion of the incorrect dataset for Ta_3N_5(O) and Ta_3N_5(N). In the revised version, we included the correct data and improved the analysis. While the key interpretations are not affected, the correct data are in better agreement with the insights gained from the other techniques. Specifically, we observe significantly higher oxygen content in the bulk and at the surface for Ta_3N_5(O) and Ta_3N_5(N), whereas it is more homogeneously distributed in Ta_3N_5(Ta). Overall, this inadvertent error motivated us to carefully review all other data sets presented, and we can verify that they are correct.

Changes: We updated Fig. S2 in the supporting information.

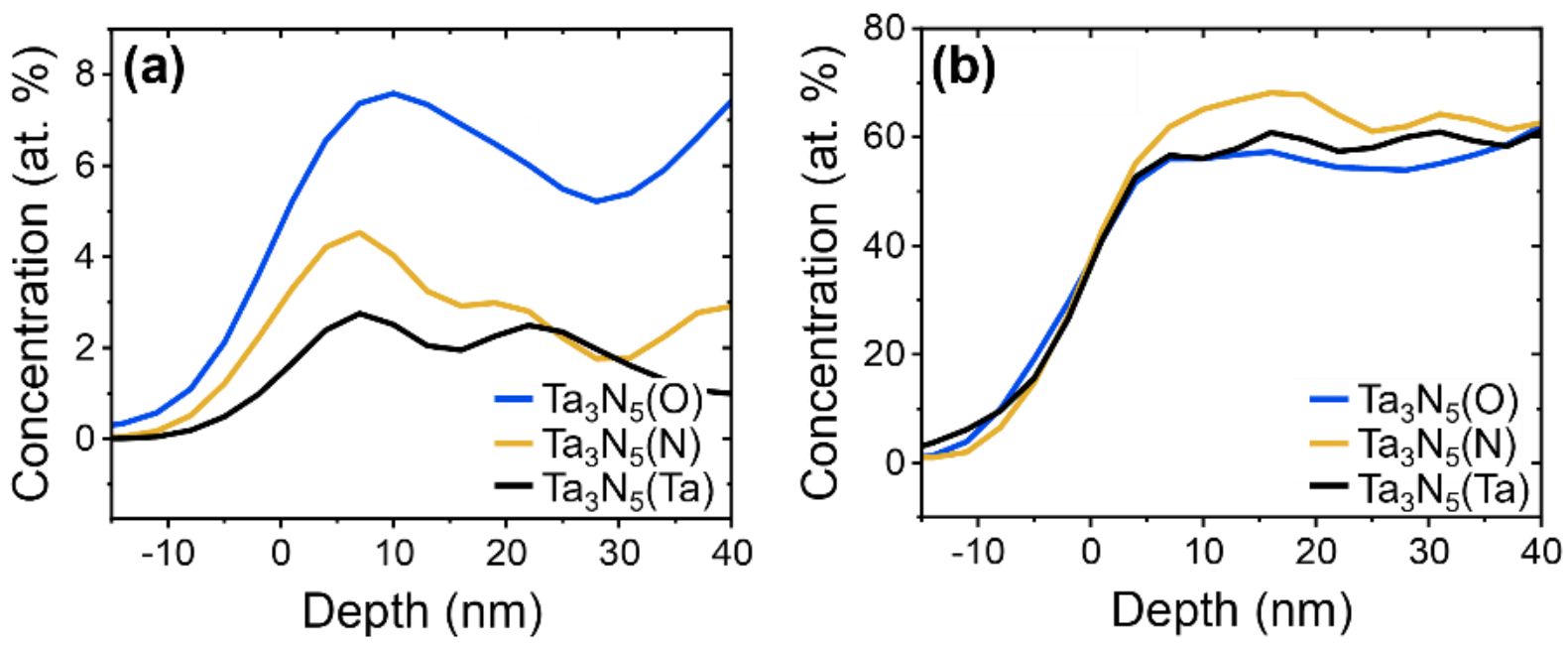

Figure S2. Depth-resolved elemental composition of Ta_3N_5 thin films synthesized from different precursors. (a) Oxygen and (b) nitrogen concentration profiles obtained by ERDA.

Furthermore, we improved the discussion on page 3:
"For ERDA measurements, it is important to note that the gradual increase in element concentrations in the first ~10 nm arises from the limited depth resolution of the detection system, broadening the depth profile. Therefore, we only use ERDA to reveal general trends in the distribution of oxygen and nitrogen near the surface and within the bulk of the films. We observe an increase of near-surface oxygen content relative to bulk, which is most pronounced for Ta_3N_5(O) with the highest oxygen content, and is reduced for Ta_3N_5(N) and Ta_3N_5(Ta), which contain lower oxygen concentrations. In contrast, the nitrogen content exhibits a more homogeneous distribution throughout the bulk. Overall, these results show a pronounced difference between surface and bulk compositions, with these variations depending sensitively on the synthesis route."

Comment 3.3: The criteria used to define the boundary between the "surface" and "bulk" regions should be explicitly stated. What depth or physical parameters were used to distinguish these two regimes in the analysis?

Answer: Indeed, we did not define an explicit depth of the boundary between the surface and bulk regions, since the thickness of the surface layer and the position of the surface-bulk boundary depend on the precursor. Our aim is to show that the surface and bulk properties are different, but not on exactly defining the point of transition. In our study, we combined techniques with varying penetration depths and, in other instances, conducted depth-dependent measurements. Therefore, the difference between the surface and the bulk that we report depends strongly on the depth resolution of the respective measurements.

- For chemical composition, we used depth-dependent ERDA measurements, where we used the complete signal from the whole film to determine the bulk composition. In addition, the surface composition was determined with XPS with an average detection depth of about 5 nm.
- For the structure, we used depth-dependent GIXRD and analyzed the signal for a depth of 3 nm for the surface and 38 nm for the bulk.
- TEY-XAS is typically associated with a detection depth of less than 10 nm, depending on the exact element, while TFY-XAS is associated with a detection depth larger than our film thickness and is thus bulk-sensitive.
- In PL, we cannot clearly define the depth from which the signal arises. However, we can clearly distinguish between the near-surface region and the bulk-like region.

Taken together, except for PL, the signal obtained for the “surface” is from a depth of about 0 - 10 nm from the surface. Clearer insight into the transition from bulk to surface can be obtained from the added cross-section TEM measurements (see Comment 3.1).

Changes: Considering the large number of different methods applied in this work, we have now added an additional table to the SI, highlighting the different probing depths of the different techniques.

Table S1. Probed regions of the different techniques used for surface and bulk material characterization in this work.

	Surface	Bulk	Reference
Chemical composition	**XPS**<br>~ 5 nm	**ERDA**<br>Signal from complete film<br>Resolution ~10 nm	[9,10]<br>[11]
Structure	**GIWAX**<br>~ 3 nm	**GIWAX**<br>~ 38 nm	[12,13]
XAS	**TEY**<br>Element-dependent: ~ 10 nm	**TFY**<br>Multiple hundred nm	[14,15]
PL	Focus point near surface	Focus point in bulk	[16]

We included these considerations in the main text on page 3:
"To this end, we combine multiple techniques with different probing depths (Table S1), with surface-sensitive measurements typically probing the near-surface region within 5-10 nm. In addition, we note that the transition between surface and bulk properties is not sharply defined and can vary between the three films due to differences in precursor composition, defect distributions, and resulting structural inhomogeneities."

Comment 3.4: It remains unclear whether the disordered surface layer in Ta_3N_5(O) exhibits electronic and chemical properties similar to those of the well-crystallized Ta_3N_5(Ta), given their markedly different defect concentrations. The depth range of the ~10 nm amorphous surface layer in Ta_3N_5(O) should be clearly indicated and discussed.

Answer: We thank the reviewer for this interesting question regarding the chemical and electronic properties of the disordered surface layer. To identify differences in the chemical compositions of all three thin films, we conducted surface-sensitive XPS (Fig. S8–S10). Among the three thin films (Table S3), Ta_3N_5(O) has the highest surface O/Ta ratio of 0.28, while it is reduced for Ta_3N_5(N) to 0.11, and for Ta_3N_5(Ta) to 0.11. Furthermore, the N/Ta ratio increases from 1.38 for Ta_3N_5(O) to 1.65 for Ta_3N_5(Ta) and 1.70 for Ta_3N_5(N). In short, the composition of Ta_3N_5(Ta) is closest to the ideal stoichiometry of Ta_3N_5, while Ta_3N_5(O) is characterized by increased oxygen content and Ta_3N_5(N) by increased nitrogen content. Additionally, in the Ta 4f spectra (Table S3), we also observe the tantalum nitride component, with the highest contribution for Ta_3N_5(Ta) (93.0%), which is reduced to 92.6% for Ta_3N_5(N) and 88% for Ta_3N_5(O). We have now included the results more clearly in the discussion.

The depth of the surface layers is clearly discernible in the penetration depth profile in Fig. 1g, h, and i. Furthermore, we added additional STEM measurements to visually support these insights in Fig. 2 (see comment 3.1 for details).

We thank the reviewer for suggesting the investigation of the electronic properties. In principle, (P)EIS measurements could provide insight into the interfacial charge transfer characteristics. However, as-grown Ta_3N_5(N) and Ta_3N_5(Ta) are not sufficiently stable (Fig. 4), which prevents reliable (P)EIS measurements on these samples. As a result, a direct comparison of the electronic properties of the surface layers across all three as-grown photoelectrodes is not feasible within the present study. Additionally, see Comment 1.6 of Reviewer 1.

Changes: We highlighted the results better in the main text on page 3: "In detail, we observe decreasing O/Ta ratios for Ta_3N_5(O) to Ta_3N_5(N) to Ta_3N_5(Ta), while the N/Ta simultaneously increases (Table S3). As a result, the composition of Ta_3N_5(Ta) is closest to the ideal stoichiometry of Ta_3N_5, while Ta_3N_5(O) is characterized by increased oxygen content and Ta_3N_5(N) by increased nitrogen content. "

We extended the discussion on page 4: "For GIWAXS measurements performed at an incident angle corresponding to a penetration depth of 3 nm, Ta_3N_5(Ta) still exhibits a clear polycrystalline structure with only minor differences between the surface and bulk. In contrast, the diffraction rings from surface-sensitive GIWAXS measurements are broadened for Ta_3N_5(N). Even more strikingly, Ta_3N_5(O) is characterized by the formation of an amorphous surface layer (Fig. 1d). These differences are further highlighted by incident angle-dependent GIWAXS maps (Fig. 1g–i), which reveal the structural evolution from the surface to the bulk of each film. While Ta_3N_5(Ta) and Ta_3N_5(N) exhibit rather crystalline surface layers, the structural differences between surface and bulk are more significant for the case of Ta_3N_5(O). In detail, Ta_3N_5(O) is characterized by a significantly thicker and more disordered surface layer, extending to greater than 10 nm below the surface, whereas the corresponding surface layer on Ta_3N_5(N) is limited to approximately 5 nm."

Comment 3.5: Are both surface-induced shallow oxygen vacancies and deep-level defects confined to the near-surface region? Since HF etching reduces the surface-layer thickness and removes the disordered surface layer in Ta_3N_5(O), all three samples should, in principle, exhibit more bulk-like characteristics after treatment. Why, then, does Ta_3N_5(O) continue to outperform the other two samples?

Answer: As a starting point for answering this insightful question, we note that common shallow defects in Ta_3N_5 are oxygen impurities on nitrogen sites, while typical deep defect states are nitrogen vacancies and/or reduced tantalum states. The shallow donor states introduced by oxygen can be beneficial for PEC performance since they increase the electrical conductivity. Moreover, we have previously shown that oxygen can passivate deep level defects, for example by reducing the nitrogen vacancy concentration, and can improve photoelectrode stability.[1] In contrast, nitrogen vacancies and reduced tantalum states act as recombination centers, leading to increased carrier trapping, reduced PEC performance, and stronger susceptibility to photocorrosion.

Importantly, our findings indicate that the three types of samples exhibit different bulk defect concentrations that arise from their different precursor-based synthesis routes. In particular, Ta_3N_5(O) contains a higher concentration of oxygen-related defects in the bulk, whereas Ta_3N_5(N) and Ta_3N_5(Ta) are characterized by comparatively lower oxygen content but higher concentrations of nitrogen-related defects, such as nitrogen vacancies and reduced tantalum states. As a result, Ta_3N_5(O) possesses a higher density of shallow donor states and a lower density of deep-level recombination centers, while Ta_3N_5(N) and Ta_3N_5(Ta) are characterized by higher concentrations of detrimental deep defects.

Upon HF treatment, the highly disordered surface layers are removed, but the differences in the bulk defect densities remain. Due to its larger oxygen impurity and lower deep level defect content, Ta_3N_5(O) is characterized by more favorable bulk properties, including enhanced conductivity, reduced recombination, and improved interfacial stability under ferrocyanide oxidation conditions due to kinetic stabilization of the interface.[1] Overall, the

observation of different PEC characteristics after HF-treatment is fully consistent with the differing bulk defect concentrations arising from the different precursors.

Changes: To better clarify the origin of these performance differences after HF etching, we extended the PEC discussion on page 8:

"Despite the removal of the disordered surface layer following HF etching, differences in PEC performance persist, arising from differences in bulk defect properties associated with the use of three different precursors. As previously reported,[1] Ta_3N_5(N) and Ta_3N_5(Ta) exhibit comparatively high concentrations of deep-level defects, whereas the higher oxygen content in Ta_3N_5(O) facilitates passivation of such states. While deep-level defects lead to rapid photocarrier trapping and recombination, limiting PEC performance, shallow oxygen donors can enhance the electronic conductivity and enable kinetic stabilization of the interface.[1] Consistent with these differences in bulk defect concentrations, Ta_3N_5(O) exhibits improved PEC performance and stability compared to Ta_3N_5(N) and Ta_3N_5(Ta) following HF etching (Fig. 5 and Fig. S6)."

Comment 3.6: HF etching leads to hydroxyl termination of the surface. Should this hydroxylation be considered as introducing a new type of defect, or is it better described as surface functionalization that primarily enhances hydrophilicity? If regarded as a defect, is it shallow or deep in nature? Moreover, what is the relationship between HF-induced hydrophilicity and the precursor-dependent shallow oxygen vacancies or deep-level defects?

Answer: We thank the reviewer for this insightful question. In our view, HF treatment should not be interpreted as introducing a new defect type in Ta_3N_5. Instead, our results suggest that it primarily acts as a surface modifier and passivator, removing the disordered, oxygen-rich surface layer and increasing hydrophilicity, likely by exposing a fresh surface and that is largely hydroxylated, with partially F termination. This interpretation is supported by multiple techniques: GIWAXS shows improved near-surface crystallinity, PDS indicates reduced sub-bandgap absorption, Mott–Schottky/EIS analysis shows a weaker influence of surface states, and PEIS/PEC measurements demonstrate improved interfacial charge transfer and stability. Together, these results indicate a reduction of detrimental surface states, rather than the formation of new defects. That said, we believe that additional studies of the electronic properties of differently terminated Ta_3N_5 surfaces would be of great interest, since the specific interface trap densities and their energetic distributions are not fully understood.

Contact-angle measurements show that all films become significantly more hydrophilic after HF treatment. At the same time, precursor-dependent differences persist after HF etching, with Ta_3N_5(N) and Ta_3N_5(Ta) retaining similarly low contact angles and Ta_3N_5(O) exhibiting slightly higher values. However, we note that hydrophilicity is primarily governed by surface chemistry (e.g., -OH termination) and surface morphology (e.g., roughness), whereas the

precursor-dependent defect concentrations primarily influence transport and recombination. Here, we note that Ta_3N_5(O) is characterized by a smoother surface with a finer grain structure, which could contribute to its somewhat larger contact angle relative to Ta_3N_5(N) and Ta_3N_5(Ta). While surface defect concentrations, such as vacancy sites and reconstructions, could impact the hydroxyl coverage and, thus, the hydrophilicity, identifying and quantifying such effects would represent a challenging study in itself. Therefore, we would prefer to refrain from speculating on these points.

Changes: To address this point, we improved the description of the experimental findings on page 11:
"Before and after HF treatment, Ta_3N_5(N) and Ta_3N_5(Ta) exhibit rather similar contact angles, whereas Ta_3N_5(O) exhibits slightly higher values."

Comment 3.7: Finally, the authors should provide surface morphology and structural characterization (e.g., TEM) of all three samples after HF etching to directly confirm the removal of the disordered surface layer and to assess any morphology changes induced by the treatment.

Answer: We thank the reviewer for the comment. To assess morphological changes after HF treatment, we recorded top-view SEM images before and after treatment. We do not observe any significant changes after HF treatment, indicating a homogenous surface modification. In addition, to directly visualize the removal of the surface layer, cross-sectional TEM was conducted on Ta_3N_5(O), which is expected to yield the greatest structural changes. These TEM measurements reveal a reduced thickness of the surface layer, consistent with the GIWAXS results, which show a reduced surface-layer thickness and improved crystallinity after HF for all samples.

Changes: We added top-view SEM images before and after HF-treatment as Fig. S5 in the supporting information and included the data in the discussion.

On page 8: ".., while the surface morphology remains similar after HF treatment (Fig. S5)."

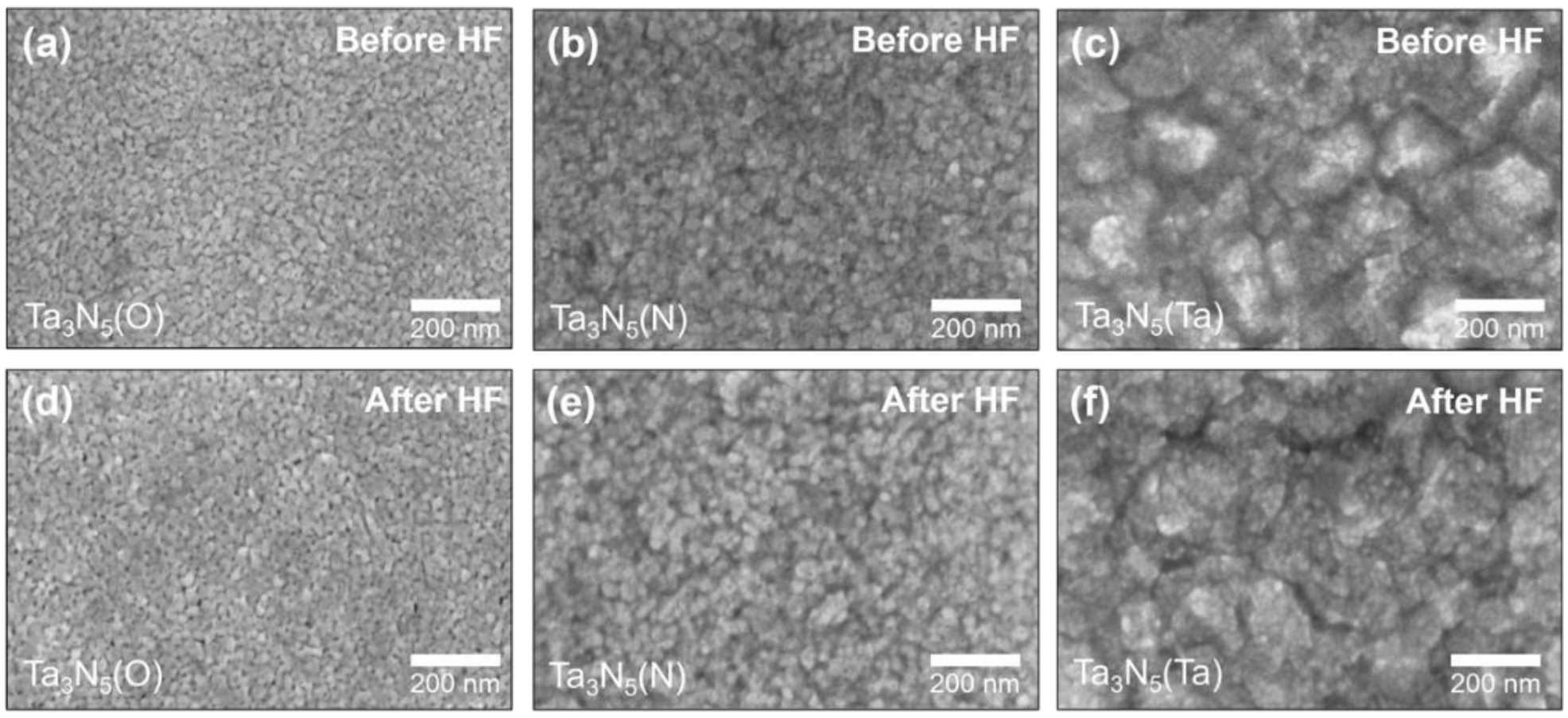

Figure S5. Top-view SEM topography images for all three Ta_3N_5 films before and after HF-treatment.

Furthermore, we added cross-sectional TEM images to the supporting information and included the data in our discussion.

On page 9: “To confirm the removal of the surface layer, complementary cross-sectional measurements were conducted on Ta_3N_5(O) after HF treatment (Fig. S8). Compared to as-grown Ta_3N_5(O), the amorphous layer is significantly reduced after HF etching and the near-surface crystallinity is improved.”

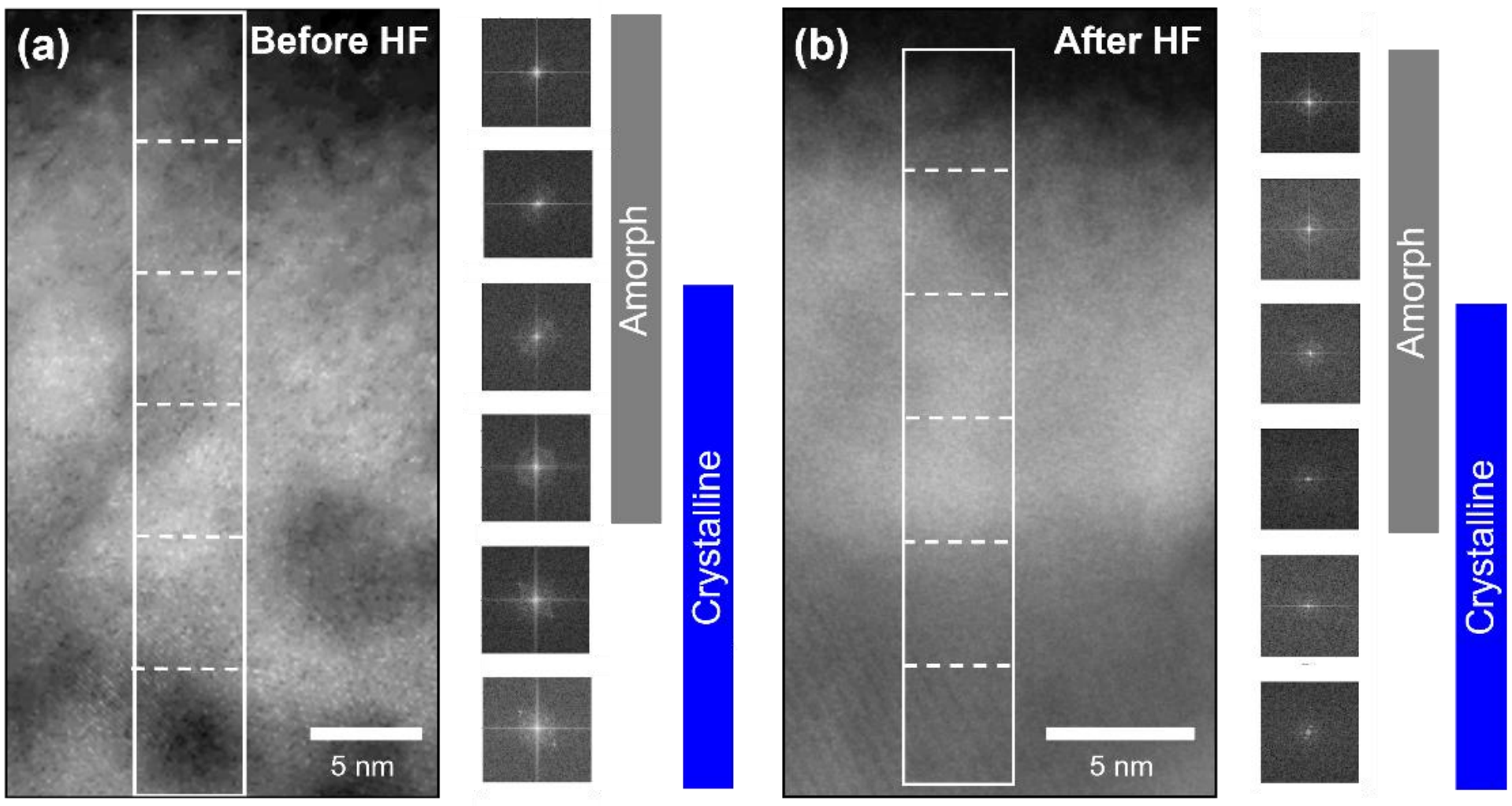

Figure S8. Cross-sectional HAADF STEM images for Ta_3N_5(O) films (a) before and (b) after HF-treatment.

References

1 L. M. Wolz, G. Grötzner, T. Rieth, L. I. Wagner, M. Kuhl, J. Dittloff, G. Zhou, S. Santra, V. Streibel, F. Munnik, I. D. Sharp and J. Eichhorn, *Adv. Funct. Mater.* 2024, **34**, 2405532.

2 T. Higashi, H. Nishiyama, V. Nandal, Y. Pihosh, Y. Kawase, R. Shoji, M. Nakabayashi, Y. Sasaki, N. Shibata, H. Matsuzaki, K. Seki, K. Takanabe and K. Domen, *Energy Environ. Sci.*, 2022, **15**, 4761–4775.

3 H.-C. Fu, W. Li, Y. Yang, C.-H. Lin, A. Veyssal, J.-H. He and S. Jin, *Nat. Commun*, 2021, **12**, 156.

4 D. Bae, G. Kanellos, G. M. Faasse, E. Dražević, A. Venugopal and W. A. Smith, *Commun. Mater.*, 2020, **1**, 17.

5 F. M. M. Francisco, P. Dias and A. Mendes, *J. Mater. Chem. A*, 2025, **13**, 14601–14611.

6 J. Fu, Z. Fan, M. Nakabayashi, H. Ju, N. Pastukhova, Y. Xiao, C. Feng, N. Shibata, K. Domen and Y. Li, *Nat. Commun.*, 2022, **13**, 729.

7 C. Shao, R. Chen, Y. Zhao, Z. Li, X. Zong and C. Li, *J. Mater. Chem. A*, 2020, **8**, 23274–23283.

8 J. Eichhorn, S. P. Lechner, C.-M. Jiang, G. F. Heunecke, F. Munnik and I. D. Sharp, *J. Mater. Chem. A*, 2021, **9**, 20653–20663.

9 D. R. Baer, K. Artyushkova, C. Richard Brundle, J. E. Castle, M. H. Engelhard, K. J. Gaskell, J. T. Grant, R. T. Haasch, M. R. Linford, C. J. Powell, A. G. Shard, P. M. A. Sherwood and V. S. Smentkowski, *J. Vac. Sci. Technol. A: Vacuum, Surfaces, and Films*, 2019, **37**, 031401.

10 D. I. Patel, T. Roychowdhury, V. Jain, D. Shah, T. G. Avval, S. Chatterjee, S. Bahr, P. Dietrich, M. Meyer, A. Thißen and M. R. Linford, *Surf. Sci. Spec.*, 2019, **26**, 016801.
11 K. Yasuda, *Quantum Beam Science*, 2020, **4**, 40.
12 H. Dosch, B. W. Batterman and D. C. Wack, *Phys. Rev. Lett.*, 1986, **56**, 1144–1147.
13 A. Hexemer and P. Müller-Buschbaum, *IUCrJ*, 2015, **2**, 106–125.
14 B. H. Frazer, B. Gilbert, B. R. Sonderegger and G. De Stasio, *Surf. Sci.*, 2003, **537**, 161–167.
15 D. Asakura, E. Hosono, Y. Nanba, H. Zhou, J. Okabayashi, C. Ban, P.-A. Glans, J. Guo, T. Mizokawa, G. Chen, A. J. Achkar, D. G. Hawthron, T. Z. Regier and H. Wadati, *AIP Advances*, 2016, **6**, 035105.
16 Z. Wei, M. Dubajic, C. Chosy, S. Kahmann and S. D. Stranks, *Nat. Rev. Methods Primers*, 2025, **5**, 37.
